# Lactate dehydrogenases promote glioblastoma growth and invasion via a metabolic symbiosis

**DOI:** 10.15252/emmm.202115343

**Published:** 2022-10-24

**Authors:** Joris Guyon, Ignacio Fernandez‐Moncada, Claire M Larrieu, Cyrielle L Bouchez, Antonio C Pagano Zottola, Johanna Galvis, Tiffanie Chouleur, Audrey Burban, Kevin Joseph, Vidhya M Ravi, Heidi Espedal, Gro Vatne Røsland, Boutaina Daher, Aurélien Barre, Benjamin Dartigues, Slim Karkar, Justine Rudewicz, Irati Romero‐Garmendia, Barbara Klink, Konrad Grützmann, Marie‐Alix Derieppe, Thibaut Molinié, Nina Obad, Céline Léon, Giorgio Seano, Hrvoje Miletic, Dieter Henrik Heiland, Giovanni Marsicano, Macha Nikolski, Rolf Bjerkvig, Andreas Bikfalvi, Thomas Daubon

**Affiliations:** ^1^ University Bordeaux, INSERM U1312, BRIC Pessac France; ^2^ University Bordeaux, INSERM, U1215 Neurocentre Magendie Bordeaux France; ^3^ University Bordeaux, CNRS, IBGC, UMR 5095 Bordeaux France; ^4^ Bordeaux Bioinformatic Center CBiB University of Bordeaux Bordeaux France; ^5^ Microenvironment and Immunology Research Laboratory, Medical Center University of Freiburg Freiburg Germany; ^6^ Department of Neurosurgery, Medical Center University of Freiburg Freiburg Germany; ^7^ Faculty of Medicine, University of Freiburg Freiburg Germany; ^8^ Translational NeuroOncology Research Group, Medical Center University of Freiburg Freiburg Germany; ^9^ Center of Advanced Surgical Tissue Analysis (CAST) University of Freiburg Freiburg Germany; ^10^ Freiburg Institute for Advanced Studies (FRIAS) University of Freiburg Freiburg Germany; ^11^ NorLux Neuro‐Oncology, Department of Biomedicine University of Bergen Bergen Norway; ^12^ Department of Oncology Luxembourg Institute of Health Luxembourg Luxembourg; ^13^ German Cancer Consortium (DKTK) Dresden Germany; ^14^ Core Unit for Molecular Tumor Diagnostics (CMTD) National Center for Tumor Diseases (NCT) Dresden Germany; ^15^ Animal Facility University Bordeaux Pessac France; ^16^ Institut Curie, INSERM U1021, CNRS UMR3347, Tumor Microenvironment Lab University Paris‐Saclay Orsay France; ^17^ Department of Pathology Haukeland University Hospital Bergen Norway; ^18^ German Cancer Consortium (DKTK), partner site Freiburg Freiburg Germany

**Keywords:** antiepileptic drug, energy metabolism, glioblastoma, invasion, lactate dehydrogenases, Cancer, Metabolism

## Abstract

Lactate is a central metabolite in brain physiology but also contributes to tumor development. Glioblastoma (GB) is the most common and malignant primary brain tumor in adults, recognized by angiogenic and invasive growth, in addition to its altered metabolism. We show herein that lactate fuels GB anaplerosis by replenishing the tricarboxylic acid (TCA) cycle in absence of glucose. Lactate dehydrogenases (LDHA and LDHB), which we found spatially expressed in GB tissues, catalyze the interconversion of pyruvate and lactate. However, ablation of both LDH isoforms, but not only one, led to a reduction in tumor growth and an increase in mouse survival. Comparative transcriptomics and metabolomics revealed metabolic rewiring involving high oxidative phosphorylation (OXPHOS) in the LDHA/B KO group which sensitized tumors to cranial irradiation, thus improving mouse survival. When mice were treated with the antiepileptic drug stiripentol, which targets LDH activity, tumor growth decreased. Our findings unveil the complex metabolic network in which both LDHA and LDHB are integrated and show that the combined inhibition of LDHA and LDHB strongly sensitizes GB to therapy.

## Graphical abstract

This study highlights the importance of metabolic symbiosis dependent on lactate and lactate dehydrogenase isoforms (LDHA and B) in glioblastoma development. Targeting both lactate dehydrogenases may be a novel potential therapeutic approach for targeting glioblastoma.

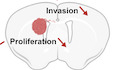

## Introduction

Glioblastoma (GB) is the most common malignant primary brain tumor in adults. The recently updated WHO classification integrates morphologic and genomic data, and classifies GB into a histologically and genetically defined group consisting of isocitrate‐dehydrogenase (IDH)‐wild‐type diffuse astrocytomas with TERT promoter mutation, or EGFR gene amplification, or +7/−10 chromosomal copy number changes (Louis *et al*, 2021). Major hallmarks of GB are high proliferation rate, pronounced angiogenesis, and local invasion (Giese *et al*, 2003). Glioblastoma is a highly heterogeneous tumor that exhibits astrocytic, oligodendrocytic, neural progenitor, and mesenchymal features (Neftel *et al*, 2019). The current treatment of GB consists in surgical tumor resection, followed by concomitant radio‐chemotherapy (Stupp *et al*, 2005). However, this strategy invariably leads to tumor recurrence due to the inability to fully eradicate invasive cells and results in a poor prognosis (Vollmann‐Zwerenz *et al*, 2020). This highlights the need for alternative therapeutic targets to improve tumor management and treatment. Glioblastoma invasion makes use of three types of brain structures: blood vessels, white matter tracts, and the interstitial space (Scherer, 1940). Hypoxia‐inducible factor 1α (HIF1α) expression induced by hypoxia or following anti‐angiogenic treatment significantly impacts GB invasion (Daubon *et al*, 2019b) and metabolism (Fack *et al*, 2015).

Unlike non‐neoplastic cells that often rely on mitochondrial oxidative phosphorylation (also known as “mitochondrial respiration”) to produce energy, a common property of cancer cells is enhanced glucose metabolism (Gatenby & Gillies, 2004). Under hypoxic pressure, cells commonly switch to a highly glycolytic metabolism and produce lactate. Under aerobic conditions, tumor cells can maintain this glycolytic metabolism, particularity known as the Warburg effect (Warburg, 1956), which is an efficient way to increase biomass production and therefore maintain a high proliferation rate and promote invasion (Gatenby & Gillies, 2004; Liberti & Locasale, 2016). Production of lactate, an end‐product of glycolysis, is catalyzed by lactate dehydrogenase (LDH), a tetrameric enzyme composed of two subunits, LDHA and LDHB, encoded by separate genes. In the human body, LDH exists in five different isozymes composed of four subunits, LDH1 is composed of only LDHB subunits, LDH5 of LDHA subunits, LDH2/3/4 are heterotetramers of both LDHA and LDHB (Drent *et al*, 1996). Depending on the lactate/pyruvate flux, LDHA and LDHB both catalyze the conversion of pyruvate into lactate or its retroconversion, both reactions being coupled to oxidoreduction of NAD^+^/NADH. This leads to increased lactate production (for LDHA) and consumption (for LDHB). Lactate and protons are then extruded from cells by monocarboxylate transporters (MCTs). Other subunits of LDH exist, such as LDHC and LDHD, but they are not highly expressed in brain tissue when compared to LDHA and LDHB (Bittar *et al*, 1996).

Many cancers display high LDHA levels (Altenberg & Greulich, 2004), which is associated with poor patient survival (Koukourakis *et al*, 2003, 2005). Several pre‐clinical studies have demonstrated that LDHA inhibition shows anti‐proliferative effects (Sheng *et al*, 2012; Xie *et al*, 2014). One proposed explanation is that LDHA inhibition induces mitochondrial reactive oxygen species (ROS) production and oxidative damage (Fantin *et al*, 2006; Le *et al*, 2010; Sheng *et al*, 2012). Moreover, several studies have shown that LDHA activity and lactate secretion promote invasion and metastasis (Goetze *et al*, 2011; Rizwan *et al*, 2013). Lactate has also been linked to *in vitro* migration of GB cells (Baumann *et al*, 2009; Colen *et al*, 2011; Seliger *et al*, 2013). LDHA has indeed been found at GB invasive borders of antiangiogenic‐treated tumors (Fack *et al*, 2015). IDH‐mutated gliomas, which are of better prognosis than IDH1wt gliomas, express lower levels of LDHA (Chesnelong *et al*, 2014). Of note, LDHB has not yet been explored in the GB context, unlike in uterine cancer where LDHB activity modulates autophagy (Brisson *et al*, 2016). However, no study to date has examined the role of both LDHA/LDHB in the development and invasiveness of GB. We, therefore, aimed to study the impact of LDHA and LDHB activity in GB development using patient‐derived GB stem‐like cells cultured as spheroids to maintain parental DNA genotype and phenotype while implanted into rodents (Wang *et al*, 2009).

We show herein that LDHA and LDHB expressions are spatially restricted in a GB stem‐like cell model (in invasive spheroids and *in vivo*) but also in patient samples, by analyzing spatial transcriptomics data (Ravi *et al*, 2022). GB stem‐like cells starved from carbon sources and fed with lactate can compensate for the absence of glucose to sustain the TCA cycle thus driving proliferation and invasion. Individual CRISPR‐Cas9 LDHA or LDHB knock‐outs have limited effects on glioblastoma development, while major biological adaptations are observed in double LDHA/B KO cells. Even under low O_2_ concentration, double LDHA/B KO cells still rely on TCA and mitochondrial respiration to support tumor growth, which could be further inhibited by irradiation. Finally, treatment with stiripentol (Diacomit®, Biocodex)—a known antiepileptic drug that also blocks LDH activity (Sada *et al*, 2015)—inhibits lactate production and decreases GB invasion and growth. Collectively, these results demonstrate a central role of LDHA and LDHB, which are regionally expressed, and lactate production in supporting GB energy metabolism which in turn has a major impact on GB invasion and growth.

## Results

### Expression of LDHA and LDHB isoforms is restricted to specific GB areas

**Figure 1:**
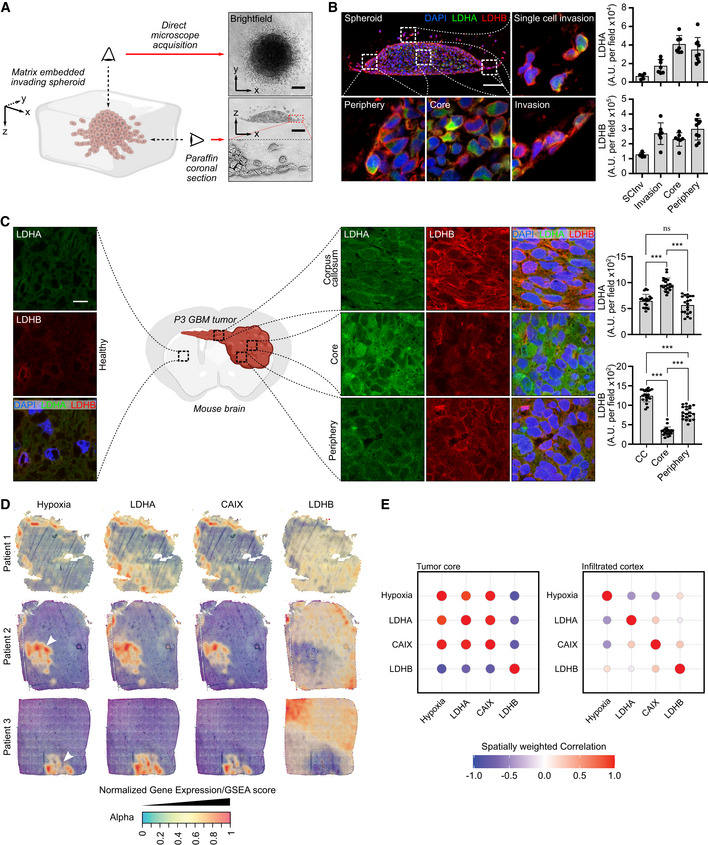
Regional expression of lactate dehydrogenases A and B in glioblastoma Left, 3D representation of an invading P3 spheroid embedded in a collagen I matrix. Right, upper view: brightfield microscope image, lower view: coronal section of an invading spheroid. Scale bar: 100 μm.Coronal section of a P3 spheroid embedded with paraffin and stained with DAPI (blue), LDHA (green), and LDHB (red). Magnification boxes show different areas as depicted in the main image. Quantification of LDHA and LDHB staining was performed on the spheroid areas as indicated in the graphs. Scale bar: 100 μm. Data are represented as mean ± s.d. (5–8 fields per area of interest, one representative spheroid of three independent invading spheroids).Coronal section of P3 tumors in a mouse brain, embedded with paraffin and stained with DAPI (blue), LDHA (green), and LDHB (red). Magnification shows areas as depicted in the central illustration. Quantification of LDHA and LDHB staining was performed on the tumor areas as indicated in the graphs. Scale bar: 15 μm. Data are represented as mean ± s.d. (20 fields per area of interest, one representative brain of two independently implanted mouse brains) and analyzed using one‐way ANOVA followed by Tukey's multiple comparisons test. LDHA: CC vs. Core, *P* < 0.0001; CC vs. Periphery, *P* = 0.18; Core vs. Periphery, *P* < 0.0001. LDHB: CC vs. Core, *P* < 0.0001; CC vs. Periphery, *P* < 0.0001; Core vs. Periphery, *P* < 0.0001.Surface plots of the expression of *LDHA*, *LDHB*, *CA9*, and gene set enrichment for hypoxia, in various stRNA samples (patients 1–3). The transparency of the spots is indicative of the expression/enrichment for the genes, with an additional layer of color based on expression/enrichment.Spatially weighted correlation analysis of *LDHA*, *LDHB*, *CAIX*, and gene set enrichment for hypoxia, separated between the tumor core and the infiltrating areas. Source data are available online for this figure.

We have previously reported regional expression of genes and proteins such as TSP1 in GB (Daubon *et al*, 2019a, 2019b). Here, a regional expression of the lactate dehydrogenase isoforms was first analyzed in spheroids invading a three‐dimensional (3D) collagen matrix, structures which recapitulate GB regional heterogeneity via oxygenation gradient (Guyon *et al*, 2020). Invasive tumor spheroids from P3 stem‐like cells were included in paraffin and LDHA and LDHB expressions were analyzed in coronal sections (Fig 1A). LDHA was found highly expressed in the central hypoxic area but also in some single invasive cells, while LDHB was highly expressed at spheroid borders and invasive areas (Fig 1B). Of note, only a few cells expressed both enzymes. Then, a regional expression of the LDHs was analyzed in a patient‐derived xenograft mouse model by immunohistochemistry, using the same P3 stem‐like cells. Notably, LDHA was found to be expressed mainly in the central hypoxic area of the tumor (Fig 1C; Appendix Fig S1A), colocalized with carbonic anhydrase IX (CAIX or CA9; Appendix Fig S1B), and some cells invading the corpus callosum (CC), while LDHB was prevalently expressed in peripheral tumor areas and cells invading the CC (Fig 1C). Thus, LDHA and LDHB had a distinct spatially restricted expression pattern in the tumor core but were found both expressed in invasive cells (Fig 1C; Appendix Fig S1A). By using a recently published database on spatial transcriptomics (Ravi *et al*, 2022), we analyzed the local expression of LDHA, LDHB, CAIX, or hypoxia signature in three different patient samples. LDHA and CAIX expression were restricted to the hypoxic core, and in some invasive cells (Fig 1D and E), LDHB was only expressed in invasive areas (Fig 1D and E). These spatial transcriptomics data strictly confirmed our results on the P3 tumor model.

*In silico* analysis on a single‐cell RNAseq database from invasive and central GB areas demonstrated a similar pattern, with preferential expression of *LDHA* in the central area and *LDHB* in the peripheral area albeit mRNA of both enzymes was found in a portion of invasive tumor cells (Appendix Fig S2A). When using data extracted from the IVYGAP database, distinct regional *LDHA* and *LDHB* expressions were observed, as well as a positive correlation between *LDHA* and the hypoxic transcription factor *HIF1Α* in invasive and microvascular areas (Appendix Fig S2B). On the contrary, *LDHB* and *HIF1A* expressions did not correlate (Appendix Fig S2B). When analyzed in the GB‐Tissue Cancer Genome Atlas (TCGA) dataset, LDHA was found to be a marker of poor prognosis. LDHB expression was, on the contrary, linked to a favorable prognosis (Appendix Fig S2C). The same tendency was observed when the expression of LDHA and LDHB was analyzed in low‐grade gliomas (Appendix Fig S2D). In summary, LDHA and LDHB expressions were spatially restricted to hypoxic or peripheral areas respectively, with a mixed pattern in invasive cells.

### Lactate modulates GB invasion by fueling energy metabolism pathways

**Figure EV1:**
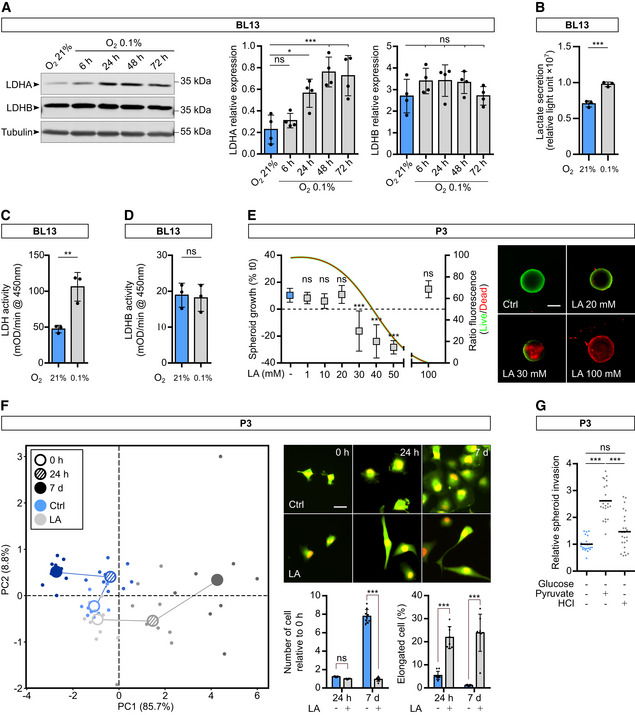
Independent stem‐like cell line BL13 confirms results obtained in P3 stem‐like cells (Extended data Fig 2A–H) Western blot analysis of LDHA and LDHB from BL13 cells upon exposure to 21% or 0.1% O_2_ during 6, 24, 48, and 72 h. The graphs represent densitometry quantification of the immunoblots normalized to tubulin (*n* = 4 independent experiments). Data are represented as mean ± s.d. and analyzed using one‐Way ANOVA following Dunnett's multiple comparisons test. LDHA: O_2_ 21% vs. O_2_ 0.1% 6 h, *P* = 0.81; O_2_ 21% vs. O_2_ 0.1% 24 h, *P* = 0.01; O_2_ 21% vs. O_2_ 0.1% 48 h, *P* = 0.0002; O_2_ 21% vs. O_2_ 0.1% 72 h, *P* = 0.0004. LDHB: O_2_ 21% vs. O_2_ 0.1% 6 h, *P* = 0.35; O_2_ 21% vs. O_2_ 0.1% 24 h, *P* = 0.34; O_2_ 21% vs. O_2_ 0.1% 48 h, *P* = 0.44; O_2_ 21% vs. O_2_ 0.1% 72 h, *P* > 0.99.Lactate secretion of BL13 cells exposed to 21% or 0.1% O_2_ measured by bioluminescent assay using a pro‐luciferin reductase substrate converted to luciferin in the presence of NADH (*n* = 3 independent experiments). Data are represented as mean ± s.d. and analyzed using unpaired *t*‐test: *P* = 0.0009.Enzymatic assays for the activity of LDH enzymes in BL13 cells (*n* = 3 independent experiments). Data are represented as mean ± s.d. and analyzed using unpaired *t*‐test: *P* = 0.008.Enzymatic assays for the activity of immune‐captured LDHB in BL13 cells (*n* = 3 independent experiments). Data are represented as mean ± s.d. and analyzed using unpaired *t*‐test: *P* = 0.81.P3 spheroid cytotoxicity assay was recorded over 24 h when incubated with or without lactic acid at different concentrations (1, 10, 20, 30, 40, 50, and 100 mM). Area of spheroids was measured at 0 and 24 h. Growth is represented as a percentage of the spheroid area when compared to time 0, and viability is estimated with live/dead fluorescence ratio at 24 h and represented as a fitted curve (*n* = 3 independent experiments, one experiment including 8–10 spheroids per condition). Data are represented as mean ± s.d. and growth at 72 h are analyzed using Kruskal–Wallis test followed by Dunn's multiple comparison test: Control vs. LA 1 mM, *P* > 0.99; Control vs. LA 10 mM, *P* = 0.27; Control vs. LA 20 mM, *P* > 0.99; Control vs. LA 30 mM, *P* < 0.0001; Control vs. LA 40 mM, *P* < 0.0001; Control vs. LA 50 mM, *P* < 0.0001; Control vs. LA 100 mM, *P* = 0.77. Images of representative spheroids in each condition (in green, calcein; in red, ethidium homodimer‐1). Scale bar: 250 μm.Principal component analysis of morphologic data on P3 cells incubated 7 days with or without lactate (20 mM). Cell number and morphology were measured at 0 h, 24 h, and 7 days (*n* = 3 independent experiments, one experiment including 2–3 independent cell dishes). Images of representative adherent cells in each condition (in green, GFP; in red, nuclear Tomato). Scale bar: 40 μm. The graphs represent quantification of the cell number and elongated cells (Aspect ratio > 2:5; *n* = 3). Data are represented as mean ± s.d. and analyzed using two‐way ANOVA followed by Sidak's multiple comparisons test. Number of cell: 24 h, *P* = 0.43; 7 days, *P* < 0.0001. Elongated cell: 24 h, *P* < 0.0001; 7 days, *P* < 0.0001.P3 spheroid invasion in collagen I gel incubated 24 h at 21% O_2_ and treated with 20 mM pyruvate or 1.5 mM HCl. Invasion rate is expressed as a fold change of the control (*n* = 3 independent experiments, one experiment including 6–8 spheroids per condition). Data are represented as mean ± s.d. and analyzed using Kruskal–Wallis test followed by Dunn's multiple comparison test: Control vs. Pyruvate, *P* < 0.0001; Control vs. HCL, *P* = 0.09; Pyruvate vs. HCL, *P* = 0.0002.

**Figure 2:**
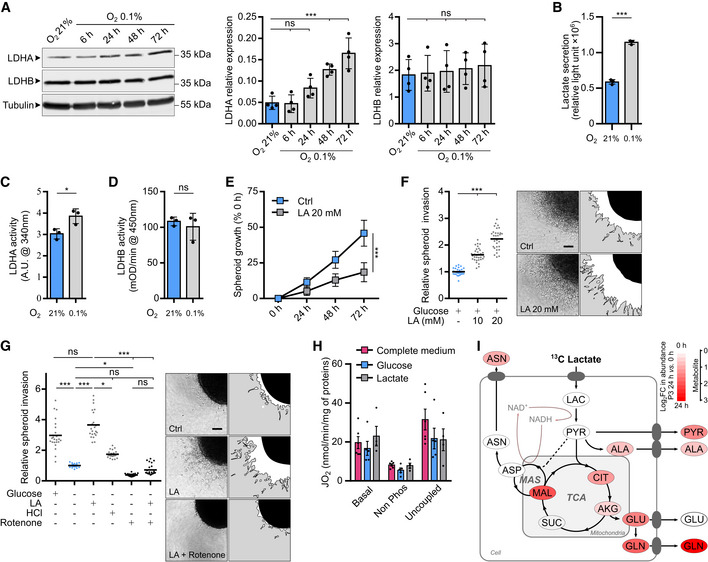
Hypoxia‐driven lactate fuels the TCA cycle and drives cell invasion Western blot analysis of LDHA and LDHB from P3 cells upon exposure to 21% or 0.1% O_2_ for 6, 24, 48, and 72 h. The graphs represent densitometry quantification of immunoblots normalized to tubulin (*n* = 4 independent experiments). Data are represented as mean ± s.d. and analyzed using one‐Way ANOVA following Dunnett's multiple comparisons tests. LDHA: O_2_ 21% vs. O_2_ 0.1% 6 h, *P* > 0.99; O_2_ 21% vs. O_2_ 0.1% 24 h, *P* = 0.15; O_2_ 21% vs. O_2_ 0.1% 48 h, *P* = 0.0009; O_2_ 21% vs. O_2_ 0.1% 72 h, *P* < 0.0001. LDHB: O_2_ 21% vs. O_2_ 0.1% 6 h, *P* > 0.99; O_2_ 21% vs. O_2_ 0.1% 24 h, *P* > 0.99; O_2_ 21% vs. O_2_ 0.1% 48 h, *P* = 0.97; O_2_ 21% vs. O_2_ 0.1% 72 h, *P* = 0.88.Lactate secretion of P3 cells exposed to 21 or 0.1% O_2_ was measured with a bioluminescent assay using a pro‐luciferin reductase substrate converted to luciferin in the presence of NADH (*n* = 3 independent experiments). Data are represented as mean ± s.d. and analyzed using unpaired *t*‐test: *P* < 0.0001.Enzymatic assays for the activity of LDHA in P3 cells (*n* = 3 independent experiments). Data are represented as mean ± s.d. and analyzed using unpaired *t*‐test: *P* = 0.03.Enzymatic assays for the activity of immune‐captured LDHB in P3 cells (*n* = 3 independent experiments). Data are represented as mean ± s.d. and analyzed using unpaired *t*‐test: *P* = 0.54.P3 spheroid growth was recorded over 72 h during incubation with or without 20 mM lactate. Area of spheroids was measured at 0, 24, 48, and 72 h, and growth is represented as a percentage of spheroid area compared to time 0 (*n* = 3 independent experiments, one experiment including 9–10 spheroids per condition). Data are represented as mean ± s.d., and growth at 72 h is analyzed using unpaired *t*‐test: *P* < 0.0001.P3 spheroid invasion in collagen I gel incubated 24 h at 21% O_2_ and incubated with 10 or 20 mM lactate (LA). Invasion rate is expressed as a fold change to the control (*n* = 4 independent experiments, one experiment including 6–8 spheroids per condition). Data are represented as mean and analyzed using Kruskal–Wallis test followed by Dunn's multiple comparisons test: Control vs. LA 10 mM, *P* < 0.0001; Control vs. LA 20 mM, *P* < 0.0001; LA 10 mM vs. LA 20 mM, *P* = 0.0009. Images of representative invasive spheroids in each condition. Scale bar: 100 μm.P3 spheroid invasion in collagen I gel incubated 24 h at 0.1% O_2_ and incubated with 20 mM lactate, 20 μM rotenone, and 1.5 mM HCl. Invasion rate is expressed as a fold change of the control (*n* = 4 independent experiments, one experiment including 6–8 spheroids per condition). Data are represented as mean and analyzed using Kruskal–Wallis test followed by Dunn's multiple comparisons tests: Control vs. Control + Glucose, *P* < 0.0001; Control vs. LA, *P* < 0.0001; Control vs. HCL, *P* = 0.11; Control vs. Rotenone, *P* = 0.01; Control vs. Rotenone + LA, *P* > 0.99; Control + Glucose vs. LA, *P* > 0.99; LA vs. HCL, *P* = 0.02; LA vs. Rotenone, *P* < 0.0001; LA vs. Rotenone + LA, *P* < 0.0001. Images of representative invasive spheroids in each condition. Scale bar: 100 μm.P3 cell mass‐specific respiration obtained by oxygraphy analysis. Cells were cultured in (i) complete medium or in medium without glucose and pyruvate supplemented with either (ii) 10 mM glucose or (iii) 20 mM lactate (*n* = at least four independent experiments). Data are represented as mean ± s.e.m. and analyzed using two‐way ANOVA followed by Tukey's multiple comparisons test (no statistical difference between conditions, *P*∈[0.6; 0.9999]). JO_2_ Basal: baseline mitochondrial respiration (oxygen consumption rate), JO_2_ non‐phosphorylating (non‐phos): minimal respiratory capacity, and JO_2_ uncoupled: maximal respiratory capacity.Metabolic changes of central carbon metabolism on P3 cells infused with [^13^C_3_] lactate for 24 h. Metabolites are labeled with a colored oval whose color corresponds to log_2_ fold changes between 24 h and 0 h (red, increase in 24 h) (*n* = 3 independent cell dishes). AKG, alpha‐ketoglutarate; ALA, alanine; ASN, asparagine; ASP, aspartate; CIT, citrate; GLN, glutamine; GLU, glutamate; LAC, lactate; MAL, malate; MAS, Malate–Aspartate Shuttle; PYR, pyruvate; SUC, succinate; TCA, TriCarboxylic Acid cycle. See also Appendix Figs S4 and S5. Source data are available online for this figure.

**Figure EV2:**
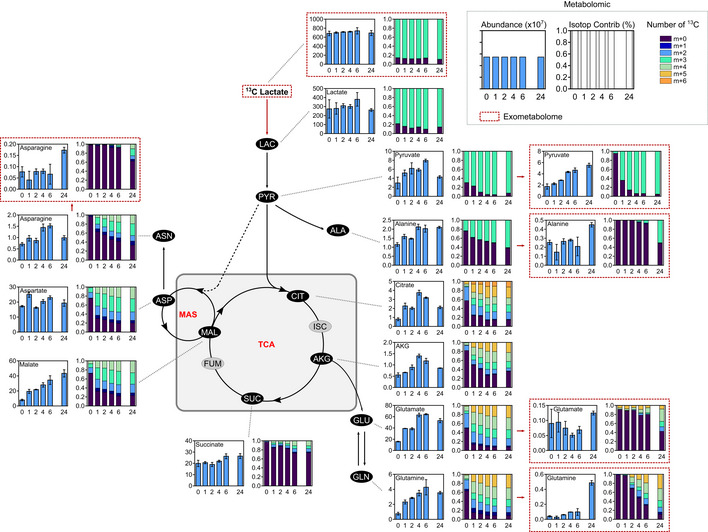
Metabolic tracing using [^13^C_3_] lactate (Extended data Fig 2I) P3 cells were infused during 0, 1, 2, 4, 6, and 24 h with [^13^C_3_] lactate at a concentration of 5 mM. Metabolites from cell extracts (endometabolome) or cell medium (exometabolome, red lines) measured by liquid chromatography–mass spectrometry (*n* = 3 independent cell dishes for each time point). Metabolite abundance of some intermediates of metabolic pathway of interest, data are represented as mean ± s.d. Quantification of the [^13^C_3_] lactate carbon incorporation into intermediates of the carbon metabolism (isotopologue contribution), data are represented as mean. *m* + 0 stands for the fraction of metabolite without ^13^Carbon and *m* + *n* (*n* > 0) stands for fraction of metabolite with *n* ^13^Carbon. The sum of (*m* + 0, *m* + 1, …, *m* + 10, …) equals to 1.

In physiological conditions, brain tissue is exposed to low levels of oxygen, that is physiological hypoxia which ranges from 0.5 to 7% O_2_ depending on the distance from blood vessels (Sakadžić *et al*, 2010). Local O_2_ concentration can be even lower in brain tumors, reaching around 0.1% O_2_ in the core area. We, therefore, investigated whether stem‐like cells (P3 or BL13 cells) in culture express LDHA and/or LDHB, and whether the expression of these enzymes can be modified by incubating cells at 0.1% O_2_. We observed that LDHA expression was upregulated after 48 h under hypoxic conditions (3× fold induction at 72 h), while LDHB expression did not change (Fig 2A for P3 cells and Fig EV1A for BL13 cells). Lactate production was also increased under hypoxia at 0.1% O_2_, correlating with higher LDHA activity in P3 and BL13 cells (Fig 2B–D and Fig EV1B–D). Lactate has been reported to sustain tumor growth via monocarboxylate transporters (MCT1/4) (Allen *et al*, 2016). We then tested the effect of lactate on tumor cell proliferation and invasion. To test a lactate‐induced cytotoxic effect, lactate was added to the medium of P3 cells, and cytotoxicity was measured. Lactate concentrations starting at 30 mM induced cytotoxicity at 24 h (Fig EV1E). The effect of non‐cytotoxic lactate concentrations was then tested and spheroid growth and invasion were measured. Spheroid growth was significantly inhibited by 20 mM lactate (Fig 2E), while cell invasion increased in the presence of lactate from 10 mM onwards (Fig 2F). Lactate treatment also induced changes in cell morphology, promoting an elongated cell shape, reminiscent of a mesenchymal phenotype (Fig EV1F). In absence of glucose, lactate by itself increased cell invasion (Fig 2G). To rule out the effect of acidification, we tested cell invasion at the same pH of lactate treatment (pH 6.8) by using HCl, and no effect was observed (Fig 2G). Moreover, rotenone, a respiratory chain complex I inhibitor, completely blocked lactate‐induced invasion, suggesting that lactate is fueling mitochondria activity to promote invasion (Fig 2G). Pyruvate, the main LDH substrate, was then tested in invasion experiments, and invasion rates were increased in comparison to control conditions but remained lower than under lactate stimulation (Figs EV1G and 2G). Of note, the respiration capacity of P3 cells was similar in a complete medium, with glucose or lactate (Fig 2H). Lactate consumption by GB mitochondrial activity was followed by [^13^C_3_] lactate infusion in glucose‐starved P3 cells, wherein labeled TCA intermediates (citrate, oxoglutarate, malate) and amino acids (alanine, glutamate, glutamine) were detected in the endometabolome but also in the exometabolome after 1 h (Figs 2I and EV2). The intracellular abundance of lactate and pyruvate was found unchanged during the experiment (Figs 2I and EV2), but the amount of [^13^C_3_] lactate and [^13^C_3_] pyruvate immediately reached their maximum level (Fig EV2). The appearance of [^13^C_2_] citrate from the beginning indicated a fast reaction from acetyl‐CoA, and higher isotopologues increased quickly after, thus indicating an active TCA cycle (Fig EV2). We challenged malate–aspartate shuttle (MAS) by using cycloserine as MAS inhibitor (MacDonald, 1995). This led to slight modifications of aspartate, glutamine, and glutamate concentrations at 4 h of treatment without impacting extracellular concentrations of glutamine (Appendix Fig S3). Main changes were observed for alanine labeling in endo‐ and exometabolome (Appendix Fig S3). These results shed light on the pathways involved in lactate metabolism of GB cells and are a direct demonstration that lactate is used as an energy source via the TCA cycle. Almost no labeling of amino acids not associated with the TCA cycle or gluconeogenesis was observed (not included in the analysis).

### Double LDHA/B KO impairs *in vitro* and *in vivo* GB growth and invasion

**Figure 3:**
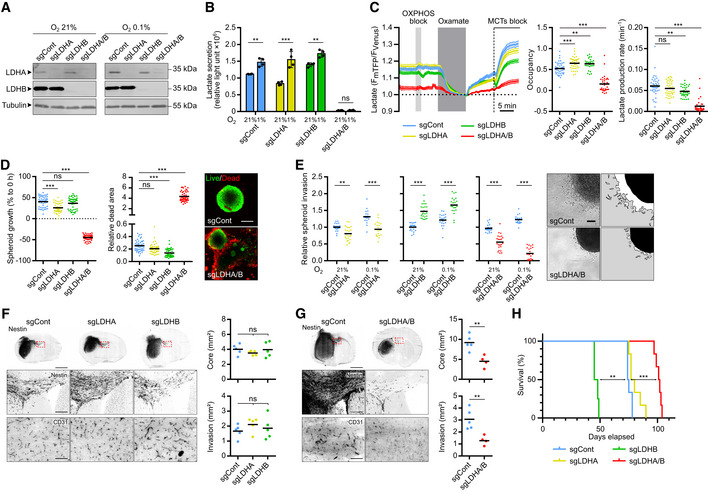
Double knockout of LDHA and LDHB impairs GB lactate metabolism, cell viability, and invasion AWestern blot analysis of LDHA and LDHB expression in P3 cells transduced with CRISPR‐Cas9 lentiviral vectors with scramble sequence (sgControl) or against LDHA (sgLDHA), LDHB (sgLDHB) or both (sgLDHA/B). Knockout (KO) cells were exposed to 21% or 1% O_2_ for 48 h.BLactate secretion of P3 cells KO for LDHA, LDHB, or both, exposed to 21% or 1% (*n* = 4 independent experiments). Data are represented as mean ± s.d. and analyzed using two‐way ANOVA followed by Tukey's multiple comparisons test: sgCont 21% vs. sgCont 1%, *P* = 0.002; sgLDHA 21% vs. sgLDHA 1%, *P* < 0.0001; sgLDHB 21% vs. sgLDHB 1%, *P* = 0.004; sgLDHA/B 21% vs. sgLDHA/B 1%, *P* > 0.99; sgCont/sgLDHA/sgLDHB vs. sgLDHA/B, *P* < 0.0001.CIntracellular lactate level analyzed with a fluorescent biosensor in P3 cells (control, KO for LDHA, LDHB, or both). *Left*, lactate level monitored in basal condition, followed by sequential exposure to OXPHOS block (5 mM sodium azide), 6 mM oxamate, and MCTs blockers (1 μM AR‐C1558585 + 1 mM diclofenac). The response to oxamate and MCTs blockers was used to determine, *Middle*, the basal lactate level (expressed as biosensor occupancy) and, *Right*, the lactate production rate (*n* = 4, 33–44 cells analyzed per condition). Data are represented as mean ± s.e.m. (*Left*) or as mean (*Middle* and *Right*) and analyzed using one‐way ANOVA following Tukey's multiple comparisons test. Occupancy: sgCont vs. sgLDHA, *P* = 0.001; sgCont vs. sgLDHB, *P* = 0.005; sgCont vs. sgLDHA/B, *P* < 0.0001. Lactate production: sgCont vs. sgLDHA, *P* = 0.32; sgCont vs. sgLDHB, *P* = 0.01; sgCont vs. sgLDHA/B, *P* < 0.0001. See Materials and Methods for analysis details and see also Fig EV3D.D
*Left*: P3 spheroid growth recorded over 1 week at 1% O_2_ (*n* = 3 independent experiments, one experiment including 16 spheroids per condition). Data are represented as mean and analyzed using Kruskal–Wallis test followed by Dunn's multiple comparisons test. Spheroid growth: sgCont vs. sgLDHA, *P* = 0.0003; sgCont vs. sgLDHB, *P* = 0.78; sgCont vs. sgLDHA/B, *P* < 0.0001. Dead area: sgCont vs. sgLDHA, *P* = 0.19; sgCont vs. sgLDHB, *P* < 0.0001; sgCont vs. sgLDHA/B, *P* < 0.0001. *Right*: Viability of spheroids at 1 week, incubated with calcein (green) or ethidium homodimer‐1 (red). Scale bar: 200 μm.EP3 spheroid invasion in collagen I gel, incubated 24 h at 21% or 0.1% O_2_. Invasion rate is expressed as fold change from controls (*n* = 4 independent experiments, one experiment including 6–8 spheroids per condition). Data are represented as mean and analyzed using two‐way ANOVA followed by Tukey's multiple comparisons test: sgCont 21% vs. sgLDHA 21%, *P* = 0.003; sgCont 0.1% vs. sgLDHA 0.1%, *P* < 0.0001; sgCont 21% vs. sgLDHB 21%, *P* < 0.0001; sgCont 0.1% vs. sgLDHB 0.1%, *P* < 0.0001; sgCont 21% vs. sgLDHA/B 21%, *P* < 0.0001; sgCont 0.1% vs. sgLDHA/B 0.1%, *P* < 0.0001. Images of representative of control or LDHA/B KO spheroids. Scale bar: 100 μm.F, GA first cohort of mice was sacrificed when one mouse reached a limit point, brains were extracted, snap‐frozen in liquid nitrogen, sliced, and stained. For the second cohort, each mouse was sacrificed when it reached a limit point allowing the draw of survival curves. *Left*: Immunofluorescence staining of Nestin (*top* and *middle*) and CD31 (*bottom*) of control and LDHA/B KO P3 tumor section. Scale bars: 2 mm (*top*), 250 μm (*middle*) and 100 μm (*bottom*). *Right*: Graphs represent tumor core and invasion area of control and LDHA/B KO P3 tumors in mm^2^ (*n* = 5 mice per group, average of 5–6 sections per tumor). Data are represented as mean. For (F), data are analyzed using one‐way ANOVA followed by Dunnett's multiple comparisons test. Core: sgCont vs. sgLDHA, *P* = 0.48; sgCont vs. sgLDHB, *P* = 0.99. Invasion: sgCont vs. sgLDHA, *P* = 0.40; sgCont vs. sgLDHB, *P* = 0.81. For G, data are analyzed using unpaired *t*‐test. Core: sgCont vs. sgLDHA/B, *P* = 0.005. Invasion: sgCont vs. sgLDHA/B, *P* = 0.01.HKaplan–Meier survival curves of xenotransplanted mice with P3 cells KO for LDHA (yellow), LDHB (green), LDHA/B (red), or control (blue) (*n* = 8 mice per group). Data are analyzed using Log‐rank (Mantel‐Cox) test: sgCont vs. sgLDHA, *P* = 0.06; sgCont vs. sgLDHB, *P* = 0.001; sgCont vs. sgLDHA/B, *P* = 0.0007. Source data are available online for this figure.

**Figure EV3:**
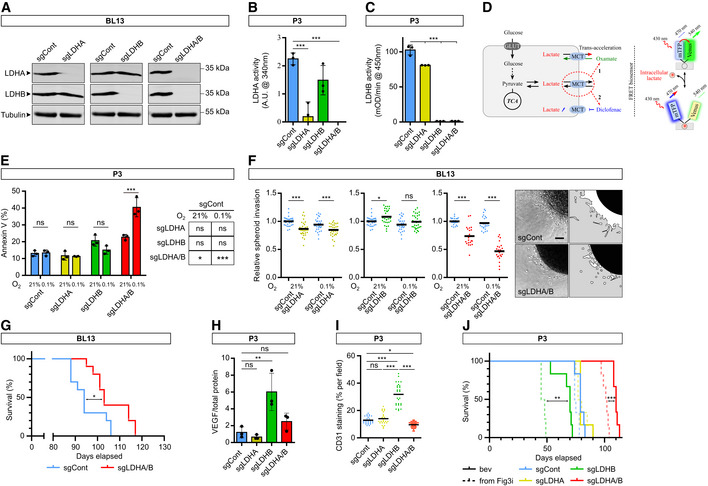
LDHA/B KO in BL13 cells and bevacizumab treatment in P3 tumors (Extended data Fig 3) Western blot analysis of LDHA and LDHB from BL13 cells knockout by CRISPR‐Cas9 lentiviral vectors against LDHA, LDHB, or both, and upon exposure to 21% O_2_.Enzymatic assays for the activity of LDHA in P3 cells (*n* = 3 independent experiments). Data are represented as mean ± s.d. and analyzed using one‐way ANOVA followed by Dunnett's multiple comparison test: sgCont vs. sgLDHA, *P* = 0.0005; sgCont vs. sgLDHB, *P* = 0.1; sgCont vs. sgLDHA/B, *P* < 0.0001.Enzymatic assays for the activity of immune‐captured LDHB in P3 cells (*n* = 3 independent experiments). Data are represented as mean ± s.d. and analyzed using one‐way ANOVA followed by Dunnett's multiple comparison test: sgCont vs. sgLDHA, *P* = 0.0002; sgCont vs. sgLDHB, *P* < 0.0001; sgCont vs. sgLDHA/B, *P* < 0.0001.Schematic representation of the intracellular lactate level monitoring with a fluorescent biosensor. The presence of the lactate changes the conformation of the biosensor and fluorescence emission. Known as an accelerated‐exchange transport (trans‐acceleration), oxamate was used to quickly release the lactate out of the cells for the determination of the lactate basal level. Then, diclofenac was used to block the lactate transporter for the quantification of the lactate production rate.Cells incubated during 48 h at 21% or 0.1% O_2_, labeled with Annexin‐V FITC, and analyzed by cytometry (*n* = 3 independent experiments). Data are represented as mean ± s.d. and analyzed using two‐way ANOVA followed by Tukey's multiple comparison test: sgCont 21% vs. sgCont 0.1%, *P* > 0.99; sgLDHA 21% vs. sgLDHA 0.1%, *P* > 0.99; sgLDHB 21% vs. sgLDHB 0.1%, *P* = 0.3, sgLDHA/B 21% vs. sgLDHA/B 0.1%, *P* < 0.0001. Table of statistical comparisons of Annexin‐V signal in sgLDHA, sgLDHB and sgLDHA/B cells with respective control (either 21 or 0.1% O_2_): sgCont 21% vs. sgLDHA 21%, *P* > 0.99; sgCont 0.1% vs. sgLDHA 0.1%, *P* = 0.98; sgCont 21% vs. sgLDHB 21%, *P* = 0.07; sgCont 0.1% vs. sgLDHB 0.1%, *P* > 0.99; sgCont 21% vs. sgLDHA/B 21%, *P* = 0.01; sgCont 0.1% vs. sgLDHA/B 0.1%, *P* < 0.0001.BL13 spheroid invasion in collagen I gel incubated 24 h at 21 or 0.1% O_2_. Invasion rate is expressed as a fold change of the control (*n* = 4 independent experiments, one experiment including 7–8 spheroids per condition). Data are represented as mean and analyzed using two‐way ANOVA test followed by Tukey's multiple comparison test: sgCont 21% vs. sgLDHA 21%, *P* < 0.0001; sgCont 0.1% vs. sgLDHA 0.1%, *P* = 0.001; sgCont 21% vs. sgLDHB 21%, *P* = 0.03; sgCont 0.1% vs. sgLDHB 0.1%, *P* = 0.29; sgCont 21% vs. sgLDHA/B 21%, *P* < 0.0001; sgCont 0.1% vs. sgLDHA/B 0.1%, *P* < 0.0001. Images of representative invasive sgControl or sgLDHA/B spheroids. Scale bar: 100 μm.Kaplan–Meier survival curves of xenotransplanted mice with BL13 cells KO for LDHA/B (red) or control (blue) (*n* = 10 mice per group). Data are analyzed using log‐rank (Mantel‐Cox) test: *P* = 0.02.Supernatants were collected from each cell line and analyzed by using ELISA to detect VEGF (*n* = 3 independent experiments). Data are represented as mean ± s.d. and analyzed using one‐way ANOVA followed by Dunnett's multiple comparison test: sgCont vs. sgLDHA, *P* = 0.91; sgCont vs. sgLDHB, *P* = 0.004; sgCont vs. sgLDHA/B, *P* = 0.5.Tumor blood vessels were stained with anti‐CD31 antibodies and CD31 staining area was calculated over tumor area (related to Figs 3F and G). Data are represented as mean and analyzed using one‐way ANOVA followed by Tukey's multiple comparison test: sgCont vs. sgLDHA, *P* = 0.81; sgCont vs. sgLDHB, *P* < 0.0001; sgCont vs. sgLDHA/B, *P* = 0.034; sgLDHA vs. sgLDHB, *P* < 0.0001; sgLDHB vs. sgLDHA/B, < 0.0001.Kaplan–Meier survival curves complement of Fig 3H where xenotransplanted mice with LDHA/B KO P3 spheroids were treated by bevacizumab (*n* = 8 mice per group). Data are analyzed using log‐rank (Mantel‐Cox) test: sgLDHB vs. sgLDHB + bevacizumab, *P* = 0.001; sgLDHA/B vs. sgLDHA/B + bevacizumab, *P* = 0.0005.

We then decided to investigate the LDH function via knockout (KO). We transduced P3 and BL13 cells with lentiviral CRISPR‐Cas9 constructs to fully abolish LDHA and/or LDHB expressions (sgControl, sgLDHA, sgLDHB, and sgLDHA/B). KO was validated by Western blot in single LDHA or LDHB KO cells, and in double LDHA/B KO cells at 21 or 0.1% O_2_ (Figs 3A and EV3A). Next, we performed functional experiments to further validate the KO strategy. Lactate secretion was only abolished on double LDHA/B KO cells, but was increased in LDHB KO when compared to the control (Fig 3B). LDHA activity was absent in LDHA KO and double LDHA/B KO cells but no differences were observed between control and LDHB KO cells (Fig EV3B). LDHB activity was not detected in LDHB or LDHA/B KO cells and slightly decreased in LDHA KO cells (Fig EV3C). Next, we stably transduced P3 cells with the lactate‐sensitive FRET biosensor to detect intracellular lactate levels (Fig EV3D; San Martín *et al*, 2013). Basal lactate concentrations were higher in control, LDHA, or LDHB KO cells than in double LDHA/B KO cells (Fig 6C). Adding oxamate a potent LDH inhibitor that also induces lactate efflux by MCT trans‐acceleration (Contreras‐Baeza *et al*, 2019), followed by a MCT blocker cocktail (diclofenac and AR‐C155858) unveil that basal lactate concentrations were higher in control, LDHA, or LDHB KO cells compared to double LDHA/B KO cells (Fig 3C). Spheroid growth was then monitored over 1 week at 1% O_2_ and showed that double LDHA/B KO strongly reduced spheroid growth, unlike LDHA KO, which moderately decreased spheroid growth and LDHB KO, which had no effect (Fig 3D, left panel). This was associated with a strong ethidium homodimer‐1 staining to visualize apoptotic events (Fig 3D, right panel). This cell death signature was also supported by high Annexin‐V staining in double KO cells detected by cytometry (Fig EV3E).

The invasion capacity was analyzed in all cell lines, and a moderate but significant decrease in invasion was observed in LDHA KO in both P3 (Fig 3E) and BL13 cells (Fig EV3F). Nevertheless, LDHB KO P3 but not LDHB KO BL13 cells had higher invasive capacities (Figs 3E and EV3F). For LDHA/B KO cells, under 0.1% O_2_, the invasive ability dropped by 75% for P3 cells (Fig 3E) and 50% for BL13 cells (Fig EV3F).

Spheroids from single and double LDHA/B KO cells were injected into immunodeficient mice to evaluate *in vivo* tumor development and survival. No histopathological differences between control and single LDH KO (LDHA KO or LDHB KO) tumors were observed (Fig 3F). However, double LDHA/B KO tumors were much smaller and less invasive than control tumors (Fig 3G), which correlated with an increase in mouse survival in both, the P3 (Fig 3H) and the BL13 model (Fig EV3G).

Only a small but significant increase in mouse survival was observed in the LDHA KO group, and, surprisingly, a drastic decrease in survival in the LDHB KO group (Fig 3H); the latter was most likely due to hemorrhages at the tumor site. Vascular endothelial growth factor (VEGF), the main inducer of neoangiogenesis by tumor cells, was quantified by ELISA, and an increase was only observed in LDHB KO cells (Fig EV3H), correlating with higher vascular coverage in LDHB KO tumor (Fig EV3I). The VEGF inhibitor bevacizumab (bev) was then injected in mice bearing single or double LDHA/LDHB KO P3 tumors to reverse this phenotype. An increase in survival was observed when mice implanted with LDHB KO tumors were treated with bev, reaching a similar survival curve to control animals (Fig EV3J). Increased survival was also seen in mice with double LDHA/B KO tumors, while no difference was observed in mice with control or LDHA KO tumors (Fig EV3J).

### Metabolic switch in double LDHA/B KO cells under hypoxia delineates vulnerabilities

**Figure 4:**
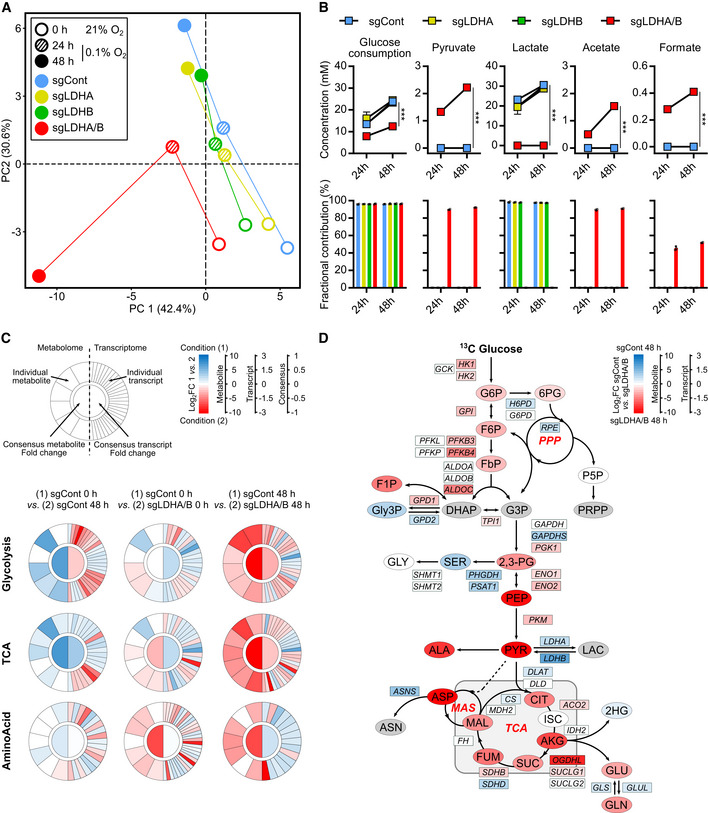
Correlation between transcriptomic and metabolomic analysis reveals dysregulated metabolic pathways in double LDHA/B knockout Control or LDH KO P3 cells were infused 0, 24, and 48 h with [^13^C] glucose at 0.1% O_2_ (*n* = 3 independent cell dishes).
Principal component analysis of metabolomic data from cell extracts.Metabolites from cell medium. *Top*, concentration of metabolite consumed ([^13^C] glucose) or secreted (pyruvate, lactate, acetate, formate) by cells. Data are represented as mean ± s.d. and analyzed using two‐way ANOVA followed by Sidak's multiple comparisons test: for all metabolites, sgCont/sgLDHA/sgLDHB vs. sgLDHA/B, *P* < 0.0001. *Bottom*, amount of labeled isotopes relative to the total amount of this element, expressed as a percentage (fractional contribution) Data are represented as mean ± s.d.Circular metabologram illustrating metabolic and transcriptomic differences in metabolite pathways between LDH KO P3 cells. The metabologram is divided in two parts, the left corresponds to metabolomic analysis and the right to the transcriptomic analysis. The outer circle corresponds to the log_2_ fold change for each metabolite (*left*) and transcripts (*right*). The central circle displays the average fold change of all analytes.Metabolic changes of central 13‐labeled‐carbon metabolism in LDH KO P3 cells infused with [^13^C_6_] glucose at 1% O_2_ for 48 h. Metabolites are labeled with a colored oval and enzyme transcripts with a colored square, colors correspond to the log_2_ fold changes between sgControl and sgLDHA/B (blue, increase in sgControl; red, increase in sgLDHA/B; gray, not measured or not computable). For legend, see also Fig 2I; 2,3‐PG: 2,3‐Phosphoglycerate; 2HG: 2‐Hydroxyglutarate; 6PG: 6‐Phosphogluconate; DHAP: Dihydroxyacetone Phosphate; F1P: Fructose‐1‐Phosphate; F6P: Fructose‐6‐Phosphate; FbP: Fructose‐bisPhosphate; G3P: Glyceraldehyde‐3‐Phosphate; G6P: Glucose‐6‐Phosphate; GLY: Glycine, SER: Serine; Gly3P: Glycerol‐3‐Phosphate; P5P: Pyridoxal‐5‐Phosphate; PEP: Phosphoenolpyruvate; PPP: Pentose Phosphate Pathway; PRPP: Phosphoribosylpyrophosphate. Source data are available online for this figure.

To trace metabolic fluxes at 0.1% O_2_, cells were starved and infused with [^13^C_6_] glucose for 24 or 48 h. Principal component analysis (PCA) of endometabolome, defining global regulation of intracellular metabolites, showed that the metabolism of double LDHA/B KO cells switched after 48 h at 0.1% O_2_ when compared to control or single KO tumors (Fig 4A). Exometabolome of double LDHA/B KO cells strongly differed from other cells by a decrease in glucose consumption and an absence of lactate production (Fig 4B). In addition, pyruvate and its incompletely catabolized derivatives (acetate and formate) were only secreted in double LDHA/B KO cells (Fig 4B).

RNA sequencing was then performed on the same cells under 21% or 0.1% O_2_ and confronted with metabolomics data. Metabolograms were used to integrate transcriptomics and metabolomics data as previously described (Hakimi *et al*, 2016). The changes of each metabolite/transcript pair were determined, and average metabolome/transcriptome variations of metabolic pathways were visualized by comparing two conditions (Fig 4C). Based on global results shown above (Fig 4A), metabolograms were built by analyzing total metabolite abundances in P3 control cells at 21% or 0.1% O_2_ (Fig 4C, left panels; Appendix Fig S4), and compared to P3 double LDHA/B KO cells at 21% (Fig 4C, middle panels; Appendix Fig S4) or 0.1% O_2_ (Fig 4C, right panels, and Appendix Fig S4). When changes in consensus gene expression were analyzed, differences in glycolysis or TCA cycle for all comparisons were seen, but only negligible variations for amino acid synthesis (Fig 4C). Significant variations in the metabolites were observed for most of the comparisons (Fig 4C). For P3 control cells, the majority of transcripts related to glycolysis increased in expression at 0.1% O_2_ when compared to 21% O_2_ (18 up, 12 down). In contrast, the majority of metabolites significantly decreased at 0.1% O_2_ (0 up, 4 down; Fig 4C, left panels). Metabolograms were heterogeneous when comparing changes between P3 control and double LDHA/B KO cells at (1) 21% and (2) at 0.1% O_2_ (Fig 4C, middle and right panels). P3 double LDHA/B KO cells had a strongly modified metabolism, as seen by the increase in all metabolites and transcripts related to glycolysis and oxidative phosphorylation, particularly under 0.1% O_2_ (Fig 4C).

**Figure EV4:**
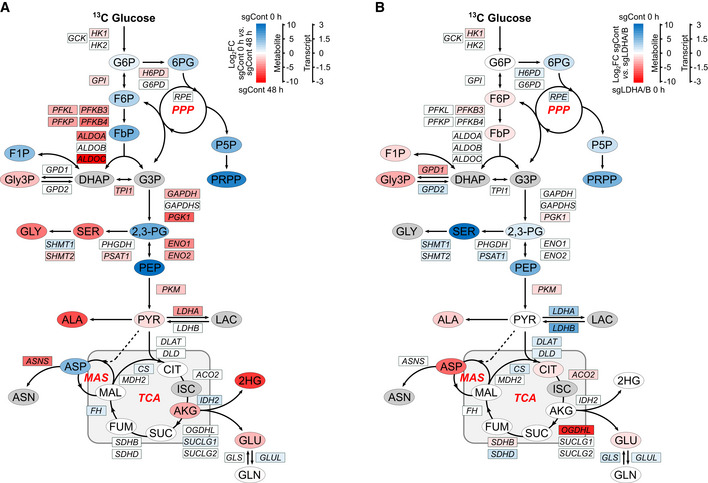
P3 sgControl RNAseq/metabolomic adaptation profiles to hypoxia and basal differences between P3 sgControl and P3 sgLDHA/B cells (Extended data Fig 4) Metabolic changes of central 13‐labeled‐carbon metabolism when knock‐out P3 cells are infused with [^13^C_6_] glucose. Metabolites are labeled with colored oval and enzyme transcripts with colored square, colors correspond to the log_2_ fold changes between:
sgCont 0 h and sgCont 48 h at 0.1% O_2_ (blue, increase in sgCont 0 h; red, increase in sgCont 48 h; gray, not measured or not computable).sgCont 0 h and sgLDHA/B 0 h (blue, increase in sgCont 0 h; red, increase in sgLDHA/B 0 h; gray, not measured or not computable). For details, see also Figs 4 and EV5.

**Figure EV5:**
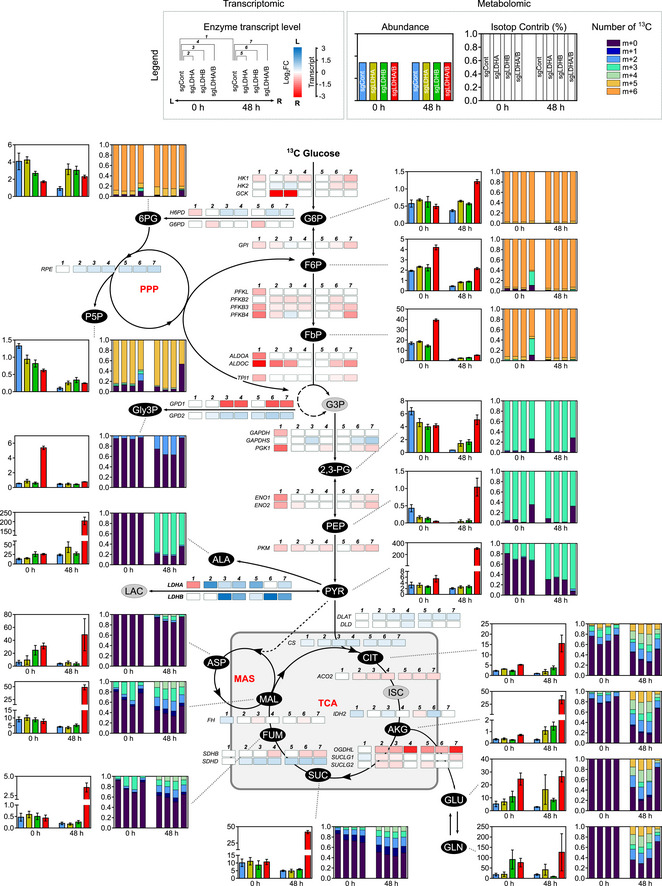
Metabolic tracing using [^13^C_6_] glucose (Extended data Fig 4) P3 sgControl, sgLDHA, sgLDHB, and sgLDHA/B were infused during 0, 24, and 48 h at 0.1% O_2_ with [^13^C_6_] glucose. Metabolites from cell extracts (endometabolome) were measured by liquid chromatography–mass spectrometry (*n* = 3 independent cell dishes for each condition and time point) and transcripts by RNA sequencing. Abundance and isotopologue contribution of all metabolites from glucose metabolism are shown. For abundance, data are represented as mean ± s.d., and for isotopologue contribution, data are represented as mean. *m* + 0 stands for the fraction of metabolite without ^13^Carbon and *m* + *n* (*n* > 0) stands for fraction of metabolite with *n* ^13^Carbon. For example, *m* + 5 corresponds to a metabolite with 5 labeled ^13^Carbon. The sum of (*m* + 0, *m* + 1, …, *m* + 10, …) equals 1. For details, see also Figs 4 and EV4.

Since metabolograms are based on the total metabolite abundances, metabolite abundance was calculated after [^13^C_6_] glucose labeling using the fractional contribution and was incorporated into the central pathway of the carbon metabolic network map obtained from previous comparisons (Fig 4C). To explore the relationship between transcriptomic and metabolomic data, transcript levels of metabolism‐related genes were incorporated into the central carbon metabolic network pathway map (Figs 4D and EV4A and B). P3 control cells incubated 48 h under 0.1% O_2_ showed a global increase in glycolytic enzyme expression with a concomitant reduction of related metabolites (Fig EV4A). At 21% O_2_, the difference between P3 double LDHA/B KO versus control cells was non‐significant (Fig EV4B). Under 0.1% O_2_, most [^13^C_6_] metabolites from the glycolytic and oxidative phosphorylation pathways accumulated in double KO LDHA/B cells compared to control cells (Fig 4D). Fourteen transcripts from the glycolytic pathway (HK1, HK2, GPI, PFKB3, PFKB4, ALDOC, TPI1, PGK1, ENO1, ENO2, and PKM) and the Krebs cycle (ACO2, SUCLG1, and SDHB) were upregulated, while six were downregulated (GAPDHS, LDHA, LDHB, DLAT, SDHD, CS; Fig 4D). These findings indicate that, under hypoxia, double LDHA/B KO deregulates cell metabolism at multiple levels. Global transcripts were increased in P3 control cells incubated at 0.1% O_2_ when compared to 21% O_2_ (Appendix Fig S5, upper panels; Fig EV5), impacting metabolism and, in particular, glycolysis (HK2, ENO1, ENO2, GAPDH, PKM, LDHA). At 21% O_2_, major biological pathways are related to membrane and organelle dynamics during cell division and cell cycle regulation; on the contrary, in more unfavorable conditions at 0.1% O_2_, pathways are involved in low oxygen adaptation and maintenance of energy production (Appendix Fig S5, upper panels; Fig EV5). Hypoxic stress has decreased the nucleotide pool (Hisanaga *et al*, 1986). Indeed, the abundance of nucleotides was lower while isotopologue contribution was enriched, suggesting a continuous but limited nucleotide synthesis (Appendix Fig S6). Under 0.1% O_2_, the transcription profiles were more enriched in double LDHA/B KO cells when compared to P3 sgControl cells (Appendix Fig S5, lower panels; Fig EV5). This was also seen at 21% O_2_ (Appendix Fig S5, middle panels, Fig EV5). The differences in the amount and isotopologue contribution between control and double KO LDHA/B cells were less pronounced at 21% O_2_. In hypoxia, the abundance of nucleotide monophosphates (AMP, GMP, and UMP) was also higher in double KO LDHA/B cells (Appendix Fig S6). The fractional contribution of purines and pyrimidines mainly consisted of *m* + 5 labeling in double KO LDHA/B cells, whereas *m* + 5, *m* + 7, and *m* + 8 labeling were seen in control cells (Appendix Fig S6).

### Double LDHA/B KO cells reorganize their mitochondrial respiratory chain to support growth under 0.1% O_2_ concentration

**Figure 5:**
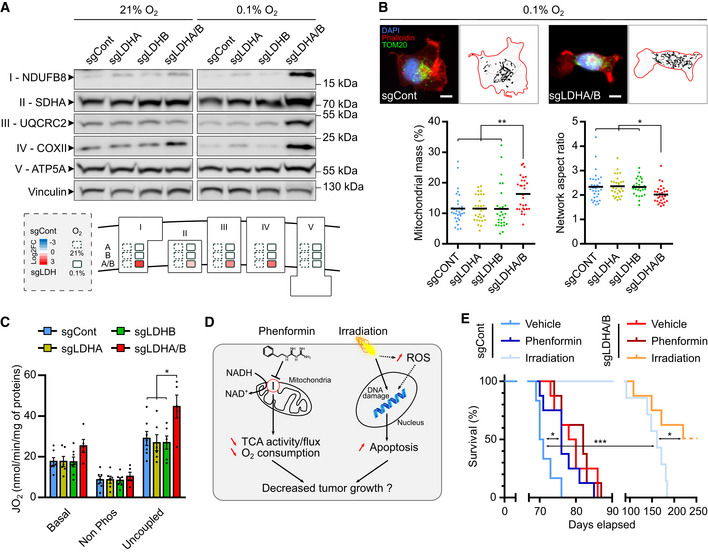
LDHA/B knockout remodels mitochondrial functions and sensitizes tumors to radiation Western blot analysis of mitochondrial respiratory chain subunits from LDH KO P3 cells upon exposure to 21% or 0.1% O_2_. Roman numbers indicate the respiratory complex number. The diagram below represents densitometry quantification of the immunoblots normalized to vinculin and expressed as log_2_ foldchange to control cells in 21% and 0.1% O_2_ (*n* = 3 independent experiments).Epifluorescence (*top*) and quantitative image analysis (*bottom*) of immune‐stained mitochondria (TOM20, green) from LDH KO P3 cells (Phalloidin, red; DAPI, blue) upon exposure to 0.1% O_2_ (*n* = 2 independent experiments, 28–32 cells per group). Data are represented as mean and analyzed using one‐way ANOVA followed by Tukey's multiple comparisons test. Mitochondrial mass: sgCont vs. sgLDHA/B, *P* = 0.005; sgLDHA/sgLDHB vs. sgLDHA/B, *P* = 0.006. Network aspect ratio: sgCont vs. sgLDHA/B, *P* = 0.03; sgLDHA vs. sgLDHA/B, *P* = 0.02; sgLDHB vs. sgLDHA/B, *P* = 0.04. Mitochondrial mass corresponds to the area covered by the mitochondria relative to the entire cell area. Network aspect ratio is the ratio of the major axis to minor axis of the mitochondrial network. Scale bar: 10 μm.LDH KO P3 cell mass‐specific respiration obtained by oxygraphy analysis (*n* = 4 independent experiments). Data are represented as mean ± s.e.m. and analyzed using two‐way ANOVA followed by Tukey's multiple comparisons test: sgCont vs. sgLDHA/B, *P* = 0.015; sgLDHA/sgLDHB vs. sgLDHA/B, *P* = 0.005. For details, see Fig 2H.Schematic representation of phenformin or irradiation effects on tumor cells.Kaplan–Meier survival curves of xenotransplanted mice with LDHA/B KO (red) or control (blue) P3 cells, treated with phenformin or irradiated with 10 Gy (*n* = 6–8 mice per group). Data are analyzed using Log‐rank (Mantel‐Cox) test: sgCont Vehicle vs. sgCont Phenformin, *P* = 0.019; sgCont Vehicle vs. sgCont Irradiation, *P* = 0.0002; sgCont Irradiation vs. sgLDHA/B Irradiation, *P* = 0.024. Source data are available online for this figure.

To corroborate the RNA sequencing results (Figs 4C and D and EV4A), we exposed P3 cells to 21% or 0.1% O_2_ and analyzed the mitochondrial respiratory chain subunit expressions by Western blot. NDUFB8, SDHA, UQCRC2, and COXII protein levels increased at 0.1% O_2_ in double LDHA/B KO cells, but only NDUFB8 and COXII at 21% O_2_ (Fig 5A). Expression of the subunit V ATP5A complex did not change in high or low oxygen conditions (Fig 5A). Only minor changes were observed for single LDHB or LDHA KO cells (Fig 5A). Immunostaining of mitochondria networks revealed an increase in the mitochondrial mass and modifications in network shape, defined as aspect ratio, only in double LDHA/B KO cells (Fig 5B). Under uncoupling conditions, double LDHA/B KO cells possessed higher respiratory capacity than the other cells (Fig 5C). Next, we evaluated cell viability by using 2‐deoxyglucose (2‐DG) and Atpenin A5 (AtpA5) to respectively inhibit glycolysis and OXPHOS via the respiratory complex II (Appendix Fig S7A). At 21% O_2_, 2‐DG induced toxicity in control and LDHA/B KO cells. However, under hypoxia, control cells were more sensitive to 2‐DG than LDHA/B KO cells (Appendix Fig S7B, higher panels). Interestingly, AptA5 induced a higher death rate in double LDHA/B KO cells than in control cells in both oxygen conditions, but these effects appeared moderate (Appendix Fig S7A and B, lower panels). To inhibit redox balance, we chose to use phenformin as an *in vivo* respiratory complex I inhibitor and irradiation to interfere with metabolic adaptations of double LDHA/B KO tumor cells (Fig 5D). Phenformin treatment improved the survival of control tumor‐bearing mice but not of LDHA/B KO tumor‐bearing mice (Fig 5E), but cranial irradiation massively increased global mouse survival, with higher efficiency for mice bearing double LDHA/B KO tumors (Fig 5E).

### The use of anti‐epileptic drug targeting LDH activity efficiently reduces GB development

**Figure 6:**
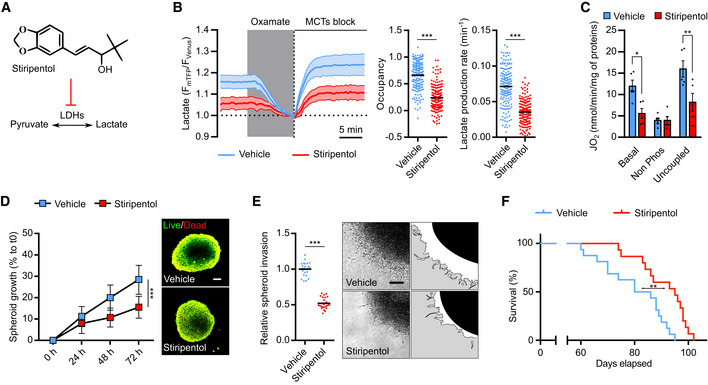
An antiepileptic drug reduces GB growth and invasion Stiripentol, an aromatic allylic alcohol drug that inhibits LDH.Intracellular lactate level was analyzed with a fluorescent biosensor in P3 cells treated with vehicle or 500 μM stiripentol. *Left*, lactate level monitored in basal condition, followed by sequential exposure to 6 mM oxamate and MCTs block (1 μM AR‐C1558585 + 1 mM diclofenac). The response to oxamate and MCTs block was used to determine, *Middle*, the basal lactate level (expressed as biosensor occupancy) and, *Right*, the lactate production rate (*n* = 4, 33–45 cells analyzed per condition). Data are represented as mean ± s.e.m. (*Left*) or as mean (*Middle* and *Right*) and analyzed using unpaired *t*‐test (Occupancy) and Mann–Whitney test (Lactate production). For both analyses, *P* < 0.0001.P3 cell mass‐specific respiration in cells treated with 500 μM stiripentol, obtained by high‐resolution oxygraphy analysis (*n* = 5 independent experiment). Data are represented as mean ± s.d. and analyzed using two‐way ANOVA followed by Sidak's multiple comparisons test: Basal, *P* = 0.013; Non‐Phos, *P* > 0.99; Uncoupled, *P* = 0.003 For details, see Fig 2H.
*Left*: P3 spheroid growth recorded over 72 h during incubation with stiripentol at 0.1% O_2_ (*n* = 3 independent experiments, one experiment including eight spheroids per condition). Data are represented as mean ± s.d., and growth at 72 h is analyzed using unpaired *t*‐test: *P* < 0.0001. *Right*: Viability of spheroids at 72 h, incubated with calcein (green) or ethidium homodimer‐1 (red). Scale bar: 100 μm.
*Left*: P3 spheroid invasion in collagen I gel, incubated 24 h with 500 μM stiripentol at 0.1% O_2_. Invasion rate is expressed as fold change from control (*n* = 3 independent experiments, one experiment including eight spheroids per condition). Data are represented as mean and analyzed using unpaired *t*‐test: *P* < 0.0001. *Right*: Representative images of invasive spheroids for each condition. Scale bar: 100 μm.Kaplan–Meier survival curves of xenotransplanted mice with P3 cells. Mice were treated either with vehicle (blue) or stiripentol at 150 mg/kg (red) (*n* = 15–16 mice per group). Data are analyzed using Log‐rank (Mantel‐Cox) test: *P* = 0.002. Source data are available online for this figure.

By carefully analyzing the literature, we found that the anti‐epileptic drug stiripentol (Fig 6A) was characterized to cross the blood–brain barrier for inhibiting cerebral LDHA/B activity in preclinical therapy studies in mice (Sada *et al*, 2015). We first validated LDH inhibition *in vitro* in P3 cells by intracellular lactate recording with the FRET biosensor *Laconic.* We found that lactate level decreased, and its production was inhibited by 50% after 24 h treatment with 500 μM of stiripentol (Fig 6B). Interestingly, a decrease of lactate production was correlated with a drastic inhibition of basal and uncoupled respiration in stiripentol‐treated P3 cells (Fig 6C). Furthermore, stiripentol significantly reduced P3 spheroid proliferation by 50% at 72 h (Fig 6D) and invasion (Fig 6E). To evaluate stiripentol *in vivo* antitumoral properties, P3 tumor‐bearing mice were intraperitoneally injected with 150 mg/kg of stiripentol. Stiripentol treatment significantly increased survival compared to the control group (median survival was 80 days for control mice and 97 days for stiripentol‐treated mice) (Fig 6F). To conclude, the antiepileptic drug stiripentol is an excellent candidate to reduce GB growth and invasion.

## Discussion

A joint property of many solid cancers, including GBs, is the upregulation of glycolysis, and it has been postulated that blocking glycolysis could be a good strategy to treat GBs. However, by using adapted cell models such as GB stem‐like cells, metabolic plasticity has been described (Garnier *et al*, 2019). A metabolic symbiosis between glycolytic and oxidative areas has also been suggested as one of the main resistance drivers, mainly through a different regional expression of MCT1 and MCT4 (Allen *et al*, 2016). Our study pinpoints lactate as one of the main metabolic drivers, through both LDHA and LDHB activities, supporting tumor development and invasion in GB. Histological analysis of LDHA and LDHB expression demonstrated a clear pattern with LDHA predominantly expressed in the central hypoxic area, in agreement with our previous data (Talasila *et al*, 2017). In contrast, LDHB was expressed in peripheral and invasive tumor areas. Only a few cells were found positive for LDHA in peripheral and invasive regions of the tumor, such as in the corpus callosum. This pattern was confirmed by using spatial transcriptomics data from a recent publication (Ravi *et al*, 2022), in which the hypoxic area is characterized by high LDHA and CA9 expression and the invasive regions by high LDHB expression. These results strongly suggest that GBs are specifically organized to sustain tumor growth and invasion via a metabolic symbiosis based on LDH regional expression and, consequently, on lactate itself. This pattern was also confirmed in the collagen‐embedded invasive spheroid model, where LDHA was mainly found in the hypoxic core of spheroid (Guyon *et al*, 2020) and a few invasive cells. LDHB is exclusively localized at spheroid borders and in invasive cells. This observed differential spatial distribution was reinforced by analyzing a single‐cell database which includes RNAseq data from central and invasive GB regions (Darmanis *et al*, 2017). These results demonstrate that the spatial distribution of LDHA or LDHB echoes the expression of the lactate transporters MCT1 and MCT4 (Allen *et al*, 2016), supporting the hypothesis of a lactate shuttle between tumor cells, as described in the astrocyte‐neuron lactate shuttle (ANLS) (Pellerin *et al*, 1998). We evidenced that lactate—as a single carbon source being retroconverted into pyruvate—can fuel the TCA cycle via stimulation of oxidative phosphorylation, an observation that becomes evident when inhibiting the respiratory chain complex I with rotenone. Our findings also show that lactate alone can regulate tumor invasion over proliferation in cells starved for most carbon sources by quickly fueling the TCA cycle. When glucose is present, lactate and acidification (by HCl) regulate GB invasion (Seliger *et al*, 2013). Acidification of the microenvironment participates in cell invasion by upregulating many factors, such as TGFβ (Seliger *et al*, 2013). Tracing of metabolites with [^13^C_3_] lactate in starved GB stem‐like cells quickly generated labeled pyruvate, which fuels the TCA cycle or is converted into alanine, which was found secreted into the extracellular space. The TCA cycle plays a central role in energy metabolism through acetyl‐CoA oxidation but also in biosynthetic pathways (*e.g*., non‐essential amino acids or fatty acids; Owen *et al*, 2002). We showed that the TCA cycle and the coupled respiratory chain are essential in GB invasion as specific inhibition with rotenone impairs this process. Moreover, exogenous lactate provides an essential and rapid carbon pool for citrate‐mediated fatty acid biosynthesis (Chen *et al*, 2016; Minami *et al*, 2021). Consistent with a previous study (Chen *et al*, 2016), we observed a more minor *m* + 2 isotopologue contribution of α‐ketoglutarate and succinate than citrate.

Single KO of LDHA does not significantly impact the stem‐like GB cell models used in this study, which is different from LDHA KO neuroblastoma cells previously described (Dorneburg *et al*, 2018). When LDHA is absent, compensatory mechanisms are observed in our study, even under low oxygen conditions. KO LDHB cells were more aggressive in *in vitro* and *in vivo* experiments. Since LDHB preferentially mediates the retroconversion of lactate into pyruvate, its deletion led to an accumulation of extracellular lactate and protons, consequently higher invasive potential and a higher production of VEGF, inducing a strong vascular response and lower survival of implanted animals. Indeed, VEGF is one of the strongest inducer of neoangiogenesis and was shown to be activated via lactate and tumor acidosis, thus increasing endothelial cell proliferation (Vegran *et al*, 2011; Sonveaux *et al*, 2012). LDHB KO was previously characterized to be linked to autophagy, and reduction of cell proliferation (Brisson *et al*, 2016), which was not investigated in our study. Double LDHA/B KO decreased both proliferation and invasion and was also linked to the increase in apoptosis, thus improving mouse survival via reduction of the tumor mass. While complementary roles of LDHA and LDHB in brain tumor development have been reported in a neuroblastoma model (Dorneburg *et al*, 2018), double LDHA/B KO in GB stem‐like cells induced a substantial decrease in tumor development via lactate production and consumption impairment. In an elegant publication, Zdralevic and colleagues showed that LDHA or double LDHA/B ablation impaired melanoma and colon adenocarcinoma growth (Ždralević *et al*, 2018). Cerebral tumors rely on low oxygen concentrations, which can partially explain these differences between the models. As lactate is immunosuppressive (Fischer *et al*, 2007), we are currently investigating macrophages/microglia activation in our *in vivo* models, as they represent the main immune cells in GB (Müller *et al*, 2017). Glioblastoma cells, among them GB stem‐like cells, evolve in a different microenvironment than melanoma cells or adenocarcinoma cells, and thus, metabolic regulations during tumor growth may be different.

Accumulation and secretion of incompletely catabolized intermediates could reflect hyperactive metabolism (Meiser *et al*, 2018) or a blockade of essential pathways. The best example is the Warburg effect, where a high glycolytic rate leads to pyruvate excess that cannot be consumed by mitochondria, resulting in significant lactate production and secretion. In addition, GB LDHA/B KO cells lost the ability to maintain the redox balance under anaerobic conditions. Therefore, the double KO globally impacts cellular metabolism. This is associated with a decrease in glucose consumption, accumulation of the majority of intermediates from glycolysis and TCA, and secretion of formate or acetate. One possible explanation for fermentative by‐product overflow is that the pyruvate‐derived acetate is produced to maintain a lower redox potential to compensate for LDH KO (Liu *et al*, 2018). We also observed an increase in the expression of the mitochondrial respiratory complexes in LDHA/B KO cells in hypoxia to counteract the metabolic stress in these extreme conditions. To increase apoptosis in double LDHA/B KO tumors, phenformin was used as an inhibitor of respiratory complex I. However, this treatment failed to increase apoptosis in double LDHA/B KO tumors which is possibly due to the compensation by other respiratory complexes or alternative metabolic pathways such as fatty acid oxidation. Of note, control tumors were found sensitive to phenformin treatment, as suggested by others (Jiang *et al*, 2016). After cranial irradiation, on the contrary, a strong increase in mouse survival was globally observed with a higher efficiency for double LDHA/B KO tumors, validating a combinatory strategy for inhibiting GB growth.

In a recent study, the use of anti‐epileptics as anticancer drugs has been proposed, which could be beneficial for treating GB patients (Jung *et al*, 2019). Therefore, we took advantage of the use of stiripentol, an antiepileptic drug approved by the FDA for the treatment of Dravet Syndrome, because it also potently inhibits both LDHA and LDHB activity (Sada *et al*, 2015). Our results demonstrate that stiripentol inhibits lactate production, but we are the first to describe an inhibitory activity of cell respiration. Both inhibitory activities led to redox imbalance, thus reducing cell proliferation and invasion, and consequently increasing the survival of GB‐bearing mice treated with stiripentol.

Altogether, we conducted a detailed analysis of the metabolic consequences of the deletion or inhibition of LDH enzymes in GB cells and demonstrated that targeting both LDHA and LDHB is required for efficient inhibition of tumor growth by destabilizing metabolic symbiosis between hypoxic and oxygenated areas. These results are of great translational significance because these enzymes may represent attractive candidates for further therapeutic development in GB.

## Materials and Methods

### Ethical issues

All mice were housed under specifically pathogen‐free conditions at the Animalerie Mutualisee de Talence (University of Bordeaux, France) on a 12‐h dark/light cycle with unlimited access to food and water. All experiments were performed with 8‐ to 11‐week‐old male mice and were randomly allocated to the treatment groups. Male RAGγ2C^−/−^ mice were born, housed, and treated in the same animal facility. All animal procedures have been done according to the institutional guidelines and approved by the local ethics committee (agreement number: A5522). The regional ethical committee approved the collection of biopsy tissue at Haukeland University Hospital, Bergen, Norway (REK 013.09), and by Humanitas Hospital (Milan, Italy).

### Antibodies and reagents

The detailed information on primary and secondary antibodies used in this study is listed in Dataset EV1. For immunofluorescence, DAPI was used to stain DNA in blue, and Phalloidin‐Rhodamine was used to stain the cytoskeleton in red. Calcein‐AM and ethidium homodimer‐1 were directly added to cultures in their usual media. Calcein‐AM is converted into green fluorescent calcein by intracellular esterase and is an indicator of cell viability. Injured or dead cells uptake ethidium homodimer‐1 which stained the DNA in red. Spheroids are incubated in these reagents for 45 min at 37°C, and then images were directly taken. Reagents, products, and their corresponding references are listed in Dataset EV1.

### Cell culture

Patient‐derived GB P3 and BL13 cells were generated from patient samples as explained in a previous article (Daubon *et al*, 2019b). Cells were cultured in Neurobasal medium (NBM) supplemented with B27, heparin (100 U/μl), 20 ng/ml basic FGF, and Penicillin–Streptomycin (1,000 U/ml) at 37°C in a 5% CO_2_ incubator. These cell lines underwent mycoplasma testing before their use. Negative mycoplasma contamination status was verified using specific PCR primers. All cell lines were used between passages 10 and 30 in culture to perform experimental procedures. Stable cell lines were generated by infecting lentiviral particles. For the generation of lentiviral particles, HEK293T cells were transfected with the lentiCRISPR v2 vector with single guide RNA (sgRNA), psPAX2 (packaging construct), and pMD2.G (viral envelope). The culture medium was replaced by Opti‐MEM with 20 mM HEPES 6 h after transfection. After 2–3 days of culture, the supernatant was collected and passed through a 0.22 μm syringe filter. The virus‐containing media supplemented with 8 μg/ml polybrene was used to infect P3 and BL13 cells. The infected cells were selected in media with puromycin (0.75 μg/ml) or blasticidin (10 μg/ml) and the LDHA or/and LDHB deletion was assessed by Western blot.

### sgRNA constructs

LDHA and LDHB sgRNAs were designed online (http://crispr.mit.edu/). The target sequences were 5′‐TGCGAATACGCCCACGCGATGGG‐3′ for Control, 5′‐CCGATTCCGTTACCTAATGGGGG‐3′ for LDHA, and 5′‐AAGATCACTGTAGTGGGTGT‐3′ for LDHB. The sgRNAs were then cloned into the lentiCRISPR v2 vector by BsmBI digestion.

### Spheroid experimental assays

Protocols are described in detail in a previous article (Guyon *et al*, 2020). Briefly, P3 and BL13 spheroids of uniform size were prepared from 10^4^ dissociated cells in NBM with 0.4% methylcellulose in a 96‐well round‐bottom plate. The spheroids were used after 3 days of formation. For spheroid growth, each spheroid was washed with PBS and individually replaced in fresh complete neurobasal medium with 0.4% methylcellulose supplemented with different treatments in a 96‐well round bottom plate. For spheroid invasion, spheroids were washed in PBS and individually included in a 96‐well flat‐bottom plate in 100 μl of type I collagen matrix prepared on ice with 1 mg/ml collagen type I and NaOH (initial concentration: 1 M, volume added correspond to 0.023 volume of collagen type I). The matrix was incubated at 37°C for 30 min and then, a complete neurobasal medium with different treatments was added on the matrix.

### Intracranial implantation

For *in vivo* experiments, 5 spheroids of P3 or BL13 knock‐out for LDHA, LDHB, or LDHA/B (only LDHA/B for BL13) and control cell lines were stereotactically implanted into the brain of immunodeficient RAGγ2C^−/−^ mice (*n* = 8 for survival or *n =* 5 for stopping at a given point, 7–9‐week‐old) housed and treated in the animal facility of Bordeaux University (“Animalerie Mutualisée Bordeaux”). The animals were anesthetized with ketamine (1.5 mg/kg) and xylazine (150 μg/kg), and a burr hole was drilled 2.2 mm to the left of the bregma, and the spheroids were implanted into the cerebral cortex at 3 mm depth using a 10 μl Hamilton syringe. An analgesic procedure was applied with the subcutaneous injection of buprenorphine (0.1 mg/kg, once 10 min before and once 12–24 h after implantation). After 4 days, started the bevacizumab injections (10 mg/kg ip), and/or stiripentol (100 mg/kg ip), or phenformin (50 mg/kg ip) injection 3 times a week. The animals were euthanized by cervical dislocation at the end of the experiment, and brains were removed for further histological and immunohistological analyses.

### Histological samples preparation

For the frozen process, mice brains were placed in a cryotube after extraction, directly frozen in liquid nitrogen without fixation, and stored at −80°C. Sections (10 μm) were prepared using a cryostat (CM1900, Microsystems) and mounted on slides. Before immunolabeling process (see below), cryo‐sections were dried at room temperature for 10 min. They were fixed with 4% paraformaldehyde (PFA) for 15 min at room temperature and then washed 3‐times in phosphate‐buffered saline (PBS). For the paraffin process, mice brains or other samples were fixed with 4% PFA for 15 min at room temperature and then washed 3‐times in PBS. Samples were dehydrated serially in 70, 96, and 100% ethanol then toluene, and paraffinized. Sections (10 μm) were prepared using a microtome (Leica) and mounted on slides. Before immunolabeling process (see below), paraffin‐embedded sections were deparaffinized in toluene and hydrated serially in 100, 96, and 70% ethanol and distilled water then for 1 h at 95°C in citrate buffer before the staining procedure.

### Immunolabeling process

Samples were permeabilized with 0.1% Triton X‐100 in PBS for 15 min, quickly washed in PBS, and blocked with 1% bovine serum albumin and 2% fetal donkey serum (FDS) in PBS (blocking buffer) for 1 h. Samples were incubated with primary antibodies in blocking buffer overnight at 4°C, followed by a 3‐time PBS wash and incubation with secondary antibodies in blocking buffer for 1 h, for staining DNA or/and cytoskeleton, samples were treated with DAPI or/and Phalloidin Rhodamine, respectively. Samples were washed 3‐times in PBS and were mounted using Prolong Gold antifade reagent.

### Western blot

Cells were washed twice in PBS and, depending on the analysis, two procedures were used for protein lysis. (1) Cells were lysed in RIPA buffer (10 mM Tris–HCl, pH 7.4, 150 mM NaCl, 0.5% NP‐40, 1%TritonX‐100, 1 mM EDTA) containing proteases and phosphatases inhibitors. Protein concentrations were determined using a bicinchoninic acid assay. Cell lysates were resuspended in Laemmli Buffer (LB ‐ 62.5 mM Tris, 10% glycerol, 2.5% SDS, 5% β‐mercaptoethanol, pH 6.8). (2) Cells were directly lysed in LB. All protein extracts (1 and 2) were separated on an acrylamide gel (10% for LDHA and B, 4–12% precast gels [Invitrogen] for OXPHOS proteins) and transferred to a nitrocellulose membrane. After blocking in blocking buffer for 1 h at room temperature, membranes were incubated with different primary antibodies overnight at 4°C. After 3 washes in TBST (20 mM Tris, 0.5 N NaCl, 0.1% Tween−20, pH = 8) and 1 in TBS (20 mM Tris, 0.5 N NaCl, pH = 8), membranes were incubated with IR‐Dye 680 or 800 labeled secondary antibodies, or secondary antibodies coupled with HRP.

### Image acquisition and analysis

For Western blot analysis, membranes were imaged using Odyssey infra‐red scanner (LI‐COR). The densitometry of proteins was quantified using Image Studio Lite Software with normalization against tubulin or vinculin. Uncropped immunoblot are depicted in Appendix Figs S8–S10. For cell or spheroid experiment assays, images were acquired with an Eclipse Ti Nikon microscope equipped with NIS (Nikon imaging element) software based on ×4, ×10, or ×20 objective lens (Nikon), a Hamamatsu Digital CCD C10600‐10B camera. Images were acquired in brightfield or/and color filters (red/green) at the plane level where the contrast is the most pronounced. Fiji software with different open‐access macros was used for image analysis (Github). Images of the spheroid growth were acquired at 0 h, and every 24 h (total duration dependent on the experiments), growth was quantified as the percent of change of the spheroid area relative to 0 h. Images of the spheroid invasion were acquired at 24 h, and invasion was quantified as the invasive area (total area—core area) normalized to the core area and expressed as a fold change relative to the control group for each independent experiment. For samples from the immunolabeling process, images were acquired with a confocal microscope (Nikon Elipse Ti) or epifluorescence microscope (Nikon). The pipeline analysis of mitochondrial morphology and mass was adapted in Fiji software (Koopman *et al*, 2006). The slide scanner was a Nanozoomer 2.0HT with a fluorescence imaging module (Hamamatsu Photonics France) using objective UPS APO 20X NA 0.75 combined with an additional lens 1.75×, leading to a final magnification of 35×. Virtual slides were acquired with a TDI‐3CCD camera. Fluorescent acquisitions were done with a mercury lamp (LX2000 200W ‐ Hamamatsu Photonics, Massy, France) and the set of filters adapted for DAPI, and/or green fluorescent protein (GFP)/Alexa 488, and/or Alexa 568 and or Alexa 647/Cy5 fluorescence. Images from Appendix Fig S1 were analyzed as previously described analysis (Daubon *et al*, 2019b).

#### TCGA glioblastoma cohort

The TCGA Glioblastoma (GBM) RNAseqV2 normalized data (level 3, log2(*x* + 1) transformed RSEM normalized count, version 2017‐10‐13), the associated clinical data and complementary clinical data from GDC pancan were downloaded from the xenabrowser website datapages (https://xenabrowser.net/datapages/).

For the genes LDHA and LDHB, primary tumor samples from the GBM cohort were split into three groups of equivalent size defined by the level of their expression. Overall survival (in months) was used to estimate survival distributions using the Kaplan–Meier method, and the three distributions were compared using the log‐rank test.

#### Ivy‐GAP cohort

The expression of HIF1A, LDHA, and LDHB was downloaded from the Ivy Glioblastoma Atlas Project (Ivy‐GAP) website (https://glioblastoma.alleninstitute.org/rnaseq/search/index.html) with associated clinical data. Spearman's correlation was computed between HIF1A and LDHA and between HIF1A and LDHA for all samples and for each anatomic structure separately.

#### Spatial transcriptomics analysis

LDHA, LDHB, and CAIX/CA9 expression was analyzed as previously described (Ravi *et al*, 2022) in three different patient samples.

### Sample preparation and transcriptomic profiling

P3 sgControl, sgLDHA, sgLDHB, and sgLDHA/B were cultivated under 0.1% or 21% oxygen for 48 h. Total RNA was extracted from fresh frozen cells with the Qiagen RNeasy Mini Kit according to the manufacturer's protocol. Quality and quantity of RNA was checked using a Fragment Analyzer (Agilent) with the company's Standard Sensitivity RNA Kit (DNF‐471). Libraries were prepared using the TruSeq stranded mRNA Kit (Illumina). All barcoded samples were pooled and sequenced together in 75 nt paired‐end mode with an Illumina NextSeq500 in 2 runs to reach sufficient coverage. Runs were demultiplexed with bcl2fastq (v2.20.0.422, Illumina).

Transcriptomic sequencing was performed in collaboration with Core Unit for Molecular Tumor Diagnostics (CMTD), National Center for Tumor Diseases (NCT), Dresden‐Germany. The R package DESeq2 was used to identify differentially expressed genes using a stringent threshold: absolute value of Log2 Fold Change (LFC) > 1 and *P*‐value adjusted for multiple testing (*P*‐adjust) < 0.01. KEGG pathways enrichment analysis was done with the R package ClusterProfiler (Yu *et al*, 2012). Gene Ontology (GO) terms enrichment analysis was done with the R package BACA with the EASE score set at 0.01. Only GO terms from Biological Pathways level 5 were used for this analysis. GO terms were clustered to get annotation clusters with a similarity of genes > 0.85. Finally, read counts were transformed using variance stabilizing transformations (VST).

#### Bioinformatics analysis

Quality of obtained fastq files was initially checked by FastQC v0.11.4 (Wingett & Andrews, 2018) followed by adapter removal and quality trimming using Trim Galore v0.4.2. Mapping of reads to the human reference genome (GRCh38 Ensembl release 95) was done using STAR v2.5.3a with standard settings (Dobin *et al*, 2013) and duplicates were marked using Picard tools v1.141.

Quality analysis of mapped reads was done using RSeQC v3.0.0 (Wang *et al*, 2012) to analyze read distributions across gene bodies. Raw read counts per gene were determined by counting gene‐specific reads in exons of protein‐coding genes using FeatureCounts v1.5.3 (Liao *et al*, 2014). Finally, a gene expression data matrix was created by removing genes without any reads and lowly expressed genes (< 1 read per million in more than 50% of samples) followed by cyclic loess normalization resulting in normalized log2‐counts per million for 14,111 protein‐coding genes that were measured in each sample.

The R package DESeq2 (Love *et al*, 2014) v1.22.2 was used to identify differentially expressed genes. Enrichment analysis was performed using the ClusterProfiler (Yu *et al*, 2012) R package v3.10.1. Gene Ontology (GO) terms enrichment analysis was visualized using Bubble Chart to Compare Annotations (BACA) using the *P*‐value threshold at 0.01. Only GO terms from Biological Pathways level 5 were used for this analysis. GO terms were clustered to get annotation clusters with a similarity of genes > 0.85. PathView R package v.1.22.3 was used to visualize KEGG metabolic pathways.

### Sample preparation and metabolic profiling

Metabolite extraction was performed using 80% methanol and 0.2% of myristic acid d27 (internal standard). After 5 min of incubation, cells were scraped and collected in a new tube. Following a centrifugation at 20,000 *g* for 10 min at 4°C, the supernatant was transferred to a new vial for MS analysis. Pellet was used for protein quantification.

10 μl of each sample was loaded into a Dionex UltiMate 3000 LC System (Thermo Scientific Bremen, Germany) equipped with a C‐18 column (Acquity UPLC‐HSS T3 1. 8 μm; 2.1 × 150 mm, Waters) coupled to a Q Exactive Orbitrap mass spectrometer (Thermo Scientific) operating in negative ion mode. A step gradient was carried out using solvent A (10 mM TBA and 15 mM acetic acid) and solvent B (100% methanol). The gradient started with 0% of solvent B and 100% of solvent A and remained at 0% B until 2 min post‐injection. A linear gradient to 37% B was carried out until 7 min and increased to 41% until 14 min. Between 14 and 26 min the gradient increased to 100% of B and remained at 100% B for 4 min. At 30 min the gradient returned to 0% B. The chromatography was stopped at 40 min. The flow was kept constant at 250 μl/min at the column and was placed at 25°C throughout the analysis. The MS operated in full scan mode (*m*/*z* range: [70–1,050]) using a spray voltage of 3.2 kV, capillary temperature of 320°C, sheath gas at 10.0, auxiliary gas at 5.0. The AGC target was set at 3e6 using a resolution of 140,000, with a maximum IT fill time of 512 ms. Data collection was performed using the Xcalibur software (Thermo Scientific). The data analyses were performed by integrating the peak areas (El‐Maven—Polly—Elucidata).

Metabolomic profiling was performed in collaboration with MetaboHUB‐MetaToul ([^13^C]_6_ Glucose) and VIB Metabolomics ([^13^C]_6_ Lactate). The Principal Component Analysis (PCA) was done on the total geometric mean abundance of each metabolite in each condition. PCA is realized with the dudi.pca function from the R package « ade4 » and visualized with ggplot. Metabolite abundance between two conditions was analyzed using the non‐parametric Mann–Whitney test with a Benjamini‐Hochberg correction for multiple comparisons. For visualization purposes, comparisons between two conditions may be Log_2_‐transformed.

#### Metabolomics supplemental method: [ ^13^C]_6_ glucose— ^13^C Isotopic profiling

##### Sample preparations

For cells in suspension (fast filtration method), 1 ml of cell culture is dropped on a filter (Sartolon Polyamide 0.2 μm) in order to eliminate cultivation medium. The filter is then rinsed with washing solution, quickly removed from the filtration unit, put on an aluminum foil, and frozen in liquid nitrogen. Every filter is then extracted into a centrifuge tube containing the 5 ml of cold sampling solution (see table below). The centrifuge tubes are then vortexed and placed 1 h at −20°C. After 1 h, the tubes are centrifuged 5 min at 2,000 *g* and the supernatant is put in a new tube for evaporation.

**Table:** 

Types of metabolites	Sampling solution composition	Temperature of extraction	Duration of extraction	Evaporation	Solution of resuspension
Central metabolites	ACN(4)/MeOH(4)/ H_2_Omq (2) 125 mM formic acid	−20°C	1 h minimum	SpeedVac	Water
Intracellular amino acids	ACN(4)/MeOH(4)/ H_2_Omq (2) 125 mM formic acid	−20°C	1 h minimum	SpeedVac	Water
Coenzyme A	ACN(4)/MeOH(4)/ H_2_Omq (2) 125 mM formic acid	‐20°C	1 h minimum	SpeedVac	2% methanol, 98% water, 25 mM formic acid, pH adjusted at 3.5

For cell supernatants, the sampling procedure is based on the separation of the cells from the medium thanks to the combination of filtration and centrifugation. This gives access only to the exometabolome content for metabolomics or fluxomics studies. The supernatant can be stored at −80°C before shipment and analysis evaporated. Then samples are prepared either for NMR or MS analysis manually or automatically using a robotic station.

To control the quality of the analysis, blank samples are done for each type of sample (culture medium, conditions, treatment…). These blank samples are obtained from a “cell‐free” culture made in parallel and sampled in the same way and time as the culture with cells.

##### Nuclear Magnetic Resonance (NMR) profiling

The acquisition of 1D 1H NMRAvance II 800 MHz equipped with a 5 mm CQPCI Z‐Gradient cryoprobe. Following parameters were used for the acquisition: Pulse program: zgpr30; Pulse angle 30°; Time Domaine (TD) 64 k; Number of dummy scan: 4; Number of scan: 32; Acquisition time: 2.04 s; Pulse P1 length: 7.70 μs; Pulse P1 power: −12.39 dB; Pulse P9 power: 43.79 dB; Acquisition temperature: 280°K. Raw data obtained after acquisition are FID. A Fourier transform was applied for each spectrum with a specific smoothing (efp with LB = 0.3 and SI = 128 K). Phase and baseline correction was also performed using automatic tools from TopSpin 3.5 software before the automatic integration of specific signals belonging to exo‐metabolites present in the samples. Absolute quantification of metabolites of interest was performed using the internal standard TSP‐d4 as reference. The quality of the analysis is based on the good resolution of the spectrum: width at half height for TSP‐d4 signal < 2.5 Hz.

##### Liquid chromatography/Mass Spectrometry analysis

Central metabolites were separated on an ionic chromatography column IonPac AS11 (250 × 2 mm i.d.; Dionex, CA, USA). Solvent used was KOH at a flow rate of 350 μl/min. Solvent was varied as follows: 0 min: 2%, 2 min: 2%, 10 min: 5%, 16 min: 35%, 20 min: 100% and 24 min: 100%. The column was then equilibrated for 6 min at the initial conditions before the next sample was analyzed. The volume of injection was 15 μl. High‐resolution experiments were performed with an ICS5000+, ion chromatography system (Dionex, CA, USA) coupled to an LTQ Orbitrap Velos mass spectrometer (Thermo Fisher Scientific, Waltham, MA, USA) equipped with a heated electrospray ionization probe. MS analyses were performed in negative FTMS mode at a resolution of 60,000 (at 400 *m*/*z*) in full‐scan mode, with the following source parameters: the capillary temperature was 350°C, the source heater temperature, 300°C, the sheath gas flow rate, 50 a.u. (arbitrary unit), the auxiliary gas flow rate, 5 a.u., the S‐Lens RF level, 60%, and the source voltage, 3.5 kV.

Amino acids were separated on a PFP column (150 × 2.1 mm i.d., particle size 5 μm; Supelco Bellefonte, PEN, USA). Solvent A was 0.1% formic acid in H20 and solvent B was 0.1% formic acid in acetonitrile at a flow rate of 250 μl/min. Solvent B was varied as follows: 0 min: 2%, 2 min: 2%, 10 min: 5%, 16 min: 35%, 20 min: 100% and 24 min: 100%. The column was then equilibrated for 6 min at the initial conditions before the next sample was analyzed. The volume of injection was 5 μl. High‐resolution experiments were performed with a Vanquish HPLC system coupled to an Orbitrap Qexactive+ mass spectrometer (Thermo Fisher Scientific, Waltham, MA, USA) equipped with a heated electrospray ionization probe. MS analyses were performed in positive FTMS mode at a resolution of 70,000 (at 400 *m*/*z*) in full‐scan mode, with the following source parameters: the capillary temperature was 320°C, the source heater temperature, 300°C, the sheath gas flow rate, 40 a.u. (arbitrary unit), the auxiliary gas flow rate, 10 a.u., the S‐Lens RF level, 40%, and the source voltage, 5 kV.

All the metabolites were determined by extracting the exact mass with a tolerance of 5 ppm. For central metabolites isotopic profile analysis and amino acids isotopic profile analysis, their concentrations have to be included in the dynamic range of the method, respectively. This range was determined during the method validation with the PA‐PT sample and corresponds to the total area of the cluster/number of isotopologues with a bias of < 5%.

##### Data quality

Filters and supernatants were received on 03.04.19 and analyzed on 10.04.19 (MS) and 11.04.19 (NMR). All the acceptability criteria were satisfied, and values meet MetaToul's acceptance as shown in the table below.

**Table:** 

		Acceptability criteria
MS calibration ≤ 7 days	Day of injection (11.04.19)	Passed
Bias on Pascal Triangle sample ≤ 5%	PEP: −1.4% max	Passed
	FruBP: −2.5% max	Passed
	ATP: 2.4% max	Passed

##### Data extraction and quality

The data extraction of the raw mass spec data files yielded information that could be loaded into a relational database. Peaks were identified using IsoCor peak integration software (Millard *et al*, 2012).

##### Normalization

Data correction was performed to correct variation resulting from the difference of cell number into each condition. For abundance interpretation, sample P3 LDHA/B at 24 and 48 h was concentrated 2 times.

### Metabolomic and transcriptomic representation

Metabolome and transcriptome were independently analyzed (see above). Due to the difficulty in examining detailed metabolic networks using several cell lines and conditions, two representations are proposed. The first one, called metabologram (Hakimi *et al*, 2016), has the ability to visualize data at pathway level relative to the second one, which is the classical detailed network map of central carbon. In order to avoid redundancy of data, the metabologram showed all the isotope abundances in metabolites and the network map showed only the [^13^C]‐labeled abundance in metabolite in order to trace the pathway of [^13^C]‐initial metabolite. For each pathway and after the stringent threshold based on log_2_ FC and *P*‐value (*P* < 0.05), transcripts are selected according to the KEGG pathway database.

### Intracellular lactate recording

Cultured P3 spheroids were gently dissociated and seeded on 18 mm glass coverslips treated with Matrigel. Constructs coding for the lactate‐sensitive FRET biosensor Laconic (San Martín *et al*, 2013) have been described previously and are available through Addgene (Plasmid #44238). Adenoviral vectors encoding the FRET biosensor were custom‐made by Vector Biolabs. The lactate‐sensitive biosensor Laconic was expressed by exposing cells to 1 × 10^6^ PFU of adenoviral particles (serotype 5) overnight. After 48–72 h post‐infection, cells were imaged on wide‐field mode with an inverted LEICA DMI6000B microscope (Leica Microsystems, Germany) equipped with a motorized stage Scan IM (Märzhäuser, Germany), a 40× oil‐immersion objective (NA 1.25), a excitation system Lumencor spectra 7 (Lumencor, US) and a Coolsnap HQ2 CDD camera (Photometrics, US). Cells were superfused with an imaging solution consisting of (in mM): 10 HEPES, 112 NaCl, 24 NaHCO3, 3 KCl, 1.25 MgCl_2_, 1.25 CaCl_2_, 10 glucose and bubbled with air/5% CO_2_ at 37°C with a constant flow of 3 ml/min. For fluorescent ratio measurements, cells expressing Laconic were excited at 430 nm for 0.05–0.1 s, and emission was collected with band‐pass filters mounted on a motorized filter wheel, at 465–485 nm for mTFP and 542–556 nm for Venus, with image acquisition every 10 s. The ratio between mTFP and Venus was computed and is proportional to the intracellular lactate level. To quantify differences in lactate level, biosensor occupancy was computed as a proxy of intracellular lactate level with the following equation: Occupancy = (R‐Rmin)/(Rmax‐Rmin), in which R: basal mTFP/Venus ratio (before any drug treatment), Rmin: steady‐state mTFP/Venus ratio induced by oxamate (6 mM), Rmax: steady‐state mTFP/Venus ratio induced by MCTs block (1 μM AR‐C155858 and 1 mM diclofenac). Lactate production rate was computed by fitting a linear rate to the first minutes of lactate accumulation during MCTs block. Fluorescence ratios were normalized to Rmin before quantifications.

### LDHA immunoprecipitation and enzymatic activity assay

LDHA was immunoprecipitated, and the enzymatic activity was measured as previously described (Ji *et al*, 2017) with some modifications.

#### Immunoprecipitation

In brief, cells were incubated for 4 h in a buffer containing 0.3% Nonidet P‐40, 150 mM NaCl, 50 mM Tris–HCl (pH 7.5), and inhibitors of proteases and phosphatases. Lysates, obtained by centrifugation at 13,000 *g* for 10 min, were pre‐cleared with protein A/G agarose beads (16% of sample volume) for 1 h. After a short centrifugation, pellets were discarded and the protein content of the supernatants was measured by Bradford assay. Samples, diluted to obtain equal protein concentration, were incubated overnight with anti‐LDHA antibodies (LDHA sc‐137,243, Santa Cruz Biotechnology) at the final concentration of 9 ng of Ab/μg of proteins. Then, protein A/G agarose beads (16% of sample volume) were added to the lysates, which were incubated for further 4 h. After a short centrifugation, the pellets containing LDHA proteins coupled to the agarose beads were washed once and resuspended in Tris–HCl 0.2 M pH 7.3. All the centrifugation steps were performed at 4°C, incubations were carried out at 4°C with gentle shaking. When indicated, cells were incubated at 0.1% O_2_ for 48 h.

#### Enzymatic assay

LDHA‐immunoprecipitated proteins were added to the reaction buffer (0.2 M Tris–HCl pH 7.3, 0.05% BSA, 10 mM MgCl_2_, 2 mM pyruvate, 0.5 mM NADH) and the enzymatic activity was determined by measuring NADH oxidation (reduction in absorbance) at 340 nm at 25°C using a CLARIOstar (BMG Labtech).

### LDH and LDHB activities

In the LDH assay (ab102526), LDH reduces NAD to NADH, which then interacts with a specific probe to produce a color (OD max = 450 nm) based on whole lysates (LDHA + LDHB isozymes) while LDHB activity is measured on LDHB enzyme which was immune‐captured within the wells of the microplate (ab140361) and determined by following the production of NADH catalyzed by the enzyme (LDHB isozymes, alone). Recommendations from the manufacturer were followed for the optimal procedure.

### Elisa VEGF

Supernatants were collected from all cell lines and a VEGF Human ELISA Kit (Thermo Fisher), and procedure was followed based on manufacturer instructions.

### Statistical analysis

No statistical methods were used to predetermine the sample size. The experiments were not randomized, and investigators were not blinded to allocation during experiments and outcome assessment. GraphPad Prism software and RStudio were used for analyses. Equality of variances was made with a *F*‐test for two groups and a Brown‐Forsythe test for at least three groups. According to homoscedasticity, comparisons of two groups were made by an unpaired two‐tailed *t*‐test or a Mann–Whitney test. Comparisons of at least three groups were performed using a one‐way ANOVA followed by a Dunnett's or Tukey's multiple comparisons test or Kruskal–Wallis test followed by a Dunn's multiple comparisons test. A two‐way ANOVA test was used to compare two or more two groups with two variables or more than two variables (*e.g*., 21% and 0.1% O_2_) or repeated measures at different times followed by a Tukey's multiple comparisons test. Survival analyses were made using log‐rank Mantel‐Cox test. All the tests used were indicated in the figure legends. *P* values < 0.05 were considered statistically significant.

**The paper explained**

**Problem**

Lactate is a central metabolite in brain function but also in tumor development. Glioblastomas are very heterogeneous and severe brain tumors with minimal treatment for patients. Yet, a better understanding of how brain tumor cells adapt to oxygen constraints via metabolic rewiring is crucial.

**Results**

Using multiple models such as immunostainings on 3D invasive spheroids or patient‐derived tumors and spatial transcriptomics data on patient material, we described a regional expression of lactate dehydrogenase (LDH) A and B in glioblastoma, suggesting a metabolic symbiosis between glycolytic and oxidative cells through lactate transfer. Indeed, LDHA is more expressed in hypoxic areas, and a few invasive cells, and LDHB is expressed in oxygenated/vascularized and invasive regions. We then showed that lactate, massively produced in hypoxic areas, fuels TCA cycle to sustain the invasion and proliferation of glucose‐starved cells. Only double LDHA/B KO led to a drastic decrease in tumor development and invasion. However, metabolic adaptation through respiratory chain remodeling and activity happened in double LDHA/B KO cells, even under low oxygen concentration. Thus, cranial radiation of the LDHA/B KO tumors further improved mouse survival. Finally, we repurposed an antiepileptic drug, stiripentol, to directly target LDHA and LDHB activity, which led to decreased cell respiration, proliferation, and invasion, and further increased survival in intracranially implanted mice.

**Impact**

Metabolic symbiosis in tumors is a central phenomenon which could be a good therapeutic target to efficiently treat patients with glioblastoma. Finding drugs crossing the blood–brain barrier and having specific inhibition activity on major metabolic enzymes, such as LDHs, led to a massive decrease in glioblastoma development in mouse models. Potential clinical studies can be based on these promising results.

## Author contributions

**Joris Guyon:** Data curation; formal analysis; investigation; visualization; methodology; writing—review and editing. **Ignacio Fernandez‐Moncada:** Conceptualization; formal analysis; validation; investigation; methodology. **Claire M Larrieu:** Data curation; formal analysis; investigation. **Cyrielle L Bouchez:** Data curation; investigation. **Antonio C Pagano Zottola:** Data curation; formal analysis; investigation; methodology. **Johanna Galvis:** Software; investigation; methodology. **Tiffanie Chouleur:** Investigation. **Audrey Burban:** Investigation. **Kevin Joseph:** Resources; validation; methodology. **Vidhya M Ravi:** Resources. **Heidi Espedal:** Investigation. **Gro Vatne Røsland:** Investigation. **Boutaina Daher:** Investigation. **Aurelien Barre:** Formal analysis; methodology. **Benjamin Dartigues:** Formal analysis; methodology. **Slim Karkar:** Methodology. **Justine Rudewicz:** Formal analysis; methodology. **Irati Romero‐Garmendia:** Investigation. **Barbara Klink:** Resources. **Konrad Grützmann:** Resources; methodology. **Marie‐Alix Derieppe:** Investigation. **Thibaut Molinié:** Investigation. **Nina Obad:** Investigation. **Céline LEON:** Investigation. **Giorgio Seano:** Writing—review and editing. **Hrvoje Miletic:** Writing—review and editing. **Dieter Henrik Heiland:** Resources. **Giovanni Marsicano:** Supervision; writing—original draft. **Macha Nikolski:** Supervision; methodology; writing—original draft; writing—review and editing. **Rolf Bjerkvig:** Resources; supervision; funding acquisition; writing—original draft. **Andreas Bikfalvi:** Supervision; funding acquisition; writing—original draft; writing—review and editing. **Thomas Daubon:** Conceptualization; data curation; formal analysis; supervision; funding acquisition; validation; investigation; methodology; writing—original draft; project administration; writing—review and editing.

## Disclosure and competing interests statement

The authors declare that they have no conflict of interest.

## Supporting information



Appendix

Expanded View Figures PDF

Dataset EV1

Source Data for Expanded View

Source Data for Figure 1

Source Data for Figure 2

Source Data for Figure 3

Source Data for Figure 4

Source Data for Figure 5

Source Data for Figure 6

PDF+

## Data Availability

Transcriptomics data for this study have been deposited in the European Nucleotide Archive (ENA): accession number PRJEB45718.
