# Supplementary material for: Lactate dehydrogenases promote glioblastoma growth and invasion via a metabolic symbiosis
Source: EMBO Mol Med. 2022 Oct 24;14(12):e15343. doi: 10.15252/emmm.202115343 (PMC9728051; doi:10.15252/emmm.202115343)
Supplement: Supplementary file 1 — Appendix [file EMMM-14-e15343-s004.pdf]

Appendix

Lactate dehydrogenases promote glioblastoma growth and invasion *via* a metabolic symbiosis

Joris Guyon[1], Ignacio Fernandez-Moncada[2,19], Claire M. Larrieu[3,19], Cyrielle L. Bouchez[3,19], Antonio C. PaganoZottola[3,19], Johanna Galvis [3,4], Tiffanie Chouleur[1], Audrey Burban[3], Kevin Joseph[5,6,7,8,9], Vidhya M. Ravi[5,6,7,8,9,10], Heidi Espedal[11], Gro Vatne Rosland[3], Boutaina Daher[3], Aurélien Barre[4], Benjamin Dartigues[4], Slim Karkar[4], Justine Rudewicz[4], Irati Romero-Garmendia[3], Barbara Klink[12,13,14], Konrad Grützmann[14], Marie-Alix Derieppe[15], Thibaut Molinié[3], Nina Obad[11], Céline Léon[1], Giorgio Seano[16], Hrvoje Miletic[11,17], Dieter Henrik Heiland[5,6,7,8,18] , Giovanni Marsicano[2], Macha Nikolski[3,4], Rolf Bjerkvig[11], Andreas Bikfalvi[1,*], and Thomas Daubon[1,3,#,*].

[1]University Bordeaux, INSERM U1312, University Bordeaux, Pessac, France ;
[2]University Bordeaux, INSERM, U1215 Neurocentre Magendie, Bordeaux, France ;
[3]University Bordeaux, CNRS, IBGC, UMR 5095, Bordeaux, France ;
[4]Bordeaux Bioinformatic Center CBiB, University of Bordeaux, France ;
[5]Microenvironment and Immunology Research Laboratory, Medical Center - University of Freiburg, Freiburg, Germany ;
[6]Department of Neurosurgery, Medical Center - University of Freiburg, Freiburg, Germany ;
[7]Faculty of Medicine, University of Freiburg, Freiburg, Germany ;
[8]Translational NeuroOncology Research Group, Medical Center - University of Freiburg, Freiburg, Germany;
[9]Center of Advanced Surgical Tissue Analysis (CAST), University of Freiburg, Freiburg, Germany ;
[10]Freiburg Institute for Advanced Studies (FRIAS), University of Freiburg, Freiburg, Germany ;
[11]NorLux Neuro-Oncology, Department of Biomedicine, University of Bergen, Norway ;
[12]Department of Oncology, Luxembourg Institute of Health, 1526, Luxembourg, Luxembourg ;
[13]German Cancer Consortium (DKTK), 01307, Dresden, Germany ;
[14]Core Unit for Molecular Tumor Diagnostics (CMTD), National Center for Tumor Diseases (NCT), 01307, Dresden, Germany ;
[15]Animal Facility, University Bordeaux, 33615, Pessac, France ;
[16]Institut Curie, INSERM U1021, CNRS UMR3347, Tumor Microenvironment Lab, University Paris-Saclay, 91400 Orsay, France ;
[17]Department of Pathology, Haukeland University Hospital, Bergen, Norway ;
[18]German Cancer Consortium (DKTK), partner site Freiburg, Freiburg, Germany ;
[19]Authors contributed equally to this work.

***Corresponding authors and co-principles investigators**: thomas.daubon@u-bordeaux.fr and andreas.bilfalvi@u-bordeaux.fr

[#] Lead author: thomas.daubon@u-bordeaux.fr

Keywords: Glioblastoma, lactate dehydrogenases, energy metabolism, invasion, antiepileptic drug.

Short title: lactate dehydrogenases in GB development

Table of Content:

Appendix Figure S1: Colocalization analysis of LDHA/LDHB and CAIX/LDHA staining

Appendix Figure S2: Expression of LDHA and LDHB in publicly available databases

Appendix Figure S3: Metabolic tracing using [$^{13}C_3$]lactate with malate-aspartate shuttle inhibitor cycloserine (Extended data Figure 2I)

Appendix Figure S4: Detailed metabolograms

Appendix Figure S5: Bioinformatics analysis based on RNAseq data from P3 sgControl adaptations to hypoxia or from basal differences between P3 sgControl and P3 sgLDHA/B cells

Appendix Figure S6: Nucleotide tracing using [$^{13}C_6$]glucose

Appendix Figure S7: Viability assay of P3 cells using glycolysis and ETC2 inhibitor

Appendix Figure S1

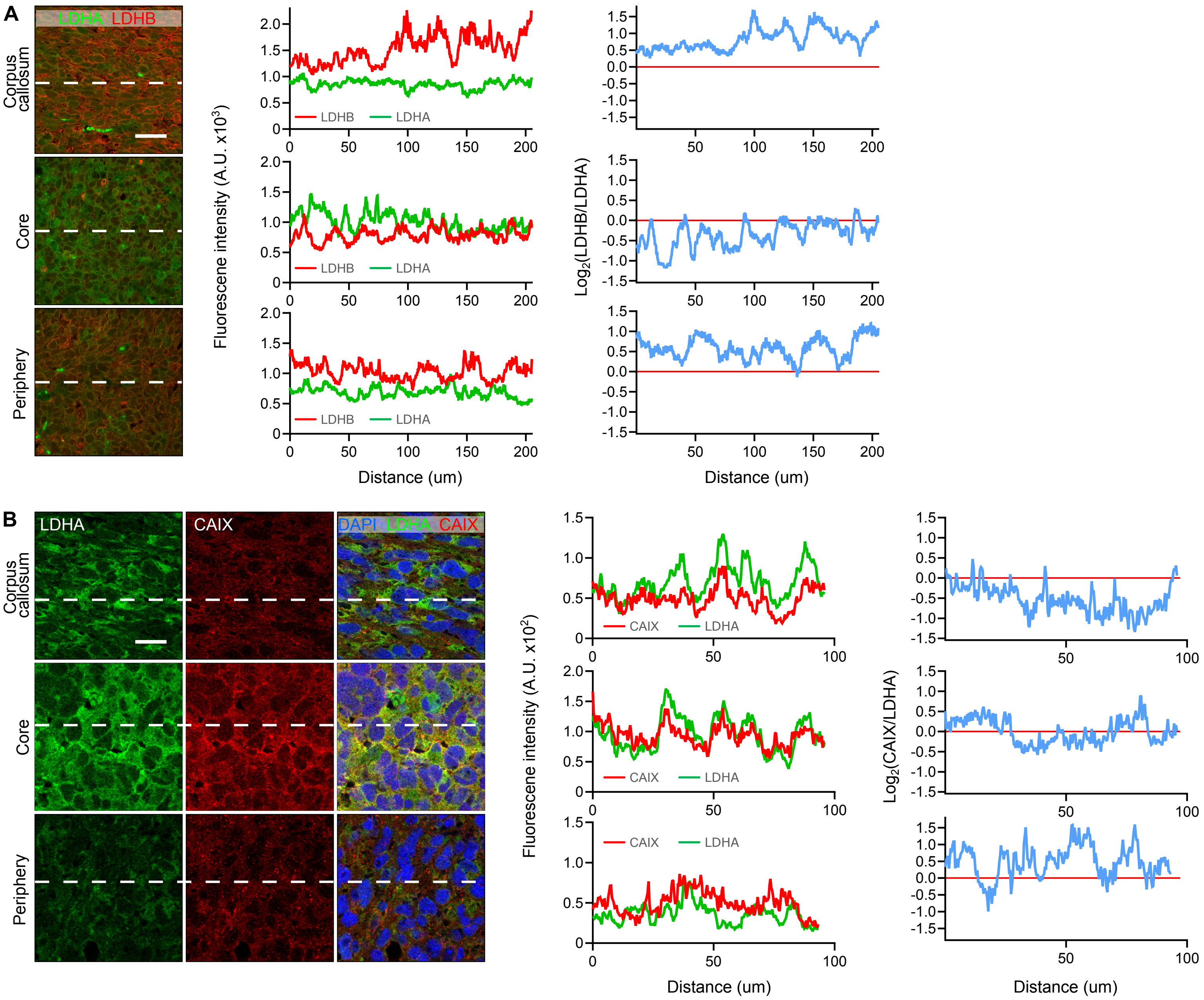

Appendix Figure S1: Colocalization analysis of LDHA/LDHB and CAIX/LDHA staining (Extended data Figure 1)

A. Same images as in Figure 1 were used in the colocalization analysis, by extracting pixel values along the dashed lines. Scale bar: 50 µm. The graphs (middle panels) represent intensity values of LDHB (red) and LDHA (green) staining in P3 tumor in the 3 different areas (corpus callosum, core, and periphery), using Fiji software. In the right panels, a Log_2FoldChance = 0 means that LDHA and LDHB are similarly expressed, and when different from 0, the expression is stronger for one of the two proteins.

B. Immunostaining of CAIX (red) and LDHA (green) were performed from P3 tumor and images were taken from the core, the periphery and invasive (corpus callosum) area. DAPI was used for nuclear staining (blue). Scale bar: 50 µm. The graphs represent intensity values of CA9 (red) and LDHA (green) staining in P3 tumor in the 3 different areas (corpus callosum, core, and periphery), using Fiji software. In the right panels, a Log_2FoldChance = 0 means that LDHA and CA9 are similarly expressed, and when different from 0, the expression is stronger for one of the two proteins.

Appendix Figure S2

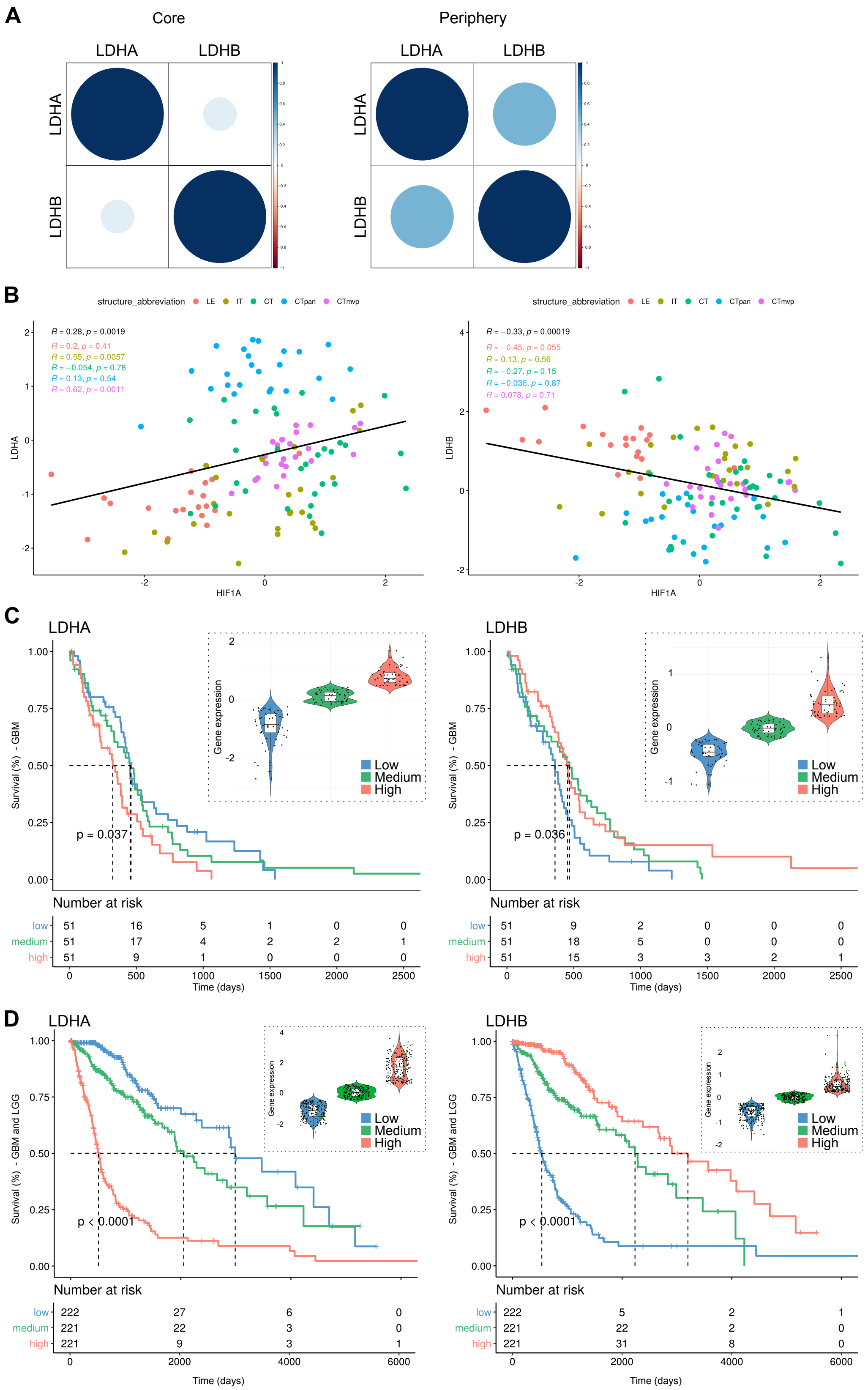

Appendix Figure S2: Expression of LDHA and LDHB in publicly available databases (Extended data Figure 1)

A. Correlation between gene expression of LDHA and LDHB and cell localization based on single cell RNA sequencing data extracted from Darmanis et al.(Darmanis et al., 2017) (1010 tumor cells and 62 periphery cells).

B. *LDHA* and *LDHB* gene expression relative to HIF1A gene expression according to their anatomical origin (data extracted from Ivy Glioblastoma Atlas Project). LE, Leading Edge; IT, Infiltrating Tumor; CT, Cellular Tumor; CTpan, Cellular Tumor pseudopalisading cells around necrosis; CTmvp, Cellular Tumor microvascular proliferation.

C, D. Survival analysis based on *LDHA* (*left*) or *LDHB* (*right*) gene expression level in glioblastoma (**C**) and on low grade gliomas (**D**). Data were extracted from TCGA.

Appendix Figure S3

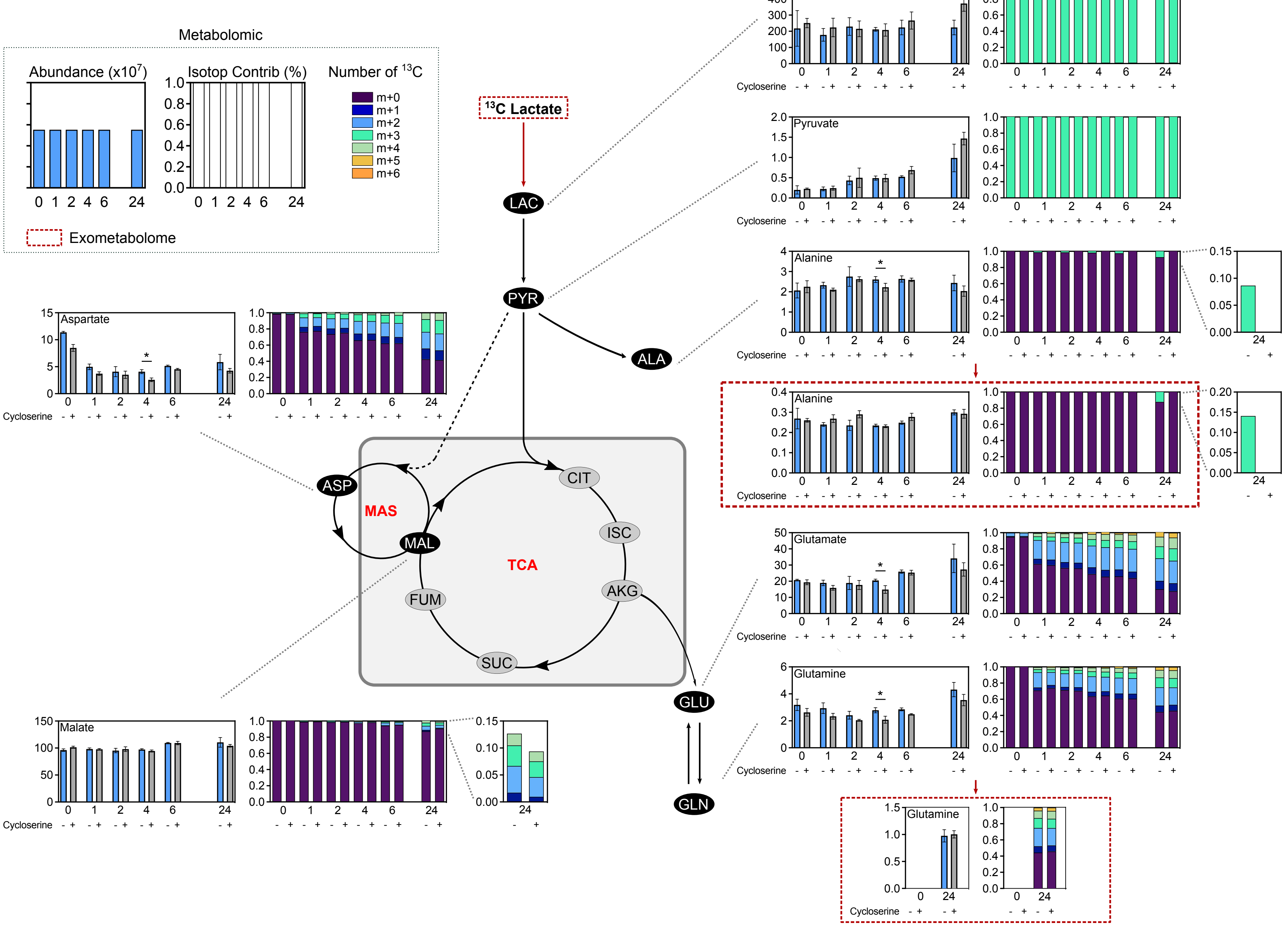

Appendix Figure S3: Metabolic tracing using [$^{13}C_3$]lactate with malate-aspartate shuttle inhibitor cycloserine (Extended data Figure 2I)

P3 cells, pretreated during 24 h with 50 µM of cycloserine, were infused during 0, 1, 2, 4, 6 and 24 h with [$^{13}C_3$]lactate at a concentration of 5 mM. Metabolites from cell extracts (endometabolome) or cell medium (exometabolome, red lines) measured by liquid chromatography-mass spectrometry (*n* = 3 independent cell dishes for each condition). Metabolite abundance of some intermediates of metabolic pathway of interest, data are represented as mean ± s.d. Quantification of the [$^{13}C_3$]lactate carbon incorporation into intermediates of the carbon metabolism (isotopologue contribution), data are represented as mean. m+0 stands for the fraction of metabolite without ^{13}Carbon and m+n (n > 0) stands for fraction of metabolite with n ^{13}Carbon. The sum of (m+0, m+1,…, m+10,…) equals to 1.

Appendix Figure S4

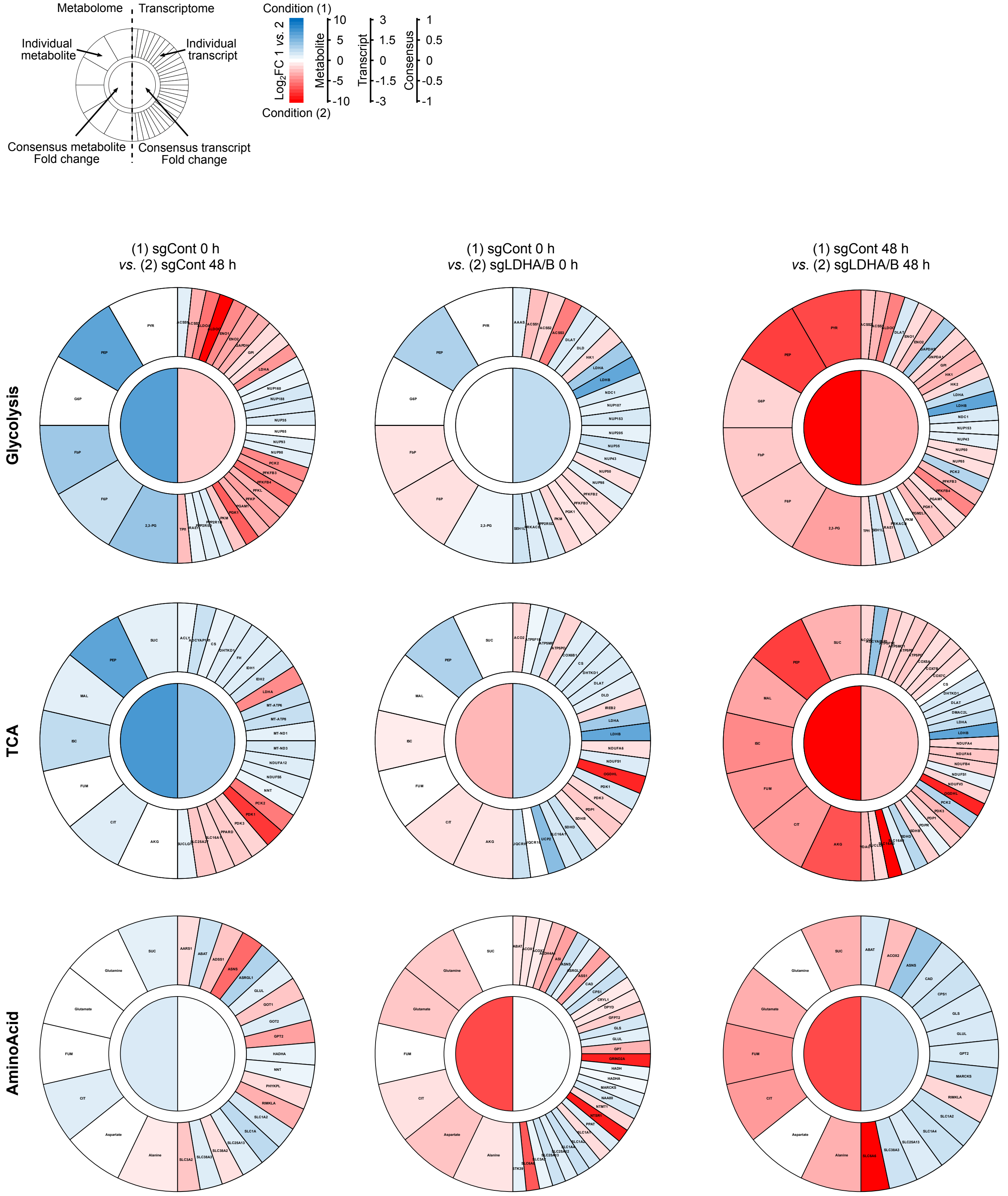

Appendix Figure S4: Detailed metabolograms (Extended data Figure 4C)

Circular metabologram illustrating metabolic and transcriptomic differences in metabolite pathways between LDH KO P3 cells. The metabologram is divided in two parts, the left corresponds to metabolomic analysis and the right to the transcriptomic analysis. The outer circle corresponds to the $\log_2$ fold change for each metabolite (*left*) and transcripts (*right*). The central circle displays the average fold change of all analytes. Metabolites and gene names were added into these metabolograms.

Appendix Figure S5

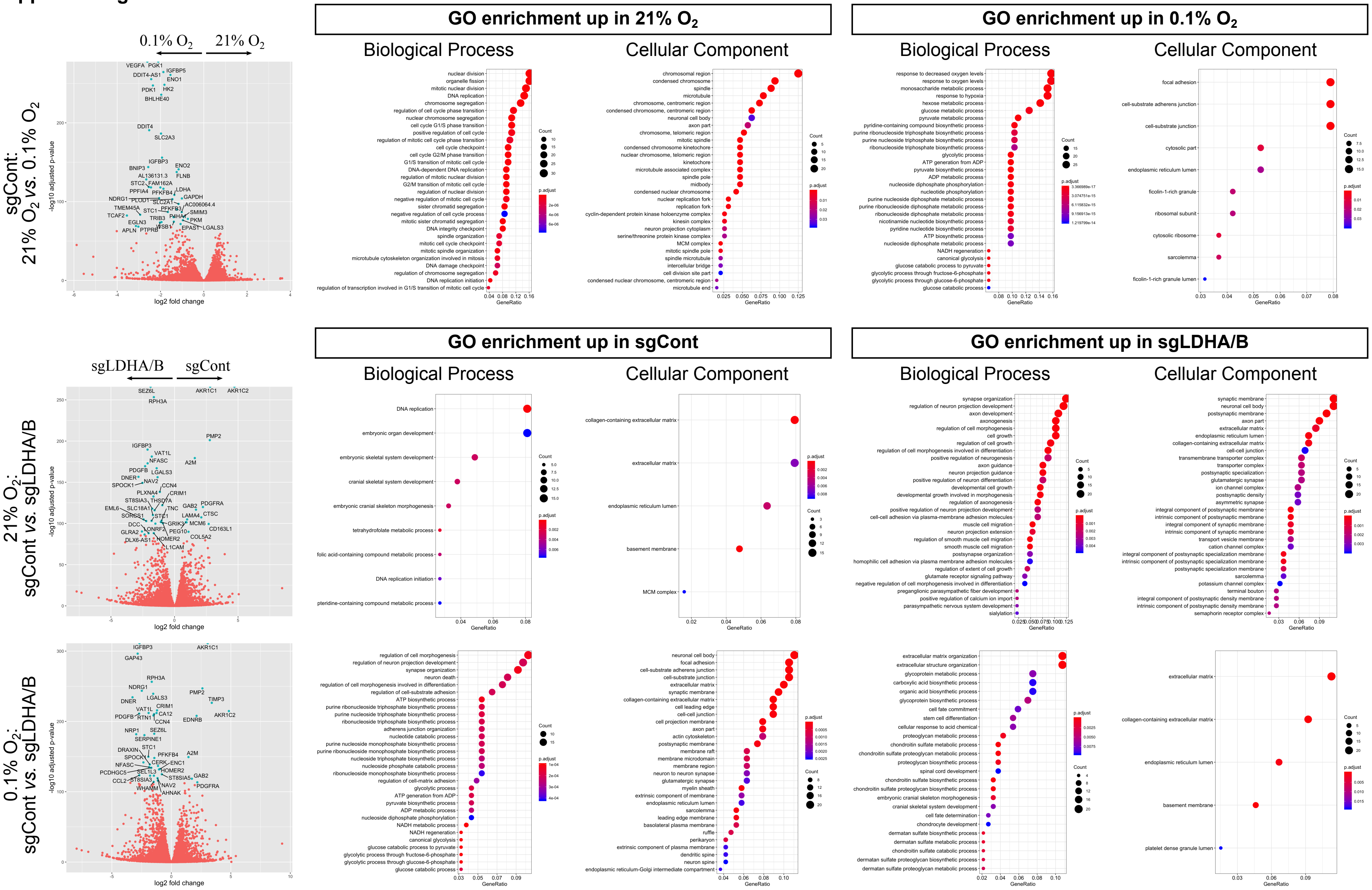

Appendix Figure S5: Bioinformatics analysis based on RNAseq data from P3 sgControl adaptations to hypoxia or from basal differences between P3 sgControl and P3 sgLDHA/B cells (Extented data Figure 4)

Left: Volcano plots for visualizing gene expression in described comparisons. *Right:* Enrichment analysis using Gene Ontology with filtered terms “biological process” and “cellular component”.

Appendix Figure S6

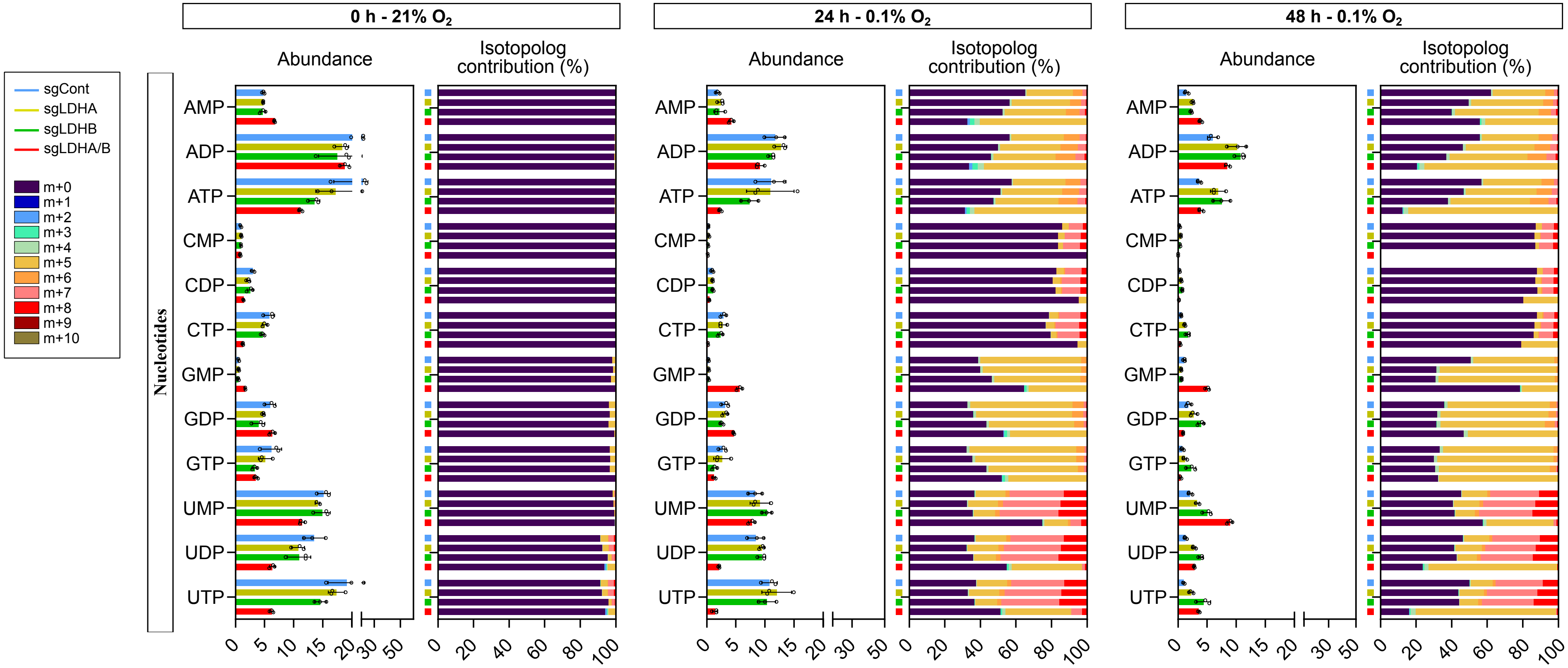

Appendix Figure S6: Nucleotide tracing using [$^{13}C_6$]glucose (Extented data Figure 4)

P3 sgControl, sgLDHA, sgLDHB and sgLDHA/B were infused during 0, 24 and 48 h at 0.1% O2 with [13C_6]glucose. Nucleotides from cell extracts were measured by gas chromatography-mass spectrometry (*n* = 3 independent cell dishes for each condition). Abundance and isotopolog contribution of all nucleotide isotopes from glucose metabolism are shown, data are represented as mean ± s.d. and as mean, respectively. m+0 stands for the fraction of metabolite without ^{13}Carbon and m+n (n > 0) stands for fraction of metabolite with n ^{13}Carbon. For example, m+5 correspond to a metabolite with 5 labeled ^{13}Carbon. The sum of (m+0, m+1, …, m+10) equals to 1.

Appendix Figure S7

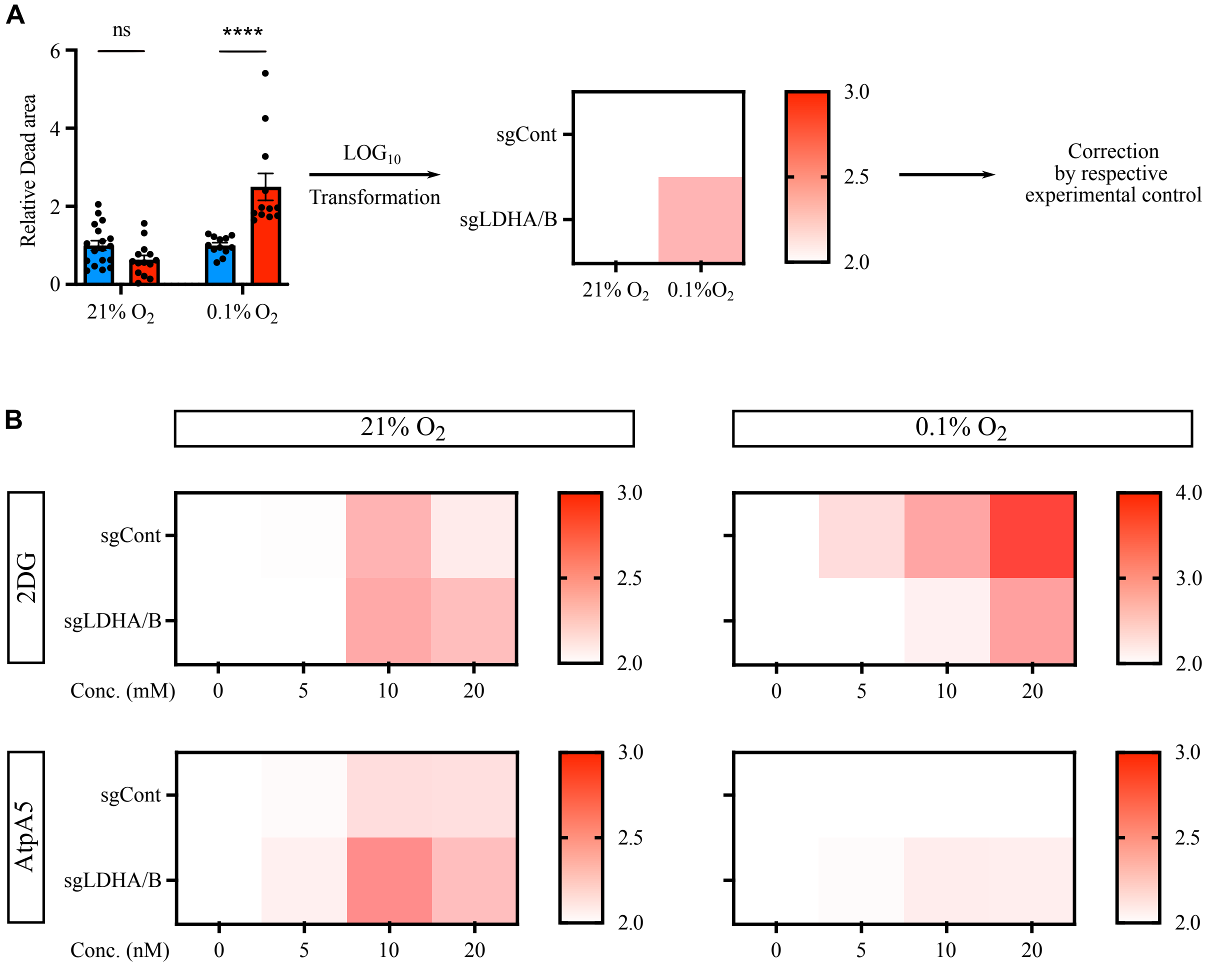

Appendix Figure S7: Viability assay of P3 cells using glycolysis and ETC2 inhibitor

A. P3 sgCont and sgLDHA/B spheroids were incubated at 21% or 0.1% O_2 and viability assay was assessed after 3 days of incubation. Data were generated from spheroid viability assays (calcein/ethidium homodimer to respectively detect live/dead cells) by normalizing values by their own internal control. Values were then log-transformed and represented in heatmaps. No note, basal cell death of double LDHA/B KO spheroid was considered to be higher as already shown in Supplementary Figure 6E.

B. P3 sgCont and sgLDHA/B spheroids were treated by multiple concentration of 2-DG (glycolysis inhibitor, from 5 to 20 mM) and Atpenin-A5 (AtpA5, ETC2 inhibitor, from 5 to 20 nM) and incubated at 21% or 0.1% O_2. Viability assay was assessed after 3 days of incubation by incubating with calcein and ethidium homodimer.
