# Supplementary material for: Lactate dehydrogenases promote glioblastoma growth and invasion via a metabolic symbiosis
Source: EMBO Mol Med. 2022 Oct 24;14(12):e15343. doi: 10.15252/emmm.202115343 (PMC9728051; doi:10.15252/emmm.202115343)
Supplement: Supplementary file 2 — Expanded View Figures PDF [file EMMM-14-e15343-s009.pdf]

Expanded View Figures

Figure EV1. Independent stem-like cell line BL13 confirms results obtained in P3 stem-like cells (Extended data Fig 2A–H).

A Western blot analysis of LDHA and LDHB from BL13 cells upon exposure to 21% or 0.1% O_2 during 6, 24, 48, and 72 h. The graphs represent densitometry quantification of the immunoblots normalized to tubulin ($n = 4$ independent experiments). Data are represented as mean ± s.d. and analyzed using one-Way ANOVA following Dunnett's multiple comparisons test. LDHA: O_2 21% vs. O_2 0.1% 6 h, $P = 0.81$; O_2 21% vs. O_2 0.1% 24 h, $P = 0.01$; O_2 21% vs. O_2 0.1% 48 h, $P = 0.0002$; O_2 21% vs. O_2 0.1% 72 h, $P = 0.0004$. LDHB: O_2 21% vs. O_2 0.1% 6 h, $P = 0.35$; O_2 21% vs. O_2 0.1% 24 h, $P = 0.34$; O_2 21% vs. O_2 0.1% 48 h, $P = 0.44$; O_2 21% vs. O_2 0.1% 72 h, $P > 0.99$.

B Lactate secretion of BL13 cells exposed to 21% or 0.1% O_2 measured by bioluminescent assay using a pro-luciferin reductase substrate converted to luciferin in the presence of NADH ($n = 3$ independent experiments). Data are represented as mean ± s.d. and analyzed using unpaired *t*-test: $P = 0.0009$.

C Enzymatic assays for the activity of LDH enzymes in BL13 cells ($n = 3$ independent experiments). Data are represented as mean ± s.d. and analyzed using unpaired *t*-test: $P = 0.008$.

D Enzymatic assays for the activity of immune-captured LDHB in BL13 cells ($n = 3$ independent experiments). Data are represented as mean ± s.d. and analyzed using unpaired *t*-test: $P = 0.81$.

E P3 spheroid cytotoxicity assay was recorded over 24 h when incubated with or without lactic acid at different concentrations (1, 10, 20, 30, 40, 50, and 100 mM). Area of spheroids was measured at 0 and 24 h. Growth is represented as a percentage of the spheroid area when compared to time 0, and viability is estimated with live/dead fluorescence ratio at 24 h and represented as a fitted curve ($n = 3$ independent experiments, one experiment including 8–10 spheroids per condition). Data are represented as mean ± s.d. and growth at 72 h are analyzed using Kruskal–Wallis test followed by Dunn's multiple comparison test: Control vs. LA 1 mM, $P > 0.99$; Control vs. LA 10 mM, $P = 0.27$; Control vs. LA 20 mM, $P > 0.99$; Control vs. LA 30 mM, $P < 0.0001$; Control vs. LA 40 mM, $P < 0.0001$; Control vs. LA 50 mM, $P < 0.0001$; Control vs. LA 100 mM, $P = 0.77$. Images of representative spheroids in each condition (in green, calcein; in red, ethidium homodimer-1). Scale bar: 250 μm.

F Principal component analysis of morphologic data on P3 cells incubated 7 days with or without lactate (20 mM). Cell number and morphology were measured at 0 h, 24 h, and 7 days ($n = 3$ independent experiments, one experiment including 2–3 independent cell dishes). Images of representative adherent cells in each condition (in green, GFP; in red, nuclear Tomato). Scale bar: 40 μm. The graphs represent quantification of the cell number and elongated cells (Aspect ratio > 2:5; $n = 3$). Data are represented as mean ± s.d. and analyzed using two-way ANOVA followed by Sidak's multiple comparisons test. Number of cell: 24 h, $P = 0.43$; 7 days, $P < 0.0001$. Elongated cell: 24 h, $P < 0.0001$; 7 days, $P < 0.0001$.

G P3 spheroid invasion in collagen I gel incubated 24 h at 21% O_2 and treated with 20 mM pyruvate or 1.5 mM HCl. Invasion rate is expressed as a fold change of the control ($n = 3$ independent experiments, one experiment including 6–8 spheroids per condition). Data are represented as mean ± s.d. and analyzed using Kruskal–Wallis test followed by Dunn's multiple comparison test: Control vs. Pyruvate, $P < 0.0001$; Control vs. HCL, $P = 0.09$; Pyruvate vs. HCL, $P = 0.0002$.

© 2022 The Authors

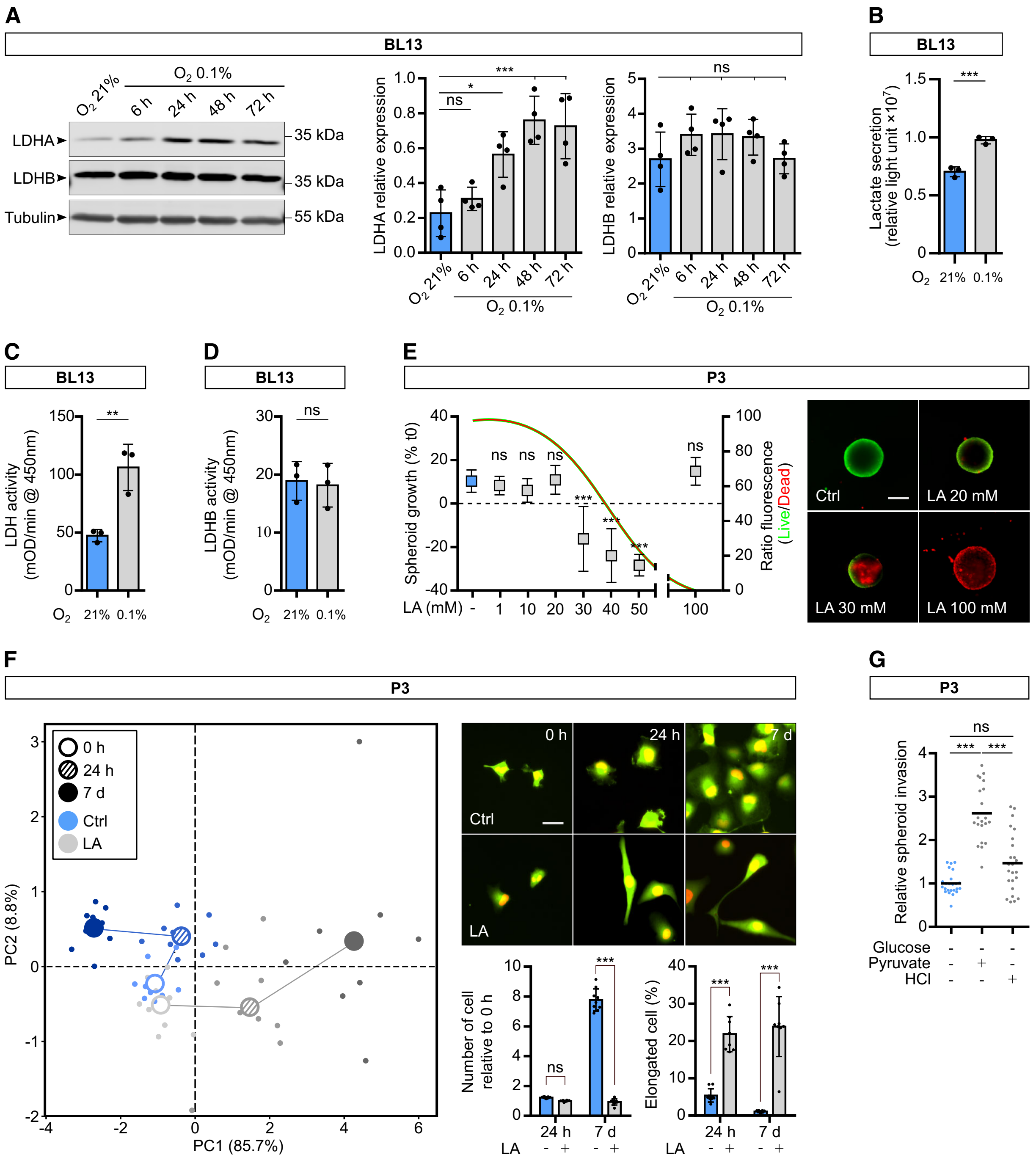

Figure EV1.

 © 2022 The Authors

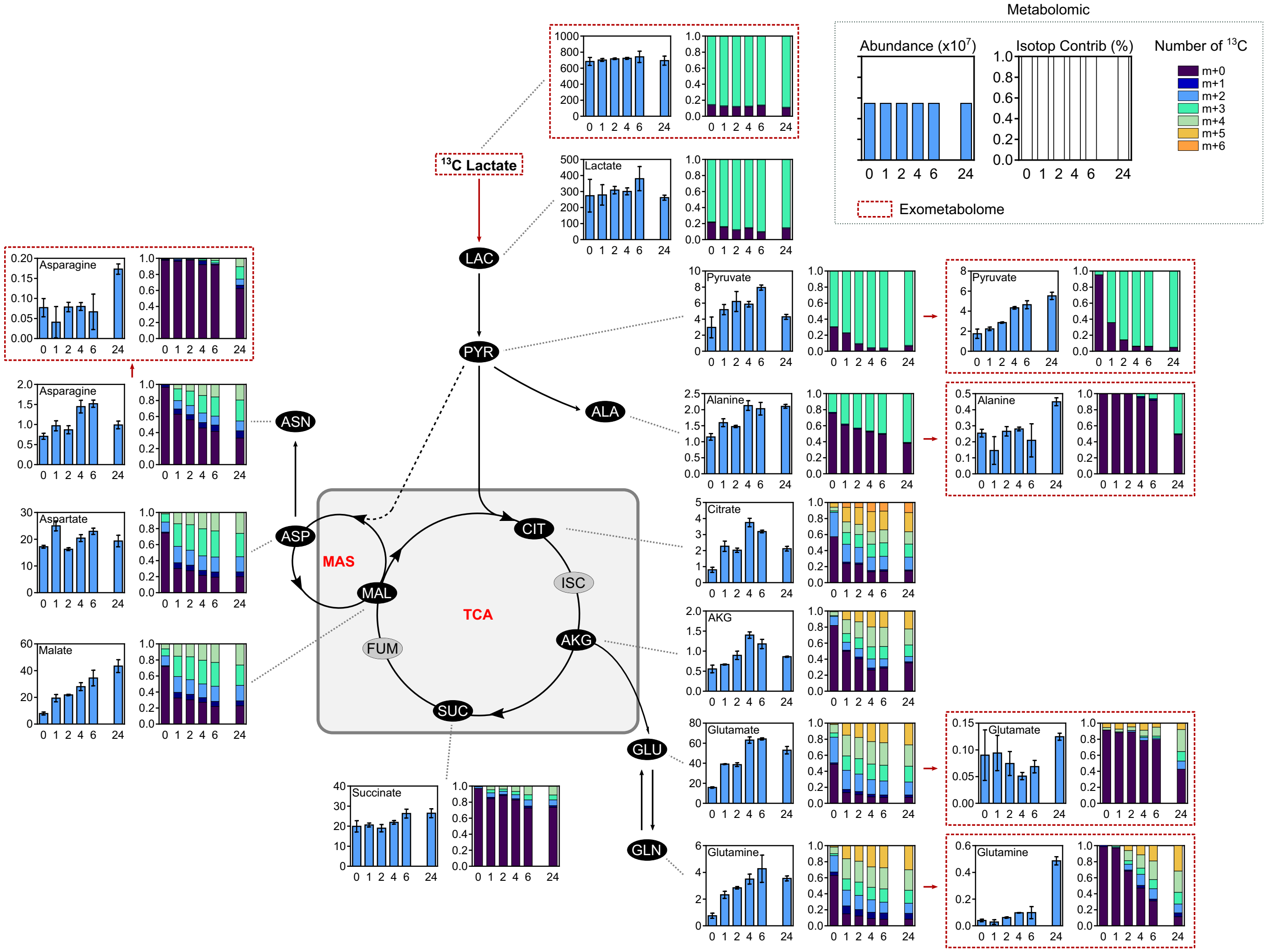

Figure EV2. Metabolic tracing using [$^{13}C_3$] lactate (Extended data Fig 2I).

P3 cells were infused during 0, 1, 2, 4, 6, and 24 h with [$^{13}C_3$] lactate at a concentration of 5 mM. Metabolites from cell extracts (endometabolome) or cell medium (exometabolome, red lines) measured by liquid chromatography–mass spectrometry (n = 3 independent cell dishes for each time point). Metabolite abundance of some intermediates of metabolic pathway of interest, data are represented as mean ± s.d. Quantification of the [$^{13}C_3$] lactate carbon incorporation into intermediates of the carbon metabolism (isotopologue contribution), data are represented as mean. $m + 0$ stands for the fraction of metabolite without ^{13}Carbon and $m + n$ ($n > 0$) stands for fraction of metabolite with n ^{13}Carbon. The sum of ($m + 0$, $m + 1$, . . ., $m + 10$, . . .) equals to 1.

© 2022 The Authors

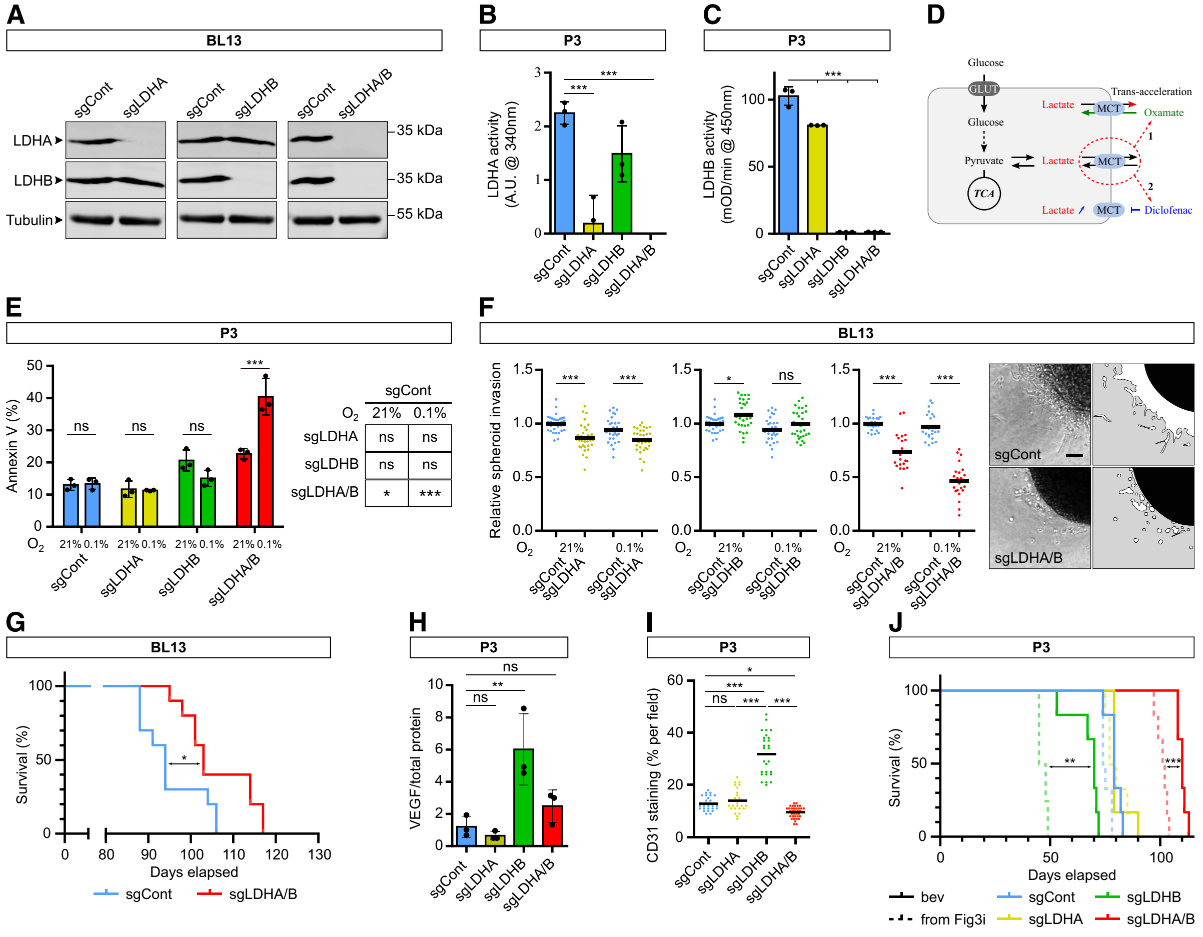

O2	sgCont 21%	sgCont 0.1%
sgLDHA	ns	ns
sgLDHB	ns	ns
sgLDHA/B	*	***

Figure EV3. LDHA/B KO in BL13 cells and bevacizumab treatment in P3 tumors (Extended data Fig 3).

A Western blot analysis of LDHA and LDHB from BL13 cells knockout by CRISPR-Cas9 lentiviral vectors against LDHA, LDHB, or both, and upon exposure to 21% O_2.

B Enzymatic assays for the activity of LDHA in P3 cells ($n = 3$ independent experiments). Data are represented as mean ± s.d. and analyzed using one-way ANOVA followed by Dunnett's multiple comparison test: sgCont vs. sgLDHA, $P = 0.0005$; sgCont vs. sgLDHB, $P = 0.1$; sgCont vs. sgLDHA/B, $P < 0.0001$.

C Enzymatic assays for the activity of immune-captured LDHB in P3 cells ($n = 3$ independent experiments). Data are represented as mean ± s.d. and analyzed using one-way ANOVA followed by Dunnett's multiple comparison test: sgCont vs. sgLDHA, $P = 0.0002$; sgCont vs. sgLDHB, $P < 0.0001$; sgCont vs. sgLDHA/B, $P < 0.0001$.

D Schematic representation of the intracellular lactate level monitoring with a fluorescent biosensor. The presence of the lactate changes the conformation of the biosensor and fluorescence emission. Known as an accelerated-exchange transport (trans-acceleration), oxamate was used to quickly release the lactate out of the cells for the determination of the lactate basal level. Then, diclofenac was used to block the lactate transporter for the quantification of the lactate production rate.

E Cells incubated during 48 h at 21% or 0.1% O_2, labeled with Annexin-V FITC, and analyzed by cytometry ($n = 3$ independent experiments). Data are represented as mean ± s.d. and analyzed using two-way ANOVA followed by Tukey's multiple comparison test: sgCont 21% vs. sgCont 0.1%, $P > 0.99$; sgLDHA 21% vs. sgLDHA 0.1%, $P > 0.99$; sgLDHB 21% vs. sgLDHB 0.1%, $P = 0.3$, sgLDHA/B 21% vs. sgLDHA/B 0.1%, $P < 0.0001$. Table of statistical comparisons of Annexin-V signal in sgLDHA, sgLDHB and sgLDHA/B cells with respective control (either 21 or 0.1% O_2): sgCont 21% vs. sgLDHA 21%, $P > 0.99$; sgCont 0.1% vs. sgLDHA 0.1%, $P = 0.98$; sgCont 21% vs. sgLDHB 21%, $P = 0.07$; sgCont 0.1% vs. sgLDHB 0.1%, $P > 0.99$; sgCont 21% vs. sgLDHA/B 21%, $P = 0.01$; sgCont 0.1% vs. sgLDHA/B 0.1%, $P < 0.0001$.

F BL13 spheroid invasion in collagen I gel incubated 24 h at 21 or 0.1% O_2. Invasion rate is expressed as a fold change of the control ($n = 4$ independent experiments, one experiment including 7–8 spheroids per condition). Data are represented as mean and analyzed using two-way ANOVA test followed by Tukey's multiple comparison test: sgCont 21% vs. sgLDHA 21%, $P < 0.0001$; sgCont 0.1% vs. sgLDHA 0.1%, $P = 0.001$; sgCont 21% vs. sgLDHB 21%, $P = 0.03$; sgCont 0.1% vs. sgLDHB 0.1%, $P = 0.29$; sgCont 21% vs. sgLDHA/B 21%, $P < 0.0001$; sgCont 0.1% vs. sgLDHA/B 0.1%, $P < 0.0001$. Images of representative invasive sgControl or sgLDHA/B spheroids. Scale bar: 100 μm.

G Kaplan–Meier survival curves of xenotransplanted mice with BL13 cells KO for LDHA/B (red) or control (blue) ($n = 10$ mice per group). Data are analyzed using log-rank (Mantel-Cox) test: $P = 0.02$.

H Supernatants were collected from each cell line and analyzed by using ELISA to detect VEGF ($n = 3$ independent experiments). Data are represented as mean ± s.d. and analyzed using one-way ANOVA followed by Dunnett's multiple comparison test: sgCont vs. sgLDHA, $P = 0.91$; sgCont vs. sgLDHB, $P = 0.004$; sgCont vs. sgLDHA/B, $P = 0.5$.

I Tumor blood vessels were stained with anti-CD31 antibodies and CD31 staining area was calculated over tumor area (related to Figs 3F and G). Data are represented as mean and analyzed using one-way ANOVA followed by Tukey's multiple comparison test: sgCont vs. sgLDHA, $P = 0.81$; sgCont vs. sgLDHB, $P < 0.0001$; sgCont vs. sgLDHA/B, $P = 0.034$; sgLDHA vs. sgLDHB, $P < 0.0001$; sgLDHB vs. sgLDHA/B, < 0.0001.

J Kaplan–Meier survival curves complement of Fig 3H where xenotransplanted mice with LDHA/B KO P3 spheroids were treated by bevacizumab ($n = 8$ mice per group). Data are analyzed using log-rank (Mantel-Cox) test: sgLDHB vs. sgLDHB + bevacizumab, $P = 0.001$; sgLDHA/B vs. sgLDHA/B + bevacizumab, $P = 0.0005$.

© 2022 The Authors

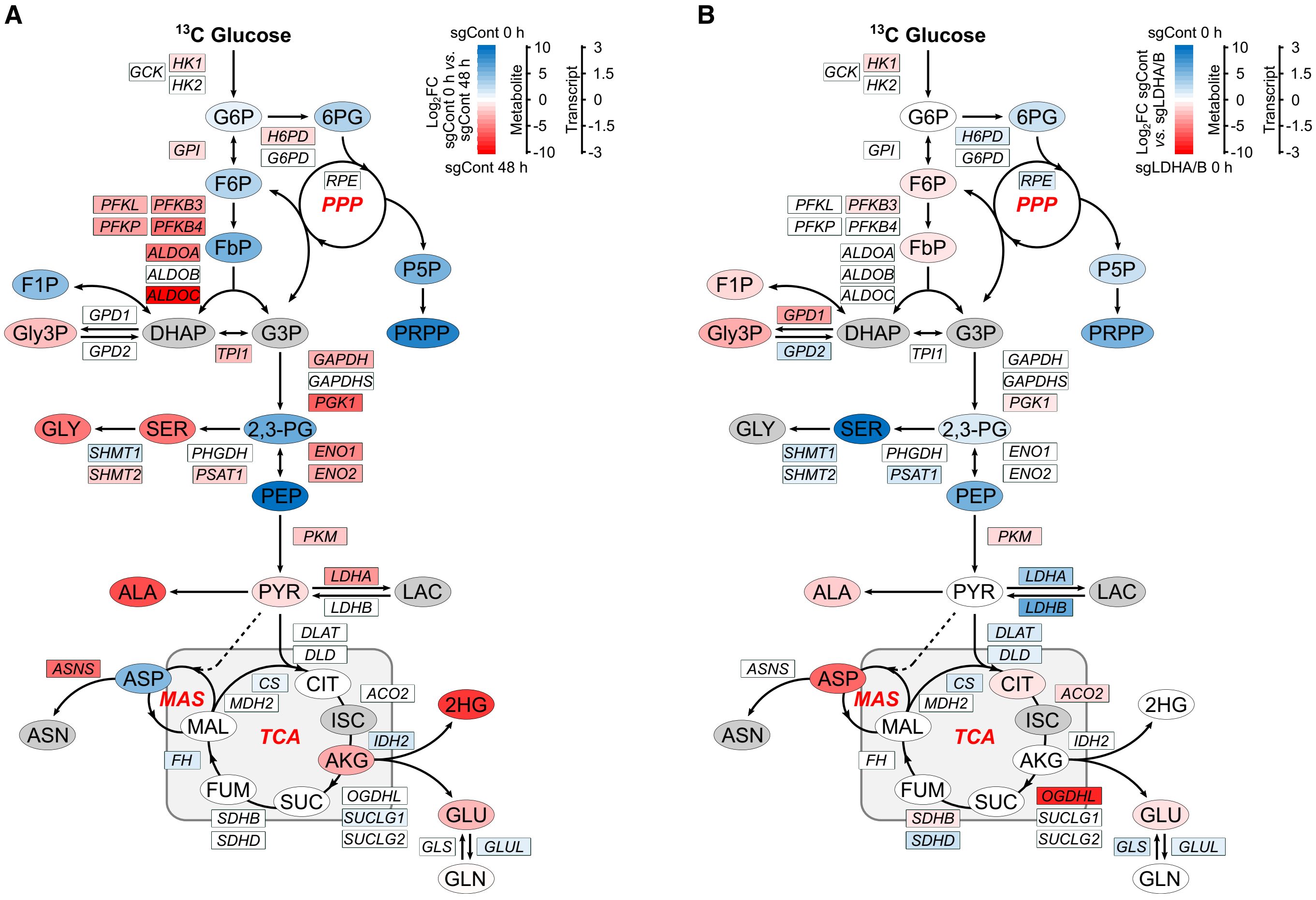

Figure EV4. P3 sgControl RNAseq/metabolomic adaptation profiles to hypoxia and basal differences between P3 sgControl and P3 sgLDHA/B cells (Extended data Fig 4).

Metabolic changes of central 13-labeled-carbon metabolism when knock-out P3 cells are infused with $[^{13}C_6]$ glucose. Metabolites are labeled with colored oval and enzyme transcripts with colored square, colors correspond to the $\log_2$ fold changes between:

A sgCont 0 h and sgCont 48 h at 0.1% O_2 (blue, increase in sgCont 0 h; red, increase in sgCont 48 h; gray, not measured or not computable).

B sgCont 0 h and sgLDHA/B 0 h (blue, increase in sgCont 0 h; red, increase in sgLDHA/B 0 h; gray, not measured or not computable). For details, see also Figs 4 and EV5.

Figure EV5. Metabolic tracing using $[^{13}C_6]$ glucose (Extended data Fig 4).

P3 sgControl, sgLDHA, sgLDHB, and sgLDHA/B were infused during 0, 24, and 48 h at 0.1% O_2 with $[^{13}C_6]$ glucose. Metabolites from cell extracts (endometabolome) were measured by liquid chromatography–mass spectrometry ($n = 3$ independent cell dishes for each condition and time point) and transcripts by RNA sequencing. Abundance and isotopologue contribution of all metabolites from glucose metabolism are shown. For abundance, data are represented as mean ± s.d., and for isotopologue contribution, data are represented as mean. $m + 0$ stands for the fraction of metabolite without ^{13}Carbon and $m + n$ ($n > 0$) stands for fraction of metabolite with n ^{13}Carbon. For example, $m + 5$ corresponds to a metabolite with 5 labeled ^{13}Carbon. The sum of ($m + 0$, $m + 1$, . . ., $m + 10$, . . .) equals 1. For details, see also Figs 4 and EV4.

© 2022 The Authors

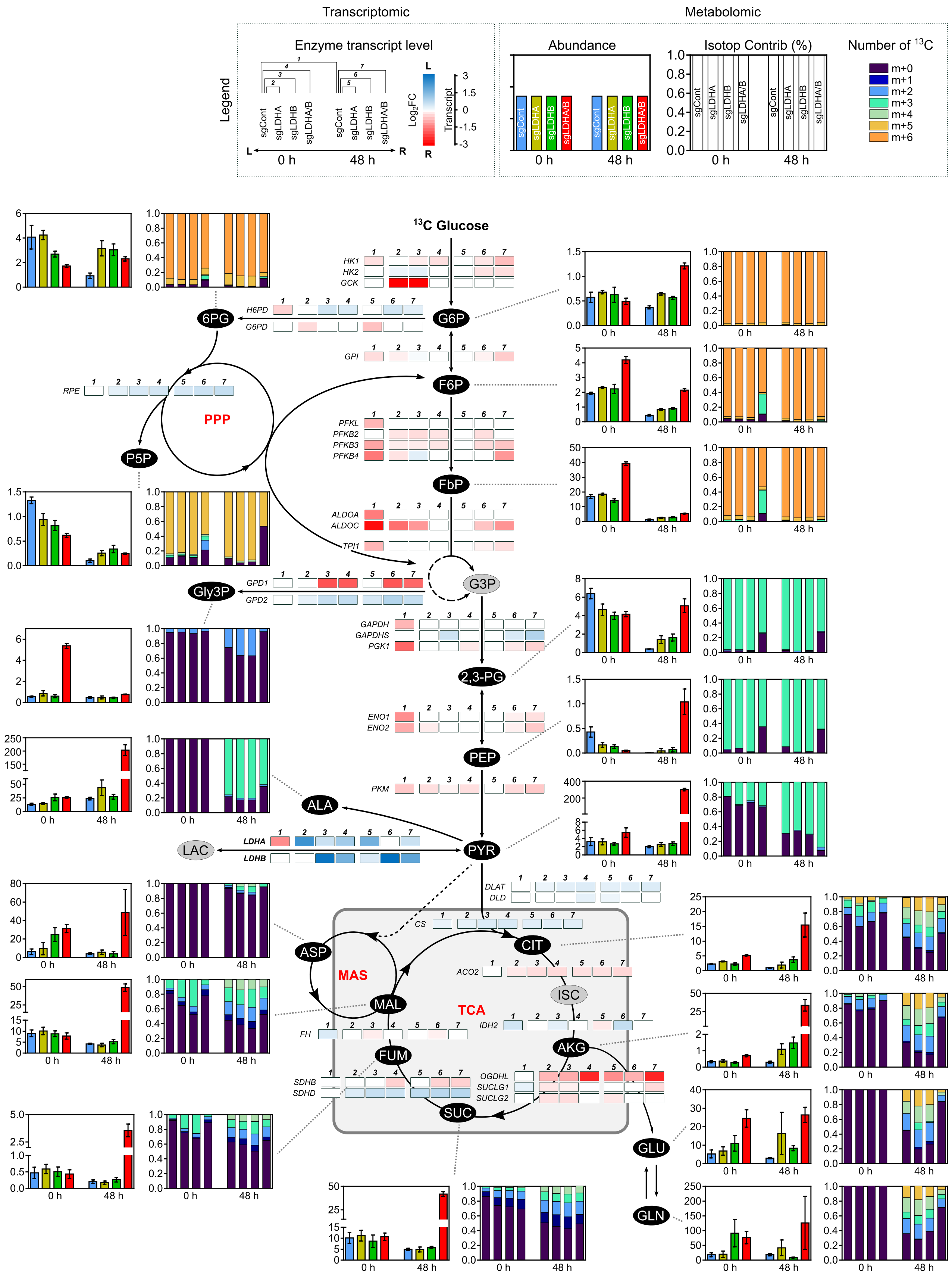

Figure EV5.

© 2022 The Authors
