# Supplementary material for: Lactate dehydrogenases promote glioblastoma growth and invasion via a metabolic symbiosis
Source: EMBO Mol Med. 2022 Oct 24;14(12):e15343. doi: 10.15252/emmm.202115343 (PMC9728051; doi:10.15252/emmm.202115343)
Supplement: Supplementary file 12 [file EMMM-14-e15343-s005.pdf]

EMBO Molecular Medicine

Lactate dehydrogenases promote glioblastoma growth and invasion via metabolic symbiosis

Joris Guyon, Ignacio Fernandez-Moncada, Claire Larrieu, Cyrielle Bouchez, Antonio Pagano Zottola, Johanna Galvis, Tiffanie Chouleur, Audrey Burban, Kevin Joseph, Vidhya Ravi, Heidi Espedal, Gro Rosland, Boutaina Daher, Aurelien Barre, Benjamin Dartigues, Slim Karkar, Justine Rudewicz, Irati Romero-Garmendia, Barbara Klink, Konrad Grützmann, Marie-Alix Derieppe, Thibaut Molinié, Nina Obad, Céline LEON, Giorgio Seano, Hrvoje Miletic, Dieter Heiland, Giovanni Marsicano, Macha Nikolski, Rolf Bjerkvig, Andreas Bikfalvi, and Thomas Daubon
DOI: 10.15252/emmm.202115343

Corresponding authors: Thomas Daubon (thomas.daubon@u-bordeaux.fr) , Andreas Bikfalvi (andreas.bikfalvi@u-bordeaux.fr)

Review Timeline:

Transferred from Review Commons:	26th Oct 21
Editorial Decision:	2nd Nov 21
Author Correspondence:	8th Nov 21
Editor Correspondence:	9th Nov 21
Appeal Received:	3rd Aug 22
Editorial Decision:	7th Sep 22
Revision Received:	26th Sep 22
Accepted:	27th Sep 22

Editor: Lise Roth

Transaction Report:

This manuscript was transferred to EMBO Molecular Medicine following peer review at Review Commons.

(Note: With the exception of the correction of typographical or spelling errors that could be a source of ambiguity, letters and reports are not edited. Depending on transfer agreements, referee reports obtained elsewhere may or may not be included in this compilation. Referee reports are anonymous unless the Referee chooses to sign their reports.)

Review #1

1. How much time do you estimate the authors will need to complete the suggested revisions:

Estimated time to Complete Revisions (Required)

(Decision Recommendation)

More than 6 months

2. Evidence, reproducibility and clarity:

Evidence, reproducibility and clarity (Required)

In this manuscript by Guyon J. et al., the authors have demonstrated that in GBM the two isoforms of the enzyme Lactate Dehydrogenase (LDH), namely LDHA and LDHB, show preferential expression of LDHA in the tumor core while that of LDHB in the periphery using spheroid models and scRNAseq patient data. In P3 and BL13 stem-like models they have shown that high LDHA/B levels lead to high lactate secretion from the cells under hypoxic conditions which is responsible for spheroid growth and invasiveness by fueling the TCA cycle as an energy source, as shown by metabolic tracing experiments. Further, the authors show that double KO of LDHA and LDHA but not single KO of either A or B is responsible for reducing invasive tumor growth in mice as shown by orthotopic tumor experiments in mice. Moreover, by integrating metabolomic data generated by carbon tracing experiments and transcriptomic data generated by RNA Sequencing, the authors show that LDHA/B KO cells under hypoxic conditions, triggers a metabolic switch leading to upregulation of TCA and glycolysis related metabolites and transcripts. Components of the mitochondrial respiratory chain which were specifically upregulated were confirmed by Western Blotting and that inhibiting the high respiratory capacity by irradiation increases survival in mice. Lastly, they have shown that an LDH inhibitor could marginally increase survival in mice which is increased significantly with a combinational treatment with antiangiogenic treatment, bevacizumab.

This is an interesting study showing the role of LDHs in tumor progression using high throughput data analysis, state of the art biochemical techniques and other molecular biology tools. Although the role of LDHs in regulate tumor progression in GBM are well known, the mechanistic pathways studied using global metabolomics and transcriptomic analysis is interesting and provides useful datasets in the field. Also, though the study is thoroughly conducted and data is mostly well presented, some of the data needs to be addressed further, most importantly regarding the mechanistic role of lactate through the respiratory chain complex as inhibiting the pathway did not affect survival in mice, and also, how does the anti-tumorigenic role of LDHB relate to the tumorigenic function of the double KO. Based on some of the issues mentioned below, the overall enthusiasm for publishing this manuscript in the EMBO journal in medium.

3. Significance:

Significance (Required)

Comments on both aspects:

1. In Figure 1, the authors should elaborate on their results better. For example, does the corpus callosum region highlighted in fig 1a portray the invasive region of the tumor as it is covered by the tumor periphery. Are both LDHA and LDHB expressed in this region? What does the dotted line indicate? Please mention in the legend. If yes, how is this region different from the periphery region which predominantly expresses LDHB. Also, in fig. s1b, please explain how LDHA and B expression is correlated to anatomical origin of tumor and which regions represent the core and peripheral regions? Is the positive correlation between LDHA and HIFA observed in any particular anatomic locations in this analysis?
2. The survival curves in fig. s1c are only marginally significant. The authors should check for survival analysis in other publically available GBM datasets as well.
3. In the metabolic tracing experiment, the amount of labelled lactate and pyruvate reached maximum levels between 0 to 5hrs showing that almost all of the lactate and pyruvate molecules in the cells become labelled within this time (Fig. S3b). However, in Figure S3c, the incorporation of labelled carbon does not appear to be very high within 0 to 5hrs as compared to citrate, which shows early incorporation in fig. s3c while achieves maximum activity in the exometabolome late around 24 hrs. What is the incorporation rate in citrate in the endometabolome? Does it match with its maximum level time point in fig s3b? Please comment on this discrepancy. What do the values m+0 to m+6 indicate? Please specify in the figure legend.
4. The data is figure 2i is interesting where the authors show that lactate is used as an energy source via TCA cycle through metabolic tracing experiments. Here, the authors should also inhibit one of the crucial components of the TCA cycle such as malate produced by the malate aspartate shuttle, which appears to have a high rate of labelled carbon incorporation, and then measure the labeled glutamine levels in the exometabolome.
5. In Fig. 3e and S4f, how do the authors explain the higher invasives in P3 cells upon LDHB KO? Does KO of LDHB shuttles the lactate production to LDHA which causes increase in invasiveness? However, in Fig. 3c, KO of LDHB shows low lactate production. Please comment!
6. In Fig. 3i, the authors state that reduced survival in case of LDHB KO is likely due to hemorrhage at the tumor site, however in Fig. S1c, patient survival data, there is a similar pattern of worse survival in case of low LDHB expression. Also, LDHB KO increases invasiveness in Fig. 3. Also, VEGF increase only in the LDHB KO tumor (Fig. S4g) could also be due to the anti-tumorigenic activity of LDHB and not just hemorrhage. Overall, LDHB appears to have an anti-tumorigenic function. However, double KO of LDHA and LDHB is increases survival indicating their cumulative action to be pro-tumorigenic. Though the doble KO data is convincing, single KO of LDHB does not add to the hypothesis. Please comment on the above.
7. In Fig. 3g,h, the authors should check the LDHA and LDHB levels in the tumor core and periphery to validate their localization in the tumor mouse model.
8. In 4c left panel, control cells, the majority of transcripts related to glycolysis increased whereas the majority of metabolites related to glycolysis decreased. Why isn't there a similar pattern between metabolome and transcriptome? Although a similar pattern is observed in the middle and right panels.
9. In Figure 5, although the components of the respiratory chain were validated to be high, inhibiting the respiratory complex I did not decrease tumor formation. Did the authors try to use inhibitors against the glycolytic pathways which are also seen to be upregulated in

LDHA/B KO in Figure 4.
10. In Fig. 6d, LDHA/B KO upregulates many components of the TCA cycle as all the pyruvate is pumped into the TCA cycle. However how does LDHA/B KO upregulate enzymes of the glycolytic cycle?
11. Fig. S6c is not readable. Please explain the values m+0 to m+10 in figure legend or text of Figure S7.
12. In Figure 6, the drug Stiripentol, which decreases LDH activity does not show a significant survival difference, however, combinational treatment with VEGF inhibitor Bevacizumab shows significant difference. Also, the difference between Bevacizumab only and combination treatment is only slightly significant, attributing all the survival difference to Bevacizumab and not Stiripentol. Thus, this data is not very convincing. Have the authors done a dose dependent study to test for Stiripentol IC50 values and toxicity in P13 cells? A cobinational treatment with higher dose of Stiripentol along with irradiation could be attempted here.
Minor
1. In Figure 1b,c, matrix embedded spheroid model, the authors should provide a quantification of the number of cells expressing either LDHA or LDHB in both the core and peripheral regions of the model.
2. Please provide figure numbers to the main and supplementary figures.
3. In Fig. S4e, please plot the significance of double KO Annexin-V staining with respect to control.
4. Please mention and explain Fig. S6a in the text.

Review #2

1. How much time do you estimate the authors will need to complete the suggested revisions:

Estimated time to Complete Revisions (Required)

(Decision Recommendation)

Between 3 and 6 months

2. Evidence, reproducibility and clarity:

Evidence, reproducibility and clarity (Required)

In this study by Guyon et al, the authors demonstrate that targeting both LDHA and LDHB that deferentially express in glioblastoma (GBM) cells and induced by hypoxia attenuates invasion and tumor progression of GBM. The authors performed metabolomics and transcriptomics analyses using cells lacking LDHA/B and demonstrated that knockout of LDHA/B results changes in gene expression and metabolites in GBM cells under hypoxic conditions. Genetic or pharmacological inhibition of LDH alone or in combination with irradiation further enhanced GBM tumor growth inhibition and enhanced survival both in vitro and in vivo. Overall, this study has identified an interesting link between LDHA/B,

hypoxia, and mitochondria metabolism, which is certainly interesting and relevant to the community who focus on cancer metabolism. However, several issues should be addressed through additional experiments and clarification to provide firmer mechanistic evidence in support of the major conclusions.

1. Quantification of distinct localization of LDHA and LDHB is needed for Figure 1 along with markers for hypoxic region and peripheral region of the tumor. In addition, colocalization of LDHA/B should be quantified and graphically presented.
2. What is specifically inducing LDHA not LDHB under hypoxia (Figure 2a)? Why same concentration (20 mM) of external lactate attenuate proliferation but promote GBM cell invasion (Figure 2e-g)? Does this difference brings back to the switch between glycolysis and OXPHOS. The authors at least should discuss potential underlying mechanisms.
3. The authors distinguished LDH activity and LDHB activity in LDHA/B knockout cell lines. However, it is unclear how the activities of LDHs and LDHB were distinguished. The legend states that they were colorimetric analyses but should be described in more detail. If LDHB activity was by using enriched LDHB through immunoprecipitation, the same approach should be made for LDHA. On a related note, the authors observed minimum effect on spheroid growth when LDHB was deprived. It is worth to know the expression levels of LDHA and LDHB in GBM cells or patient tumors.
4. It is unclear why only ETC1 inhibitor phenformin was used and compared to irradiation for tumor growth inhibition in LDHA/B KO cells in Figure 5d. Based on the immunoblot of Figure 5a, factors involved in ETC 2 (SDHA), 3 (UQCRC2), and 4 (COX II) were also increased. The authors need to check whether inhibitors against ETC 2/3/4 alter viability of GBM cells that lack LDHA and LDHB.
5. On a related note, it is unclear why the authors used LDH inhibitor and VEGF inhibitor in combination in Figure 6. Based on Figure 5, it seems targeting LDH in combination with radiation therapy is a good direction. It is necessary to provide why radiation and VEGF inhibition approaches were used in combination with LDH inhibition. In addition, both radiation and VEGF inhibitor should be treated in combination with the LDH inhibitor stiripentol and compare which would be more beneficial treatment for GBM. If there are any additional drugs that are routinely used in GBM, these need to be included in the combination study. Especially because the combination of VEGF inhibitor and LDH inhibitor seems not beneficial at all in vivo.
6. The authors state in their abstract that lactate is the metabolite that replenishes the TCA cycle, and this is based on the metabolomics data obtained using LDHA/B KO. How are the authors sure that all these changes including alteration of metabolites and metabolic gene expression (Figure 4c) are due to lactate but no other metabolites in cells lacking LDHA/B? Unless the authors demonstrate that replenishment of lactate restore these changes, it is hard to conclude that the driver was lactate.

3. Significance:

Significance (Required)

Please see above comments.

Review #3

1. How much time do you estimate the authors will need to complete the suggested revisions:

Estimated time to Complete Revisions (Required)

(Decision Recommendation)

Between 3 and 6 months

2. Evidence, reproducibility and clarity:

Evidence, reproducibility and clarity (Required)

Summary

The publication of Guyon et al reports that LDHA and LDHB contribute to the growth and invasion of glioblastoma stem-like cells by supporting the TCA cycle to maintain energy metabolism in hypoxic tumor areas. By creating single or double-knockout LDHA/B GBM cell lines as well as by metabolomics and transcriptional analyses, the authors identify changes of genes and metabolites involved in energy metabolism and report that lactate fuels oxidative phosphorylation in order to sustain tumor development. While LDHA and LDHB alone were shown to be insufficient therapeutic targets, a combined knockout of LDHA and LDHB significantly inhibited the malignant phenotype of patient-derived GBM stem-like cells. In addition, in vivo mouse studies with intracranial tumor injection with LDH activity inhibited by the antiepileptic drug stiripentol in combination with the antiangiogenic drug bevacizumab were performed. Guyon et al conclude that the activity of LDHA and LDHB in GBM is a potential new target for glioblastoma therapy when combined with other drugs or irradiation.

Major comments

The introduction of the publication does not cover all relevant information. First, the authors only describe the two main LDH subtypes LDH-A and LDH-B and leave out the other two LDH subtypes LDH-C and LDH-Bx. Furthermore, in the human body LDH exists in five different isozymes composed of four subunits, i.e. LDH-1 to LDH-5 (Drent et al, 1996; DOI: 10.1183/09031936.96.09081736). Depending on the composition of the LDH isozyme available in the brain, more information can be drawn from the presented data. The publication of Bittar et al from 1995 for instance describes the dominant LDH isozymes in astrocytes and neurons (DOI: 10.1097/00004647-199611000-00001). Further information is necessary for an improved interpretation of the published data.
In general, some information in the introduction is not cited at all. Other passages could be removed since they are not important for the story and hence are confusing (i.e. the information about NMDAR is not required).
A reagent list cannot be found in the supplemental documents.

Figure 1 shows the comparison of a healthy mouse brain with human GBM stem-like cells. This comparison is not suitable because different species can express a different pattern of LDH isozymes. Furthermore, since the brain is one of the most complex organs, it is unclear which area of the brain was used as control and if there is a general difference in the LDH expression pattern in different brain areas. An age-dependent change of LDH expression should also be excluded by appropriate experiments. Were the results confirmed with another GBM cell line, i.e. BL13 cells? The age of the mice and the number of tested animals should be specified. Statistical analysis of biological replicates should be included. At present the text does not explain why the spheroid model was used in addition to the in vivo model. One may argue that in the in vivo model it is unclear whether the cells at the invading edge are tumor cells or healthy therefore, the spheroid model is important and shows that the peripheral tumor cells show a different LDH composition that the ones in the tumor core. This should be explained in the text. In general the text can be impoved. In figure 2 only the activity of total LDH and of LDHB is shown, but information about the activity of LDHA is missing. Since other LDH subunits than only LDHB and LDHA could be present, the activity of LDHA should be included.
In figure 3 it is unclear if the hypoxic condition was at 0.1 % O2 (3a and 3f) or at 1 % O2 (3b). In addition, the oxygen concentrations in the figure differs from the indicated values in the figure description. The hypoxic conditions varies in the publication from 0.1 % O2 to 1% O2 (figure 4) and can therefore not be compared. Any explanation of the different conditions is missing.
Presented western blot data should be quantified according to figure 2a.

Figure 3f is not required for a standard in vivo experiment, the method description is sufficient.

It is striking that mice bearing LDHB knockout tumors show significantly lower survival than those with a knockout of LDHA and LDHA/LDHB (Fig 3i) but the tumor core and tumor invasion (Fig 3b) did not show differences. What is the reason for this observation? In the discussion the authors describe hemorrhages at the tumor site of mice bearing LDHB KO tumors. This should be supported by appropriate data and also dealt with in the results and more extensively in the discussion. Were hemorrhages also present in the double knockouts and if not, why not?

The data shown in 3g and h raises various questions:
How is the tumor core defined? How do you differentiate between tumor core cells and invasive GBM cells? The indicated areas for the cell invasion, i.e. the red square in the histological images, do not cover the complete dark region (assumed as the invasive area), why did you exclude the rest? The quantification is unusual and unclear and therefore difficult to assess.
How old were the mice in g and h, were they all sacrificed at the same day after tumor injection? The unit of the tumor core and the tumor invasion, meaning in relative to brain, is unusual and needs further explanation.

While 3g compares the single knockouts with the control, 3h only compare the double knockout with the control. How do single and double knockouts compare?

Figure 4 shows the metabolic and transcriptomic status of the created single and double-knockout LDHA/B cell lines and could be improved by including the metabolic and transcriptomic data of these cell lines under normoxic conditions, i.e. in a supplemental

figure.
Phenformin inhibits the oxidative phosphorylation in cells thereby altering their energy metabolism. Since this effect impacts not only tumor cells but the whole body, please add additional information on the impact of phenformin in healthy individuals. The used concentration of phenformin in figure 5 is not explained in the paper. Was the applied concentration determined in an in vitro pretest? Was it found in other publications? How can the authors conclude that the employed concentration was sufficient to inhibit oxidative phosphorylation? Further information are required.
In figure 6 the combined treatment of bevacizumab and stiripentol is shown. How efficient is the inhibition of the LDH activity in the cell lines by stiripentol? This could be easily investigated by a dose-dependent in vitro experiment.

In order to determine the effect of the combined treatment, the combo needs to be statistically compared to both single treatments, not only to the vehicle. Figure 6f and the tumor core data of 6g show that mainly bevacizumab is important for a better survival of the animals. This has to be stated in the manuscript and also modified in the abstract.

As mentioned above, further information about the calculation of the tumor core and the tumor invasion is missing as well.
Does the combination of LDHA/B knockout with bevacizumab show a combined effect?
And
In general, in figure 1 it was shown that spheroids have a different LDH expression pattern at different regions. The comparison of injected spheroids displaying regional-dependent LDH expression patterns with the metabolic and transcriptional analysis of cultivated GBM cells in suspension could greatly differ in their metabolism and thereby not reflect the situation in the tissue.

Minor comments

The text needs to be checked for grammatical errors. The experiments should be described in a way that they can be reproduced. References should be checked and included where they are missing, i.e. in the introduction. Hypoxic conditions should be made clear to avoid any confusion. In general, check if all necessary information for each figure is included in the figure and the figure description, do not exclusively insert important information in the main text.

3. Significance:

Significance (Required)

The manuscript is of moderate novelty. The presentation of lactate accumulation and the increased activity of LDH under hypoxia is presented for the specific cancer type GBM. Nevertheless, most of the information is already known for most cancer types (i.e. Warburg effect). The main significance of this paper relies in the potential co-therapeutic targeting of LDHA and LDHB in GBM patients.

My expertise

Long-standing experience working on amino acid metabolism in glioblastoma.

Revision Plan

Manuscript number: RC-2021-00982
Corresponding author(s): Thomas, Daubon

1. General Statements

Our article was globally appreciated by the 3 reviewers and we believe that explanations given in part 2 of the Revision Plan will convince the reviewers that our story is of importance for a broad scientific audience. We would like to thank the reviewers for the positive comments about our manuscript and we would like to bring more information about the significance and novelty of our work. Reviewer suggested that the role of LDH in GBM growth regulation was well studied but only few studies were related to this topic: Seliger et al 2013 and Kim et al 2015. More importantly, lactate as metabolite has never been studied in such great details in GBM development and especially in GBM invasion.
Importantly, our article does not rely only on Warburg effect, as raised by reviewer 3, which is the adaptation of cells after hypoxia, but also rely on lactate symbiosis between hypoxic cells and oxygenated areas. Metabolic symbiosis is of great importance, as discussed by several scientific teams in the world (*e.g.* important review of Corbet and Feron “Tumour acidosis: from the passenger to the driver’s seat”). Here, we show that GBM cells cultured in hypoxia can fuel cells from oxygenated area by feeding them with lactate, to trigger cell invasion, in a symbiotic way. This is the first time that the role of both LDHA and LDHB is described into details in glioblastoma by using RNA sequencing coupled with ^{13}C metabolomics. We also discuss in great details how LDHA/B KO cells, meaning without lactic fermentation, adapt in severe hypoxia. This is the first time that regulation of respiratory chain complex expression is shown in that context.

2. Description of the planned revisions

The responses to reviewers were written in blue and the proposed experiments in green in the text.

<u>**Reviewer #1** (Evidence, reproducibility and clarity (Required)):</u>

In this manuscript by Guyon J. et al., the authors have demonstrated that in GBM the two isoforms of the enzyme Lactate Dehydrogenase (LDH), namely LDHA and LDHB, show preferential expression of LDHA in the tumor core while that of LDHB in the periphery using spheroid models and scRNAseq patient data. In P3 and BL13 stem-like models they have shown that high LDHA/B levels lead to high lactate secretion from the cells under hypoxic conditions which is responsible for spheroid growth and invasiveness by fueling the TCA cycle as an energy source, as shown by metabolic tracing experiments. Further, the authors show that double KO of LDHA and LDHA but not single KO of either A or B is responsible for reducing invasive tumor growth in mice as shown by orthotopic tumor experiments in

mice. Moreover, by integrating metabolomic data generated by carbon tracing experiments and transcriptomic data generated by RNA Sequencing, the authors show that LDHA/B KO cells under hypoxic conditions, triggers a metabolic switch leading to upregulation of TCA and glycolysis related metabolites and transcripts. Components of the mitochondrial respiratory chain which were specifically upregulated were confirmed by Western Blotting and that inhibiting the high respiratory capacity by irradiation increases survival in mice. Lastly, they have shown that an LDH inhibitor could marginally increase survival in mice which is increased significantly with a combinational treatment with antiangiogenic treatment, bevacizumab.

This is an interesting study showing the role of LDHs in tumor progression using high throughput data analysis, state of the art biochemical techniques and other molecular biology tools. Although the role of LDHs in regulate tumor progression in GBM are well known, the mechanistic pathways studied using global metabolomics and transcriptomic analysis is interesting and provides useful datasets in the field. Also, though the study is thoroughly conducted and data is mostly well presented, some of the data needs to be addressed further, most importantly regarding the mechanistic role of lactate through the respiratory chain complex as inhibiting the pathway did not affect survival in mice, and also, how does the anti-tumorigenic role of LDHB relate to the tumorigenic function of the double KO. Based on some of the issues mentioned below, the overall enthusiasm for publishing this manuscript in the EMBO journal in (is) medium.

Reply: We thank the reviewer for the positive comments and time spent to evaluate our manuscript and we would like to bring more information about the significance and novelty of our work. Reviewer suggested that the role of LDH in GBM growth regulation was well studied but, to our knowledge, only few studies were related to this topic: Seliger et al 2013 and Kim et al 2015. More importantly, lactate as metabolite has never been studied in such great details in GBM development and especially in GBM invasion.

Reviewer #1 (Significance (Required)):

Comments on both aspects:

1. In Figure 1, the authors should elaborate on their results better. For example, does the corpus callosum region highlighted in fig 1a portray the invasive region of the tumor as it is covered by the tumor periphery. Are both LDHA and LDHB expressed in this region? If yes, how is this region different from the periphery region which predominantly expresses LDHB.

Reply: We thank the reviewer for these comments and we have improved the explanations of this figure in the text.
The corpus callosum region is part of the invasive area, as depicted in the image below with the red line:

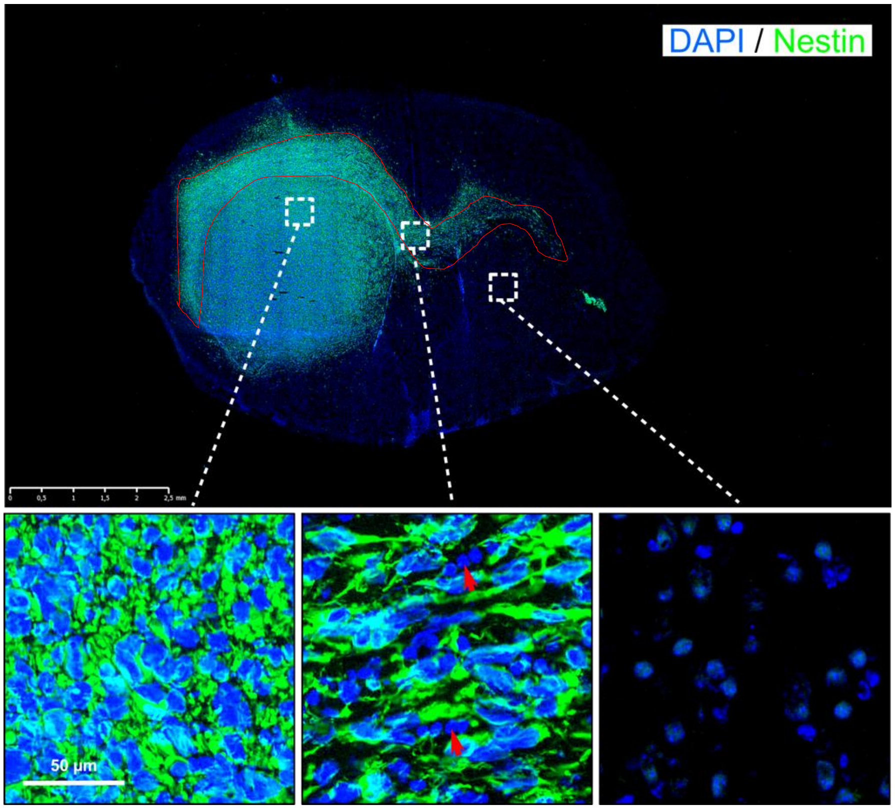

This area in the corpus callosum differs from the periphery area by the microenvironment, mainly composed of myelinated axons and giving access to the contro-lateral brain. Of note, GBM cells are influenced by this microenvironment and acquire elongated/invasive phenotype (lower middle image). Both LDHA and LDHB are expressed in this region but as now better indicated in the text and in the legend, some cells only express one or the other (as shown by the dotted lines in **Figure 1c**) and in the image below:

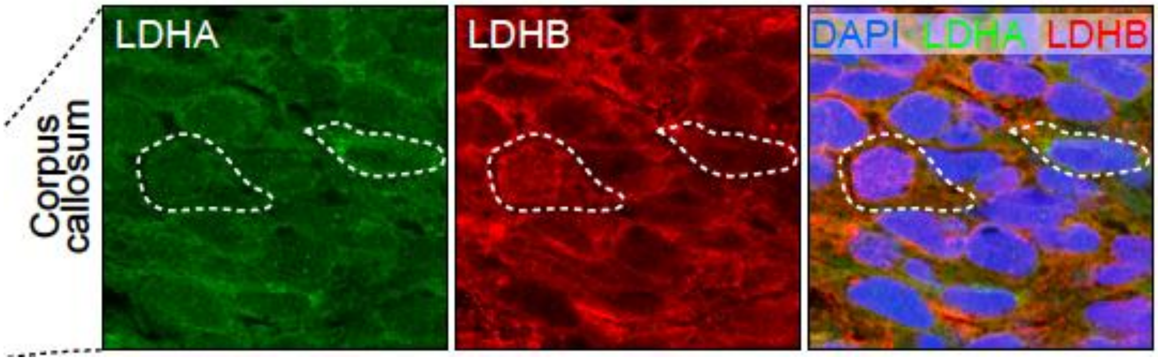

As shown below, LDHA and LDHB are both expressed in corpus callosum:

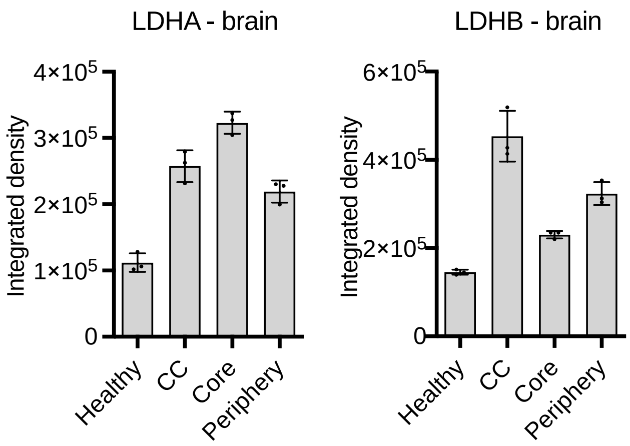

This is better explained in the text.

What does the dotted line indicate? Please mention in the legend.
This has been removed from Figure 1, as we quantified the intensities of LDHA and LDHB (see above).

Also, in fig. s1b, please explain how LDHA and B expression is correlated to anatomical origin of tumor and which regions represent the core and peripheral regions? Is the positive correlation between LDHA and HIFA observed in any particular anatomic locations in this analysis?
In Figure s1b, LDHA is more expressed in infiltrative area (IT) and in cellular tumor with microvascular proliferation (CT mvp), corresponding to areas we defined as invasion area and to some hypoxic/central area, respectively. Globally, LDHA is more expressed in samples with high HIF1a expression as described. Of note, hypoxic areas cannot be sequenced by RNA sequencing, due to low RNA quality. Then, LDHB has no specific expression in the IVYGAP database but is globally less expressed in areas with high HIF1a expression. This has been better explained in the main text.

2. The survival curves in fig. s1c are only marginally significant. The authors should check for survival analysis in other publically available GBM datasets as well.
Reply: We thank the reviewer for this comment and we are generating other survival curves based on alternative database (Genomic Data Commons -GDC- Data Portal, using TCGA, as we did, but also Target database). Our bioinformatician is working on it and we will insert this alternative survival curves in the final version of our manuscript. Of note, most of survival curves generated with glioblastoma patient (not with lower grades) give few differences due to dramatic prognosis. Nevertheless, we generated graphs with survival curves based on low grade glioma and the same trend was found for LDHA and LDHB as poor or good prognosis factors, respectively:

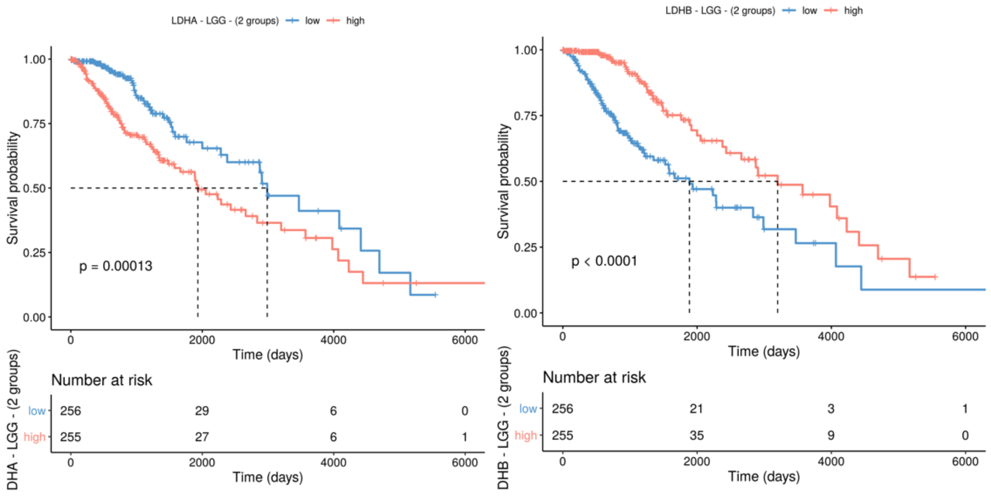

This was added in Suppl Figure 1c.

3. In the metabolic tracing experiment, the amount of labelled lactate and pyruvate reached maximum levels between 0 to 5hrs showing that almost all of the lactate and pyruvate molecules in the cells become labelled within this time (Fig. S3b). However, in Figure S3c, the incorporation of labelled carbon does not appear to be very high within 0 to 5hrs as compared to citrate, which shows early incorporation in fig. s3c while achieves maximum activity in the exometabolome late around 24 hrs. What is the incorporation rate in citrate in the endometabolome? Does it match with its maximum level time point in fig s3b? Please comment on this discrepancy.

Reply: We would like to recapitulate the analysis done in this experiment.

The fractional contribution represents the contribution of ^{13}C atoms to all of the atoms in a specific metabolite. This implies that in the case of citrate, if we - in theory- could only label a maximum of 2 molecules out of the 6 molecules, the fractional contribution will “penalize” the actual contribution, hence display a lower number of labeling than it actually is.

Looking at the isotopologues, we could have for instance 100% labeling in the m2 of citrate, but the fractional contribution will only be 33%. Corrected isotopologues represent the actual “artificial” ^{13}C labeling. For small molecules like pyruvate, the chances are high that all carbons are labelled and then the formula of fractional contribution works, while larger molecules have carbons that cannot be labeled from specific sources, hence they have a lower % labeling. Then, incorporation rate of citrate in fig s3c corresponds to fractional contribulation of citrate in fig s3b.

Either we show the fractional contribution, or we show the isotopologues or a combination of both to make the point. Bart Guesquiere’s team (Metabolomics facility, Leuven, Belgium) is working on an improved formula for fractional contribution, but this depends from tracer to tracer.

Of note, to transform abundance to quantity (based on VIB metabolomics expertise): metabolite abundances cannot be compared with each other, this is due to the difference in ionisation potential of each molecule. However, we can compare the abundance of the same metabolite across different conditions, this gives a relative abundance. However, those technics are really expensive and time-consuming and we therefore think our model is of quality and relevant to answer our question.

To better explain the model, we generated the figure below, which can be inserted as supplemental material if reviewer 1 considers it useful:

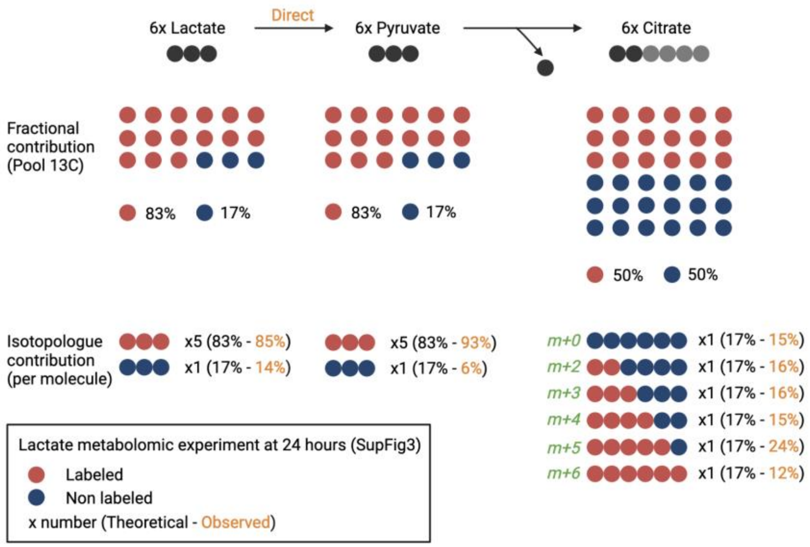

What do the values m+0 to m+6 indicate? Please specify in the figure legend.

→ m+0 stands for the fraction of metabolite without ^{13}Carbon and m+n (n > 0) stands for fraction of metabolite with n ^{13}Carbon. For example, m+5 correspond to a metabolite with 5 labeled ^{13}Carbon. The sum of (m+0, m+1,…, m+10,…) equals to 1.

This has been added into the legends.

4. The data is figure 2i is interesting where the authors show that lactate is used as an energy source via TCA cycle through metabolic tracing experiments. Here, the authors should also inhibit one of the crucial components of the TCA cycle such as malate produced by the malate aspartate shuttle, which appears to have a high rate of labelled carbon incorporation, and then measure the labeled glutamine levels in the exometabolome.

Reply: We thank the reviewer for this interesting proposal. To our knowledge thanks to part of our research team are experts on it (Article in preprint: DOI:10.1101/2021.04.17.440272), inhibiting malate-aspartate shuttle (MAS) by using MDH inhibitors is not working. Another inhibitor massively used for inhibiting the MAS, *via* GOT inhibition, is aminooxiacetate (AOA) but again, this is unspecific and inhibits all transaminases which will directly impair glutamate and then glutamine production (we provided a figure with detailed malate-aspartate shuttle and pathways directly linked to glutamine to illustrate our reply).

Revision Plan

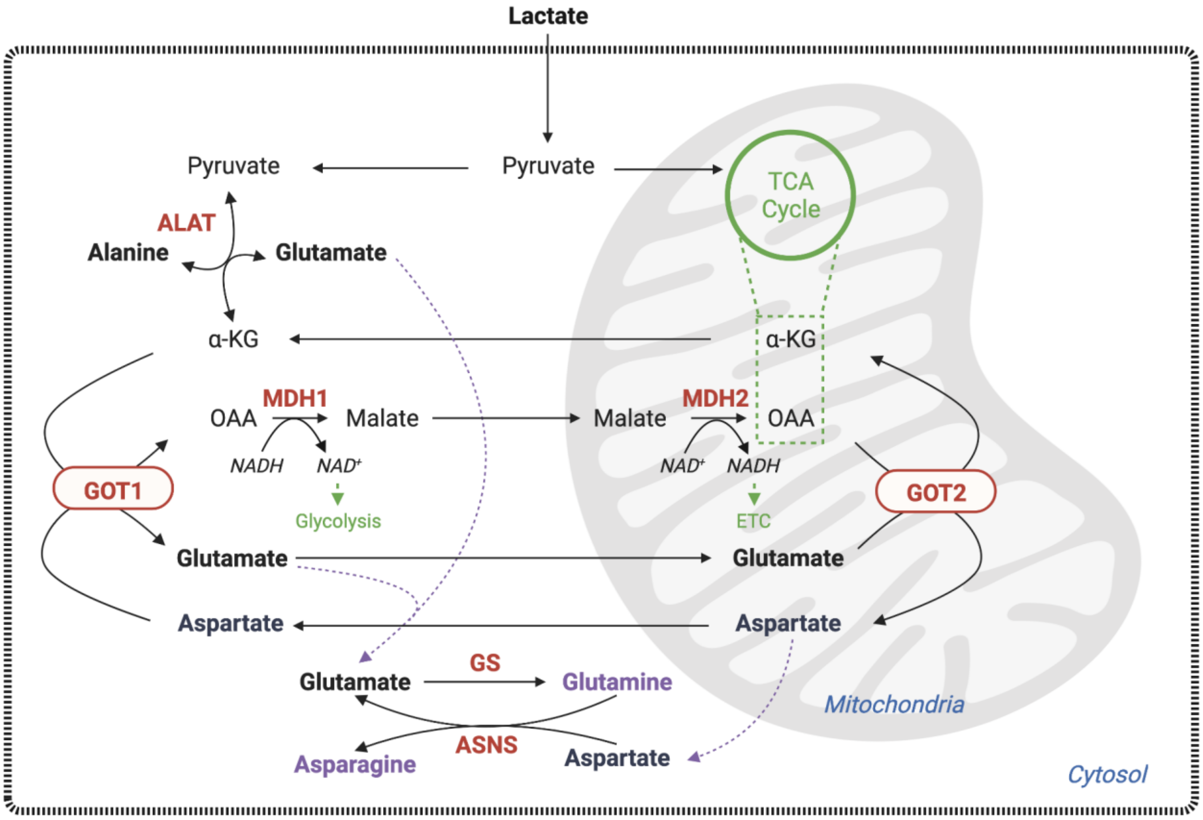

We can propose to use Cycloserine for inhibiting specifically GOT1 and measure glutamine level in the exometabolome, to answer the question raised by the reviewer.

5. In Fig. 3e and S4f, how do the authors explain the higher invasives in P3 cells upon LDHB KO? Does KO of LDHB shuttles the lactate production to LDHA which causes increase in invasiveness? However, in Fig. 3c, KO of LDHB shows low lactate production. Please comment.

Reply: We thank the reviewer for this comment. As LDHB is KO in P3 sgLDHB, cells only express LDHA and this enzyme is known to be related to poor prognosis (**Suppl. Fig1c**). LDHA have a greater affinity for pyruvate than lactate and converts it into lactate to regenerate the redox potential of the cells allowing the maintenance of the glycolytic flux. Effectively, the intracellular lactate production rate in **Fig3c** is slightly lower for LDHB KO cells compared to control cells (0.05 min^{-1} *vs* 0.06 min^{-1}, respectively – **Source Data Fig3**) and in this experiment, cells were stationary, did not invade any matrix, experiment followed cells during 35 minutes and used different agents (oxamate, diclofenac). However, in **Fig3b**, lactate secretion in the extracellular compartment after 24 hours is greater for LDHB KO cells compared to control cells ($1.4x10^6$ RLU *vs* $1.1x10^6$ RLU **- Source Data Fig3**). LDHA KO cells express LDHB enzyme that have a greater affinity for lactate and when lactate levels increase and exceed pyruvate levels, LDHB would prefer to consume the formed lactate which was shown in Figure 2f/g/I to be efficiently used by the cells to improve invasion. Then, lactate accumulation in the extracellular compartment is a pro-invasive metabolite, as shown in **Figure 2f-g**.

6. In Fig. 3i, the authors state that reduced survival in case of LDHB KO is likely due to hemorrhage at the tumor site, however in Fig. S1c, patient survival data, there is a similar pattern of worse survival in case of low LDHB expression. Also, LDHB KO increases invasiveness in Fig. 3. Also, VEGF increase only in the LDHB KO tumor (Fig. S4g) could also be due to the anti-tumorigenic activity of LDHB and not just hemorrhage. Overall, LDHB appears to have an anti-tumorigenic function. However, double KO of LDHA and LDHB is increases survival indicating their cumulative action to be pro-tumorigenic. Though the doble KO data is convincing, single KO of LDHB does not add to the hypothesis. Please comment on the above.

Reply: We think that the *in vivo* results of LDHB are of high importance. As the reviewer commented, LDHB appears to be pro-tumoral in patient data and the results we obtained are aligned with this finding. We showed that inhibiting LDHB expression leads to accumulation of extracellular lactate and protons (**Figure 3b**), inducing activation of many pathways via growth factors (TGFbeta, VEGF), and an increase in GBM aggressiveness, as mentioned in several papers from Pierre Sonveaux group (doi: 10.1158/0008-5472.CAN-10-2828 ; https://doi.org/10.1371/journal.pone.0033418). Thus, accumulation of extracellular lactate in KO LDHB cells lead to local acidosis which activates VEGF, as shown in **Figure S4g**. VEGF is one of the most efficient growth factor to induce neoangiogenesis and in high concentration, blood vessel leakiness.
Text has been modified in the discussion to bring more information.

This reply is linked to the reply from **Reviewer 3 point 6** in which blood vessel were quantified.

7. In Fig. 3g,h, the authors should check the LDHA and LDHB levels in the tumor core and periphery to validate their localization in the tumor mouse model.

Reply: We thank the reviewer for this comment and the experiments will be done. We would like to emphasize that the double staining of LDHA and LDHB is a complicated procedure with very specific tissue fixation, which was not adapted to the first procedure employed in these experiments. We will generate a new implantation experiment with all cell lines transduced with GFP and will provide LDHA/LDHB staining and quantification in the final revised manuscript.

8. In 4c left panel, control cells, the majority of transcripts related to glycolysis increased whereas the majority of metabolites related to glycolysis decreased. Why isn't there a similar pattern between metabolome and transcriptome? Although a similar pattern is observed in the middle and right panels.

Reply: We agree with the reviewer these discordant patterns between transcriptome and metabolome were striking. However, it is important to mention that enzymes transcript levels, but also protein itself, are not reliable surrogate for metabolite consumption/production. Heterogeneity might result from a complex system of factors that regulate metabolic fluxes. Mechanisms of regulation and kinetic parameters of distinct isoforms of an enzyme, which catalyze a similar metabolic reaction, differ and must be considered (Hakimi et al., Cell 2016). Nevertheless, a metabologram is a simplified visualization tool to review simultaneous transcriptomic and metabolomic data of different metabolic pathways, one at a time.

We can hypothesize that control cells efficiently use glycolysis in hypoxia, leading to no accumulation of metabolites but upregulation of most of enzymes linked to HIF1α (HKs, PFK1, ALDO, phosphoglycerate kinase, ENO1, PKM2 and LDHA).

9. In Figure 5, although the components of the respiratory chain were validated to be high, inhibiting the respiratory complex I did not decrease tumor formation. Did the authors try to use inhibitors against the glycolytic pathways which are also seen to be upregulated in LDHA/B KO in Figure 4.
Reply: This is an interesting point. We haven't done this experiment in our procedure but we can propose to treat control and KO LDHA/B spheroids with 2DG which bypasses the glycolysis and observe the viability (use of dead/live kit, in order), either in normoxia or hypoxia.
The first experiment is running this week and we will be repeated twice more to for reach good statistical level.

10. In Fig. 6d, LDHA/B KO upregulates many components of the TCA cycle as all the pyruvate is pumped into the TCA cycle. However how does LDHA/B KO upregulate enzymes of the glycolytic cycle?
Reply: This is a good comment from the reviewer. The experiments were performed in hypoxia where most of glycolytic enzymes are upregulated by the stabilization of HIF1α (HKs, PFK1, ALDO, phosphoglycerate kinase, ENO1, PKM2 and LDHA). To compensate a redox stress (unbalanced NADH/NAD+), cells are maintaining high level of glycolytic activity to compensate the absence of lactate. Moreover, as observed in **Figure 5b**, the size of the cells was detected lower in the double LDHA/B KO cells (as shown for enzymes from Krebs cycle) and then protein content is supposed to globally increase.
Here is the quantification of cell size:

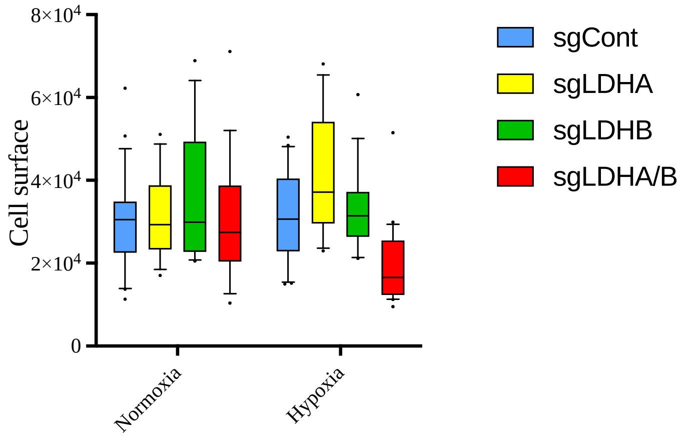

11. Fig. S6c is not readable.
Reply: This figure has been moved to new Figure S7.
Please explain the values m+0 to m+10 in figure legend or text of Figure S7.

Reply: This has been added into the legend: m+0 stands for the fraction of metabolite without ^{13}Carbon and m+n (n > 0) stands for fraction of metabolite with n ^{13}Carbon. For example, m+5 correspond to a metabolite with 5 labeled ^{13}C. The sum of (m+0, m+1,…, m+10,…) equals to 1.

12. In Figure 6, the drug Stiripentol, which decreases LDH activity does not show a significant survival difference, however, combinational treatment with VEGF inhibitor Bevacizumab shows significant difference. Also, the difference between Bevacizumab only and combination treatment is only slightly significant, attributing all the survival difference to Bevacizumab and not Stiripentol. Thus, this data is not very convincing. Have the authors done a dose dependent study to test for Stiripentol IC50 values and toxicity in P3 cells? A combinational treatment with higher dose of Stiripentol along with irradiation could be attempted here.
Reply: We thank the reviewer for this comment and we agree on the moderate effect of stiripentol when coupled with bevacizumab, but this effect was found statistically different when compared to control conditions. Of note, the concentration of Stiripentol used in our first in vivo experiment was 300 mg/kg but it induces a strong inhibition of mouse mobility, impacting mouse wellbeing, which was considered as excessive molecule reaction by the local ethical committee.

The implantation started this week and we propose this plan for answering this question:

- **Group 1** (8 mice) control treatment from Day 27, 3 times a week.
- **Group 2** (8 mice) Stiripentol 150 mg/ml from Day 27, 3 times a week.
- **Group 3** (8 mice) TMZ 25 mg/ml on Day 28 (AM + PM), Day 29 (AM + PM), Day 30 (AM) then X-ray 2 Gy Day 30 (PM), 2 Gy on Day 32 (PM) and 2 Gy on Day 35 (PM), then 1 day per week from Day 36 pf TMZ at 50 mg/ml.
- **Group 4** (8 mice) Stiripentol 150 mg/ml from Day 27, 3 times a week, then same treatment than group 3.

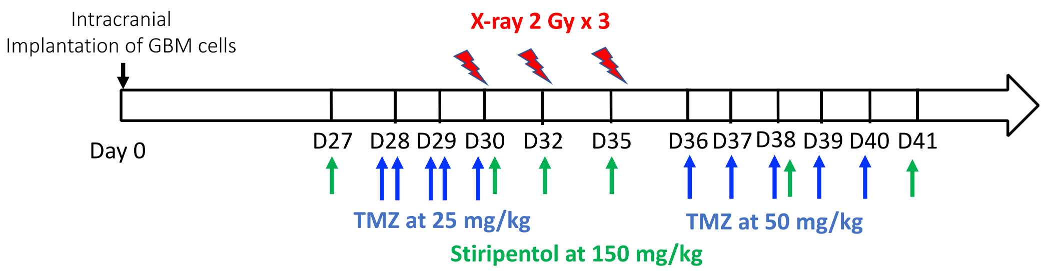

We will perform the *in vivo* experiment by treating animals with stiripentol and irradiation + temozolomide. The results will be obtained within 2 months.

Minor

1. In Figure 1b,c, matrix embedded spheroid model, the authors should provide a quantification of the number of cells expressing either LDHA or LDHB in both the core and peripheral regions of the model.

Reply: We have quantified the intensity of LDHA and LDHB expression in the spheroid areas as defined in Figure 1c, as we believe that this is more accurate than counting number of cells only positive for LDHA and/or LDHB.

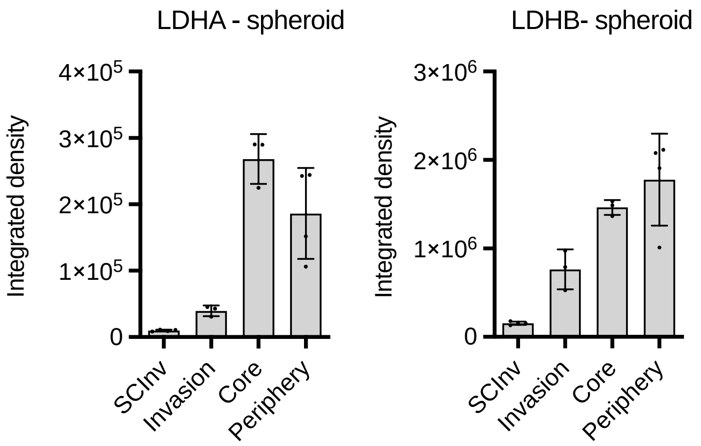

2. Please provide figure numbers to the main and supplementary figures.
Reply: This has been added in each figure.

3. In Fig. S4e, please plot the significance of double KO Annexin-V staining with respect to control.
Reply: This result was presented in the table of Suppl Fig4e. This is now better explained in the legend.

4. Please mention and explain Fig. S6a in the text.
Reply: Fig. S6a was mentioned in both sentences "To explore the relationship between transcriptomic and metabolomic data, transcript levels of metabolism-related genes were incorporated into the central carbon metabolic network pathway map (Fig 4d and supplementary Fig 6a-b)." and in "To corroborate the RNA sequencing results (Fig 4c-d and supplementary Fig 6a), but we added extra explanations in the main text.

Reviewer #2 (Evidence, reproducibility and clarity (Required)):

In this study by Guyon et al, the authors demonstrate that targeting both LDHA and LDHB that deferentially express in glioblastoma (GBM) cells and induced by hypoxia attenuates invasion and tumor progression of GBM. The authors performed metabolomics and transcriptomics analyses using cells lacking LDHA/B and demonstrated that knockout of LDHA/B results changes in gene expression and metabolites in GBM cells under hypoxic conditions. Genetic or pharmacological inhibition of LDH alone or in combination with irradiation further enhanced GBM tumor growth inhibition and enhanced survival both in vitro and in vivo. Overall, this study has identified an interesting link between LDHA/B, hypoxia, and mitochondria metabolism, which is certainly interesting and relevant to the community who focus on cancer metabolism. However, several issues should be addressed through additional experiments and

clarification to provide firmer mechanistic evidence in support of the major conclusions.
Reply: We thank the reviewer for these positive comments.

1. Quantification of distinct localization of LDHA and LDHB is needed for Figure 1 along with markers for hypoxic region and peripheral region of the tumor. In addition, colocalization of LDHA/B should be quantified and graphically presented.
Reply: As discussed in **Reviewer 1 (point 7)**, double staining of LDHA and LDHB required specific procedure of tissue fixation which led to complications for staining with other markers. We have already performed some image quantification of LDHA and LDHB staining, but new experiment will be executed for determining colocalization of HIF1a or CAIX with LDHA. However, we generate a large image of LDHA staining with nucleus marker (Hoescht) in which we can see a clear LDHA pattern in the central part of the tumor core:

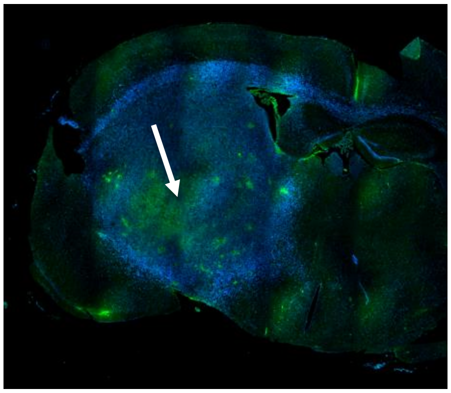

Peripheral region can be detected by using anti-Nestin staining as proposed in the image below. We can detect that tumor cells harbor bigger nuclei (green nuclei in the figure below, right panel) than cells from microenvironment (black nuclei in the figure below):

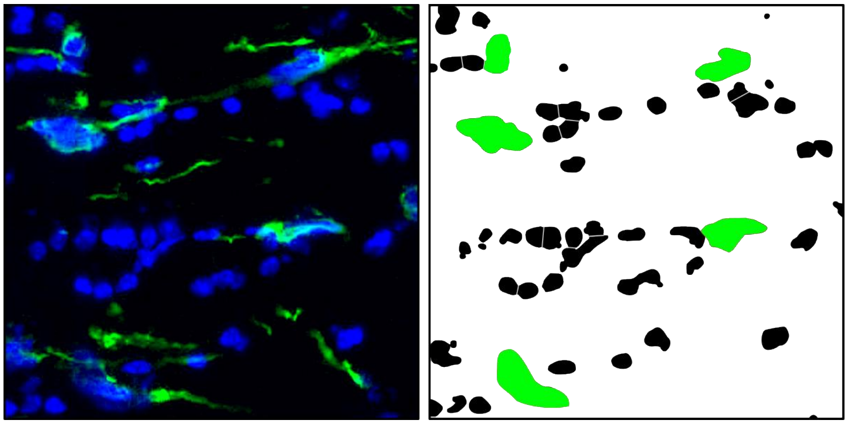

Colocalisation of LDHA/B was presented in Figure 1, we have also quantified intensity of LDHA and LDHB from the images. Results can be seen in **Reviewer 1 (Point 1)**.

2. What is specifically inducing LDHA not LDHB under hypoxia (Figure 2a)?
Reply: It has been previously characterized that *LDHA* promoter contains Hypoxia-Responsive Element (HRE) which is not present in *LDHB* promoter. *LDHB* has been characterized to be regulated by PGC-1a (https://www.jbc.org/article/S0021-9258(20)34525-7/fulltext).

Why same concentration (20 mM) of external lactate attenuate proliferation but promote GBM cell invasion (Figure 2e-g)?
We hypothesized that lactate favors invasion over proliferation as suggested in the go-or-grow hypothesis (https://doi.org/10.1016/j.bpj.2020.01.036) and in our observations, even if we can observe a smaller but efficient proliferation of lactate-treated cells when compare to control cells. We generated a longer proliferation recording to confirm results presented in Figure 2e:

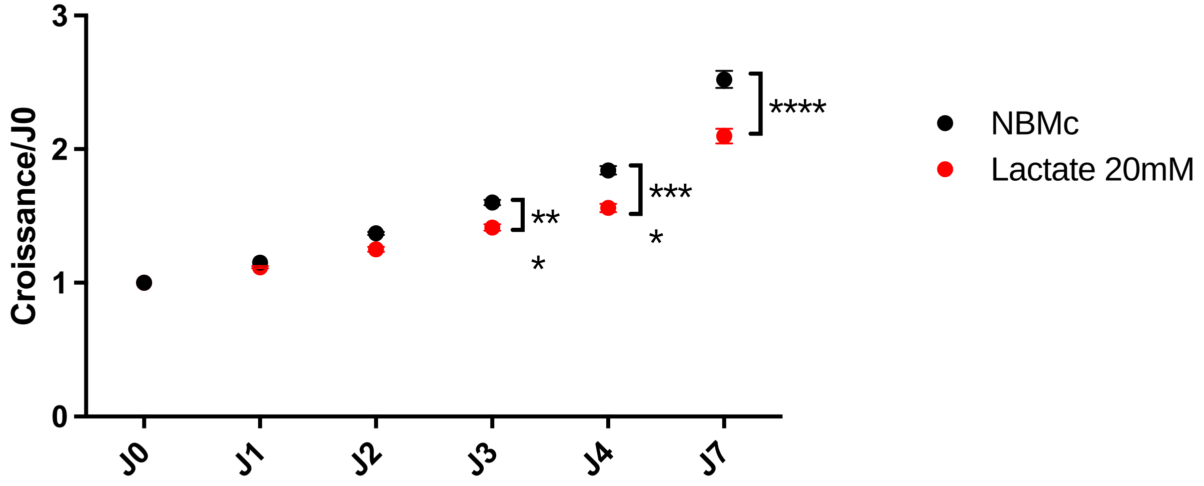

Does this difference brings back to the switch between glycolysis and OXPHOS. The authors at least should discuss potential underlying mechanisms.
We observed that rapidly after lactate infusion (<24h), lactate is directly fueling Krebs cycle for efficient energy production, then incorporation of 13C in metabolites present in neoglucogenesis was observed for sustaining biomass synthesis (unpublished results, from Suppl Fig 3), but is not sufficient in longer term to sustain proliferation as observed in glucose-enriched medium (NBMc above). Some modifications were inserted in the discussion to better discuss these points.

3. The authors distinguished LDH activity and LDHB activity in LDHA/B knockout cell lines. However, it is unclear how the activities of LDHs and LDHB were distinguished. The legend states that they were colorimetric analyses but should be described in more detail. If LDHB activity was by using enriched LDHB through immunoprecipitation, the same approach should be made for LDHA.
Reply: We apologize that we did not mention into details the two biochemical assays to record LDHs and LDHB activities. This is now added in the material and methods section. Briefly, LDH activity assay measures the formation of NADH in whole lysates (LDHA + LDHB isozymes) while LDHB activity was

measured by immunoprecipitating LDHB and measuring in the ELISA plate (LDHB isozymes alone), also by following NADH formation.

We agree with the reviewer and we can comment that no commercial kit has been developed for specifically measure LDHA activity. Then, we planned to use an equivalent system than developed for measuring LDHB activity.

The protocol will be based on:
- ELISA plate coated with anti-LDHA antibodies
- Lysates generated with the same lysis buffer than the LDHB kit
- Add the reaction products and use NADH to measure NAD+ by using spectrophotometry at 340 nm and signal decreases when NAD+ accumulates (as shown here on total lysates: DOI 10.1074/jbc.RA118.004180).

On a related note, the authors observed minimum effect on spheroid growth when LDHB was deprived. It is worth to know the expression levels of LDHA and LDHB in GBM cells or patient tumors.
Reply: As shown in **Figure 3A**, expression of LDHA and LDHB does not differ when the other subunit is depleted:

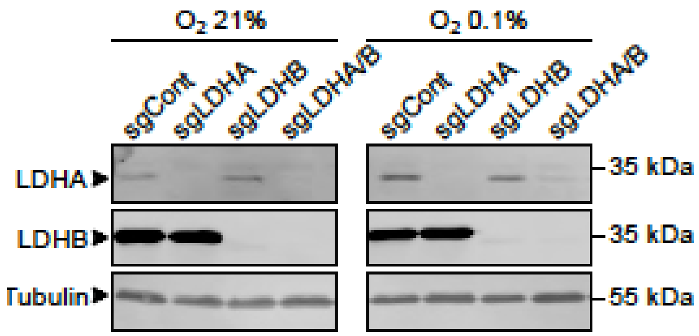

LDHB is highly expressed when compared to LDHA expression in P3 cells.
When checking LDHA and LDHB expression in ProteinAtlas database, we can observe that LDHB is also more expressed in glioma patients than LDHA (value of 183.4 FPKM for LDHB and of 99.2 FPKM for LDHA).

4. It is unclear why only ETC1 inhibitor phenformin was used and compared to irradiation for tumor growth inhibition in LDHA/B KO cells in Figure 5d. Based on the immunoblot of Figure 5a, factors involved in ETC 2 (SDHA), 3 (UQCRC2), and 4 (COX II) were also increased. The authors need to check whether inhibitors against ETC 2/3/4 alter viability of GBM cells that lack LDHA and LDHB.
Reply: We thank the reviewer for this interesting comment. Phenformin was used *in vivo* for reproducing results from **Figure 2g** when rotenone was used in spheroid invasion assay. Our aim was to specifically inhibit both regulators of redox in the tumor cells (lactate dehydrogenases and ETC1). Then, we can propose to measure cell viability, by using dead/live kit, in control and LDHA/B KO cells when adding inhibitors of ETC 2/3/4 such as Aptenin A5, antimycin A and cyanide, respectively.

However, inhibiting ETC 3 and 4 will lead to toxic effects as antimycin A induces letal accumulation of ROS and cyanide inhibits all peroxidases. To conclude, antimycin A and cyanide will have pleiotropic effects which will hide a precise answer.

We have already evaluated the inhibition of ETC2 by Aptenin A5 on cell respiration. We observed a strong sensitivity to inhibitors of complex I and not of complex II (extra results from respirometry of **Figure 5c**).
Below are the results in which we can observe 90% respiratory flux is directly inhibited by rotenone, as specific inhibitor of ETC1 (graph from above is related to cells with either complete medium, only lactate or only glucose ; graph from above is related to KO LDHA/B cells):

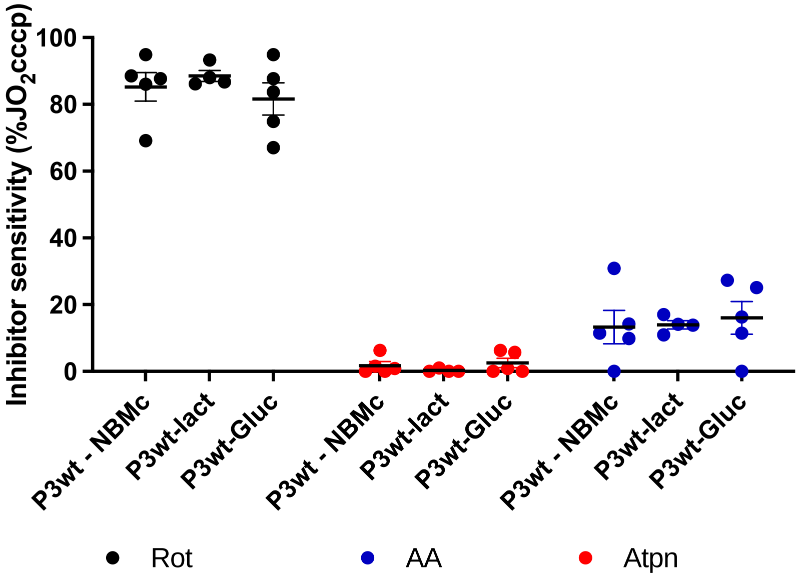

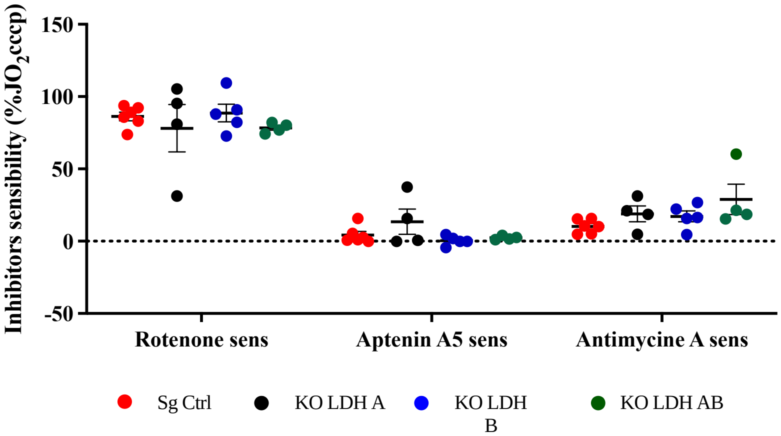

5. On a related note, it is unclear why the authors used LDH inhibitor and VEGF inhibitor in combination in Figure 6. Based on Figure 5, it seems targeting LDH in combination with radiation therapy is a good direction. It is necessary to provide why radiation and VEGF inhibition approaches were used in

combination with LDH inhibition. In addition, both radiation and VEGF inhibitor should be treated in combination with the LDH inhibitor stiripentol and compare which would be more beneficial treatment for GBM. If there are any additional drugs that are routinely used in GBM, these need to be included in the combination study. Especially because the combination of VEGF inhibitor and LDH inhibitor seems not beneficial at all in vivo.

Reply: VEGF inhibitor (bevacizumab) was used in Figure 6 to improve stiripentol accessibility, as observed in our previous study (Daubon et al., Nature Communications 2019) and when used on double LDHA/B KO in **suppl Figure 4h**, it improves survival. Bevacizumab was characterized to improve vessel quality and molecules penetration in the tumor, it appears that stiripentol follows the same trend that the one observed with anti-thrombospondin-1, as published earlier by our team. As replied to **Reviewer 1 (point 12)**, a new experiment will be performed by treating implanted animals with stiripentol and irradiation + temozolomide. As proposed to **Reviewer 1**, we can propose this experimental procedure to mimic Stupp protocol.

We can propose this experimental plan (as proposed to Reviewer 1):

- **Group 1** (8 mice) control treatment from Day 27, 3 times a week.
- **Group 2** (8 mice) Stiripentol 150 mg/ml from Day 27, 3 times a week.
- **Group 3** (8 mice) TMZ 25 mg/ml on Day 28 (AM + PM), Day 29 (AM + PM), Day 30 (AM) then X-ray 2 Gy Day 30 (PM), 2 Gy on Day 32 (PM) and 2 Gy on Day 35 (PM), then 1 day per week from Day 36 pf TMZ at 50 mg/ml.
- **Group 4** (8 mice) Stiripentol 150 mg/ml from Day 27, 3 times a week, then same treatment than group 3.

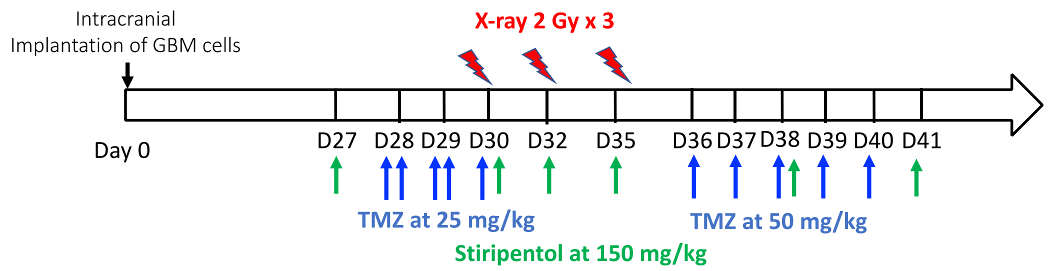

6. The authors state in their abstract that lactate is the metabolite that replenishes the TCA cycle, and this is based on the metabolomics data obtained using LDHA/B KO. How are the authors sure that all these changes including alteration of metabolites and metabolic gene expression (Figure 4c) are due to lactate but no other metabolites in cells lacking LDHA/B? Unless the authors demonstrate that replenishment of lactate restore these changes, it is hard to conclude that the driver was lactate.
Reply: We thank the reviewer for this important comment. We thought about this specific point and have previously generated results based on invasion experiments. We have treated double LDHA/B KO spheroid with lactate or HCl (to mimic acidic pH) and measure invasion rate. The same increase of double

LDHA/B KO cell invasion was observed when treating with lactate of HCl, which is much lower than the one observed on control spheroids, as depicted below:

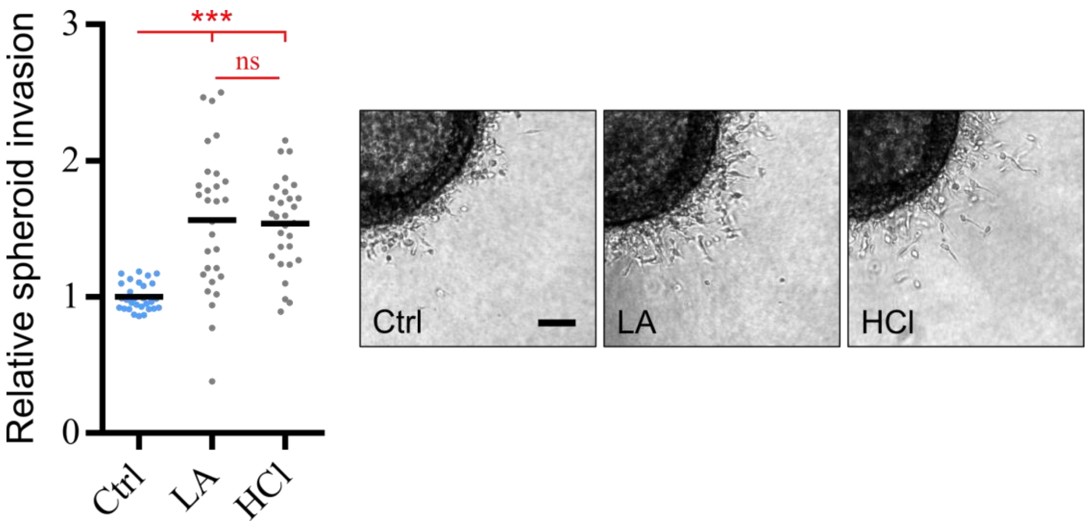

We can conclude that lactate cannot be used as a metabolite to sustain cell invasion of double LDHA/B KO, but acid pH triggers small increase in invasion rate (rate of 1.5 compared to a value of 3.8 when wt spheroids are stimulated with lactate, **Figure 2g**).

Reviewer #2 (Significance (Required)):

Please see above comments.

Reviewer #3 (Evidence, reproducibility and clarity (Required)):

Summary

The publication of Guyon et al reports that LDHA and LDHB contribute to the growth and invasion of glioblastoma stem-like cells by supporting the TCA cycle to maintain energy metabolism in hypoxic tumor areas. By creating single or double-knockout LDHA/B GBM cell lines as well as by metabolomics and transcriptional analyses, the authors identify changes of genes and metabolites involved in energy metabolism and report that lactate fuels oxidative phosphorylation in order to sustain tumor development. While LDHA and LDHB alone were shown to be insufficient therapeutic targets, a combined knockout of LDHA and LDHB significantly inhibited the malignant phenotype of patient-derived GBM stem-like cells. In addition, in vivo mouse studies with intracranial tumor injection with LDH activity inhibited by the antiepileptic drug stiripentol in combination with the antiangiogenic drug bevacizumab were

performed. Guyon et al conclude that the activity of LDHA and LDHB in GBM is a potential new target for glioblastoma therapy when combined with other drugs or irradiation.

Major comments

Point 1 : The introduction of the publication does not cover all relevant information. First, the authors only describe the two main LDH subtypes LDH-A and LDH-B and leave out the other two LDH subtypes LDH-C and LDH-Bx. Furthermore, in the human body LDH exists in five different isozymes composed of four subunits, i.e. LDH-1 to LDH-5 (Drent et al, 1996; DOI: 10.1183/09031936.96.09081736). Depending on the composition of the LDH isozyme available in the brain, more information can be drawn from the presented data. The publication of Bittar et al from 1995 for instance describes the dominant LDH isozymes in astrocytes and neurons (DOI: 10.1097/00004647-199611000-00001). Further information is necessary for an improved interpretation of the published data. In general, some information in the introduction is not cited at all.
Reply: We thank the reviewer for this comment and we completed our introduction based on these recommendations. Of note, LDH-C and LDH-D are detected at very low level, as reported in brain tissues (protein atlas database). Our results based on *in vivo* RNA sequencing previously published (Daubon et al, Nature Communications 2019) showed no expression of LDH-C and very low of LDH-D. LDH-Bx cannot be detected as there is only a slight difference with LDH-B of 7 supplement amino acids. Moreover, LDHC was characterized as mainly germinal, and LDH-D mainly metabolizing D-lactate.

Other passages could be removed since they are not important for the story and hence are confusing (i.e. the information about NMDAR is not required).
This sentence has been removed.

A reagent list cannot be found in the supplemental documents.
The reagent list can be found as supplemental file named: Reagent List.

Point 2: Figure 1 shows the comparison of a healthy mouse brain with human GBM stem-like cells. This comparison is not suitable because different species can express a different pattern of LDH isozymes. Furthermore, since the brain is one of the most complex organs, it is unclear which area of the brain was used as control and if there is a general difference in the LDH expression pattern in different brain areas. An age-dependent change of LDH expression should also be excluded by appropriate experiments.
Reply: As previously explained to **Reviewers 1 and 2**, staining procedure of LDHA and LDHB required extensive work in the P3 tumor model. Control areas were mainly taken in striatum of mice from 16 to 18 week-old. As shown in **Reviewer 1 (point 1)**, LDHA and LDHB expression are much higher in the tumor areas than in the control areas.
However, we consider that checking expression of LDHA and LDHB in the physiological brain is outside the scope of our manuscript.

Were the results confirmed with another GBM cell line, i.e. big cells?
To confirm our results obtained with P3 cells, we planned to implant another stem-like model, so-called BL13, in mouse brain to analyze the expression of LDHs. These results will be presented in the final

revised manuscript.

The age of the mice and the number of tested animals should be specified. Statistical analysis of biological replicates should be included.
This has been answered in this **Point 2** reply and specify in the text.

At present the text does not explain why the spheroid model was used in addition to the in vivo model. One may argue that in the in vivo model it is unclear whether the cells at the invading edge are tumor cells or healthy therefore, the spheroid model is important and shows that the peripheral tumor cells show a different LDH composition that the ones in the tumor core. This should be explained in the text.
We characterized in several previous studies the importance of using such spheroid model as a simplified avatar of tumor, as mentioned in the text "a model that recapitulates GBM oxygenation regional heterogeneity (ref29)".

To distinguish between tumor or healthy cells, we have performed an experiment in which GBM cells were expressing GFP and analyze the size of the nuclei which are bigger than nuclei from mouse microenvironment. Moreover, cells in the corpus callosum modify their shape for getting mesenchymal phenotype:

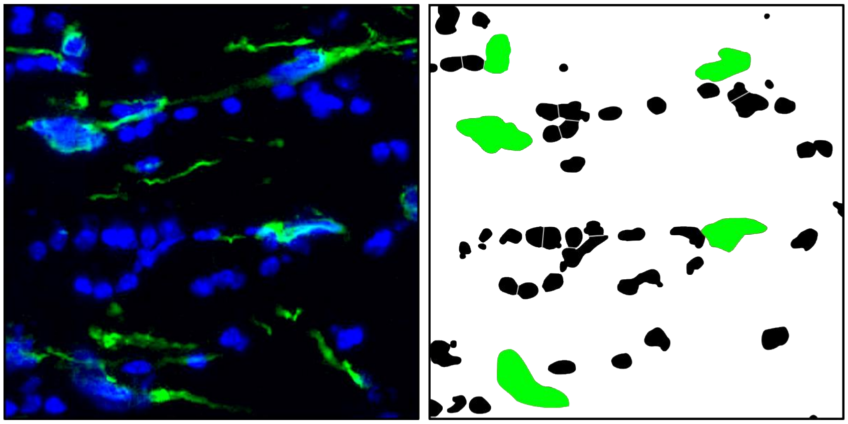

Another image below present GFP-P3 cells constituting tumor invasive area in which we can also observe than tumror cells (in green) have much bigger nuclei than cells from the microenvironment (non-GFP) :

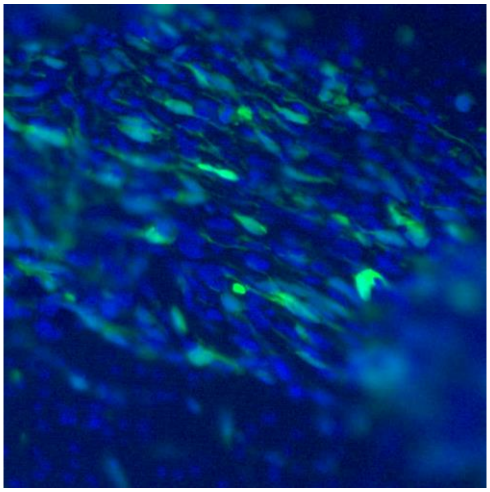

In general the text can be improved.
We thank the reviewer for this comment and we improved the description of figure 1 (sentences in red).

Point 3: In figure 2 only the activity of total LDH and of LDHB is shown, but information about the activity of LDHA is missing. Since other LDH subunits than only LDHB and LDHA could be present, the activity of LDHA should be included.
Reply: As replied to **Reviewer 2 (point 3)**, the LDHA activity will be assessed in the final revised version using an internal method we will developed as no commercial kit exists.

Point 4: In figure 3 it is unclear if the hypoxic condition was at 0.1 % O2 (3a and 3f) or at 1 % O2 (3b). In addition, the oxygen concentrations in the figure differs from the indicated values in the figure description. The hypoxic conditions varies in the publication from 0.1 % O2 to 1% O2 (figure 4) and can therefore not be compared. Any explanation of the different conditions is missing.
Reply: We apologize about these discrepancies. We used 0.1% hypoxia for all experiments in **figure 2**, as we performed assays on control spheroids. Then, in **figures 3 and 4**, 0.1% hypoxia was used for short experiment such as invasion assay or metabolomics experiments (maximum 2 days) and 1% for longer experiments such as proliferation assay (around 4 to 7 days). This was due to low viability of souble LDHA/B KO cells observed at 0.1% 02, as already demonstrated for 1% O2 in **figure 3d**. We thank the reviewer for pointing out that a mistake was inserted in the legend of figure 4, as the experiments were done at 0.1% O2. This is now corrected.

Presented western blot data should be quantified according to figure 2a.
Western-blots were quantified as shown here (related to tubulin):

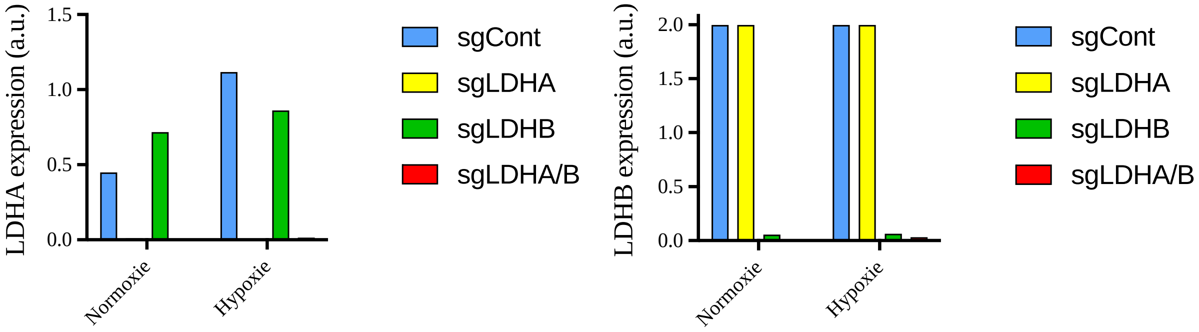

We decided to not include this quantification as it only confirms that our KO of LDHA and LDHB worked.

Point 5: Figure 3f is not required for a standard in vivo experiment, the method description is sufficient.
Reply: We removed this panel and respective legend as suggested by the **Reviewer**.

Point 6: It is striking that mice bearing LDHB knockout tumors show significantly lower survival than those with a knockout of LDHA and LDHA/LDHB (Fig 3i) but the tumor core and tumor invasion (Fig 3b) did not show differences. What is the reason for this observation? In the discussion the authors describe hemorrhages at the tumor site of mice bearing LDHB KO tumors. This should be supported by appropriate data and also dealt with in the results and more extensively in the discussion. Were hemorrhages also present in the double knockouts and if not, why not?
Reply: As shown in the figure below, we can detect that blood vessels from KO LDHB are larger than in the other tumors, such as control tumors. We are actually precisely quantifying blood vessel density and will bring more details in the final revised version of our manuscript. Of note, no hemorrhage was observed in double LDHA/B KO as no or low lactate was produced by the tumor cells, leading to low tumor acidosis. It has been characterized by Pierre Sonveaux group that lactate and acidosis lead to neo-angiogenesis (doi: 10.1158/0008-5472.CAN-10-2828 ; https://doi.org/10.1371/journal.pone.0033418)).

Below an example of blood vessel density (anti-CD31 staining, in red) in control and LDHB KO tumors :

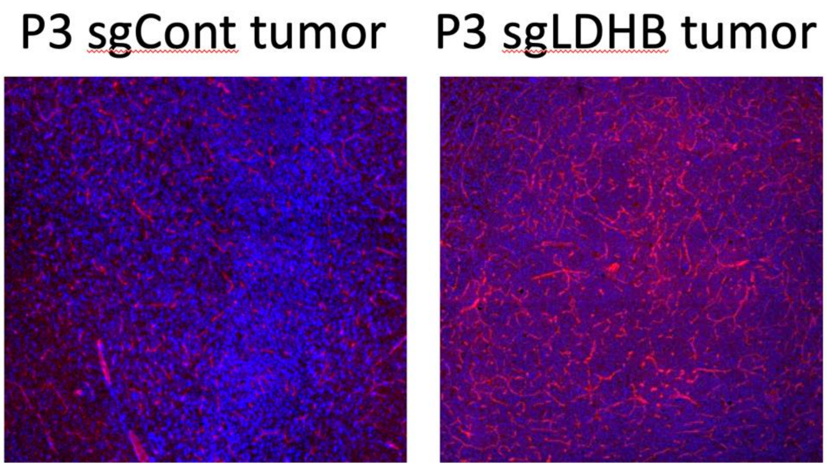

This point is also discussed in **Reviewer 1 Point 6** (precise blood vessel quantification included in final revised manuscript).
Discussion was also improved to better explain this point.

Point 7: The data shown in 3g and h raises various questions:
How is the tumor core defined? How do you differentiate between tumor core cells and invasive GBM cells? The indicated areas for the cell invasion, i.e. the red square in the histological images, do not cover the complete dark region (assumed as the invasive area), why did you exclude the rest? The quantification is unusual and unclear and therefore difficult to assess.
Reply: The implantation is proceeded into the striatum (red star) and tumor is growing by invading the corpus callosum as shown in the images below (white stars):

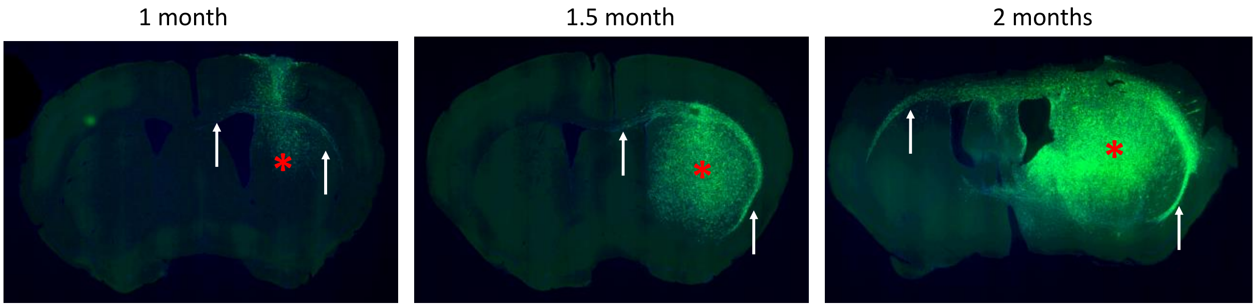

Then, tumor core is mainly defined as the area in the striatum and invasion is quantified in the corpus callosum, which are visible and easily quantified brain areas, as shown in the figure below:

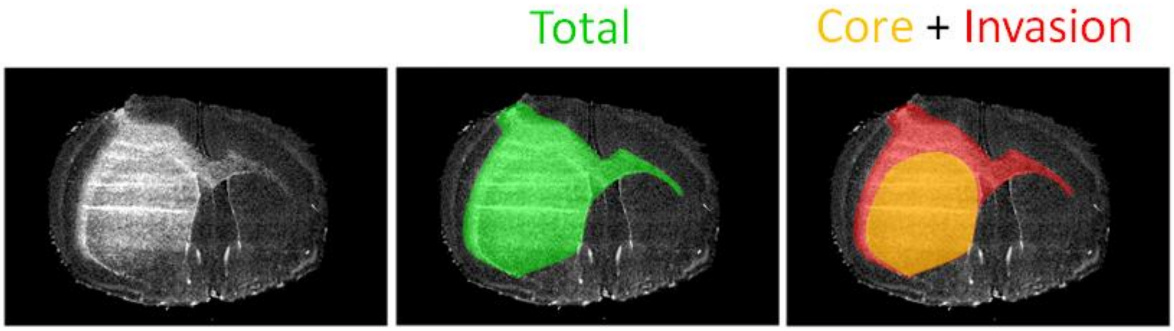

How old were the mice in g and h, were they all sacrificed at the same day after tumor injection? We had to perform two different *in vivo* experiments as control cells in **Figure 3g** only contain one CRISPR-cas9 plasmid and control cells in 3h contain both puromycin- and blasticidin resistant constructs. This control was first employed to verify if growing cells in both antibiotics would impact general proliferation, which was not the case, and this is why we could generate a second set of experiments (**Figure 3h** in the new Figure). The mice were of 16-week-old and 20-week-old in **Figure 3g** and **3h**, respectively.

The unit of the tumor core and the tumor invasion, meaning in relative to brain, is unusual and needs further explanation.
We have changed the graphs and applied new calculations for putting a unit to quantify core and invasive areas in **Figures 3 and** 6 (in mm^2).

Point 8: While 3g compares the single knockouts with the control, 3h only compare the double knockout with the control. How do single and double knockouts compare?
Reply: This has been commented in point 7, second reply.

Point 9: Figure 4 shows the metabolic and transcriptomic status of the created single and double-knockout LDHA/B cell lines and could be improved by including the metabolic and transcriptomic data of these cell lines under normoxic conditions, i.e. in a supplemental figure.
Reply: Our bioinformatician is generating the new figures and this will be ready within next week. We will propose these comparisons: Single LDHA or LDHB KO vs control at T0 and T48h (4 new metabolic pathways with RNAseq/metabolomics data).

Point 10: Phenformin inhibits the oxidative phosphorylation in cells thereby altering their energy metabolism. Since this effect impacts not only tumor cells but the whole body, please add additional information on the impact of phenformin in healthy individuals. The used concentration of phenformin in figure 5 is not explained in the paper. Was the applied concentration determined in an in vitro pretest?

Was it found in other publications? How can the authors conclude that the employed concentration was sufficient to inhibit oxidative phosphorylation? Further information are required.

Reply: Phenformin was used as complex I inhibitor, to link results obtained with rotenone in *in vitro* invasion assays or respirometry. Of note, phenformin was found efficient on control tumors but not on double LDHA/B KO (**Figure 5e**), suggesting that the concentration used in this experiment was inducing a good bioavailability of the drug, as suggested in several publications (Jiang et al, 2016 Oncotarget, doi: 10.18632/oncotarget.10919). This is now better explained in the discussion. The dose was then based on previous published protocols.

Point 11: In figure 6 the combined treatment of bevacizumab and stiripentol is shown. How efficient is the inhibition of the LDH activity in the cell lines by stiripentol? This could be easily investigated by a dose-dependent in vitro experiment.

Reply: We have performed an experiment by using 100 and 500 µM stiripentol concentration and invasion was recording in our collagen assay. As shown below, there is a dose-response which is observed by using either 100 or 500 µM stiripentol concentrations on invasion capacities but not on proliferation:

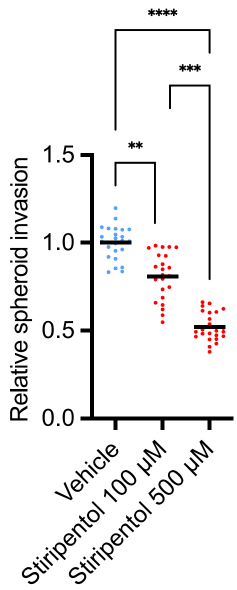

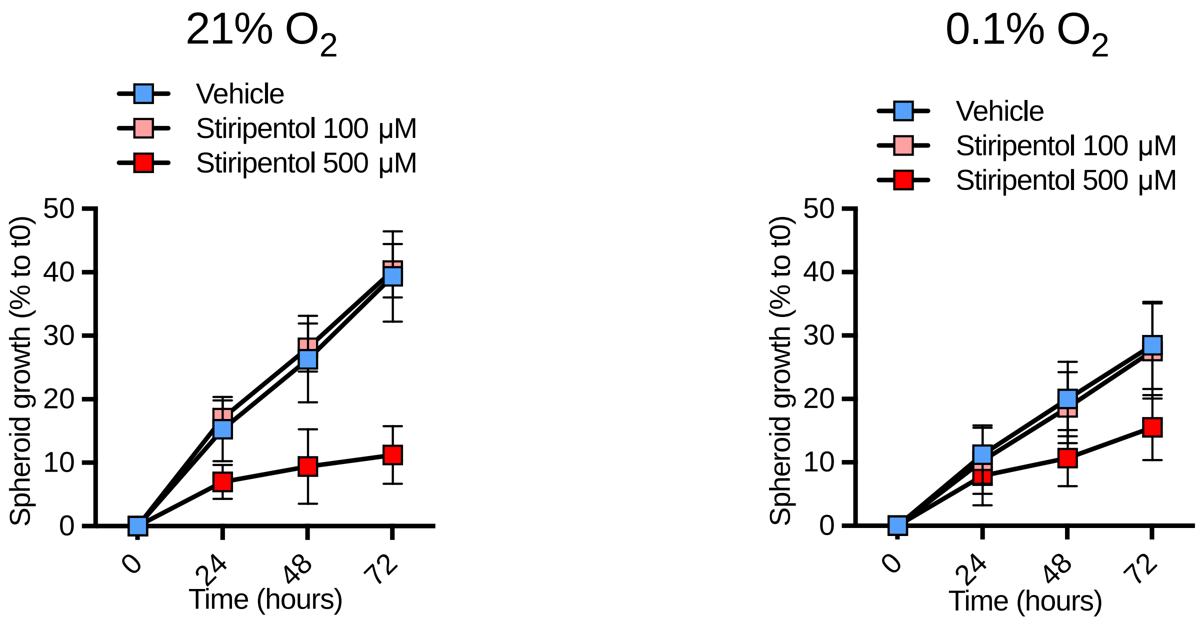

The concentration of 500 µM was used based on those first results.

Point 12: In order to determine the effect of the combined treatment, the combo needs to be statistically compared to both single treatments, not only to the vehicle. Figure 6f and the tumor core data of 6g show that mainly bevacizumab is important for a better survival of the animals. This has to be stated in the manuscript and also modified in the abstract.

Reply: We agree on the moderate effect of stiripentol on mouse survival, but we consider that the effect observed was sufficient to be included. We added statistical analysis between combo treatment and stiripentol alone, which gave a P value < 0.01.

Bevacizumab impacts tumor growth but we also interestingly found that combo treatment induced a strong decrease in contro-lateral invasion (Figure 6g, right graph) which is essential in future perspectives, as invasive cells are the roots of future recurrent tumors. We then believe that the results are of importance.

Point 13: As mentioned above, further information about the calculation of the tumor core and the tumor invasion is missing as well.

Reply: This is now explained in point 7 and modified in **Figures 3 and 6**.

Does the combination of LDHA/B knockout with bevacizumab show a combined effect?

The result was presented in **Suppl Figure 4h** (*** statistical differences, p<0.001).

Point 14: In general, in figure 1 it was shown that spheroids have a different LDH expression pattern at different regions. The comparison of injected spheroids displaying regional-dependent LDH expression patterns with the metabolic and transcriptional analysis of cultivated GBM cells in suspension could greatly differ in their metabolism and thereby not reflect the situation in the tissue.

Reply: We agree with the reviewer that *in vitro* and *in vivo* results could be different, obviously, but we

claim in the article than spheroid can efficiently recapitulate *in vivo* situation, as shown in our results. As an example, we found same results *in vitro* and *in vivo* results for double LDHA/B KO which inhibits spheroid and tumor growth and invasion. We do believe that this experimental procedure is a strength for neuro-oncology studies.

Minor comments

The text needs to be checked for grammatical errors. The experiments should be described in a way that they can be reproduced. References should be checked and included where they are missing, i.e. in the introduction. Hypoxic conditions should be made clear to avoid any confusion. In general, check if all necessary information for each figure is included in the figure and the figure description, do not exclusively insert important information in the main text.
Reply: We carefully corrected the manuscript.

Reviewer #3 (Significance (Required)):

The manuscript is of moderate novelty. The presentation of lactate accumulation and the increased activity of LDH under hypoxia is presented for the specific cancer type GBM. Nevertheless, most of the information is already known for most cancer types (i.e. Warburg effect). The main significance of this paper relies in the potential co-therapeutic targeting of LDHA and LDHB in GBM patients.
Reply: The reviewer considers our article as of moderate novelty but we can underline that we generated original results which, we believe, will be of importance for a broad audience. Our article does not rely only on Warburg effect, which is the adaptation of cells after hypoxia, but also rely on lactate symbiosis between hypoxic cells and oxygenated areas, as suggested in some previous work. Metabolic symbiosis is of great importance, as discussed into details in the review of Corbet and Feron ("Tumour acidosis: from the passenger to the driver's seat": doi:10.1038/nrc.2017.77). Here, we show that GBM cells cultured in hypoxia can fuel cells from oxygenated area by feeding them with lactate, to trigger cell invasion, in a symbiotic way. This is the first time that the role of both LDHA and LDHB is described into details in glioblastoma. We also discuss in great details how LDHA/B KO cells, meaning without lactic fermentation, adapt in severe hypoxia. This is the first time that regulation of respiratory chain complex expression is shown in such a context.

My expertise
Long-standing experience working on amino acid metabolism in glioblastoma.

Revision Plan

3. Description of the revisions that have already been incorporated in the transferred manuscript

We have already incorporated several changes in the transferred manuscript, as listed below:

- Numerous modifications were included in the main text and in legends for Figures 1, 3, 4, 5 and 6.
- Quantifications of images in Figure 1.
- Addition of survival curves in suppl. Figure 1.
- Precisions on impact of LDHB KO have been brought.
- Introduction was improved (reviewer 3).

4. Description of analyses that authors prefer not to carry out

1st Editorial Decision **2nd Nov 2021**

Dear Dr. Daubon,

Thank you for the submission of your research manuscript to our editorial offices. I have now had the opportunity to read your manuscript, as well as the referees' reports and your rebuttal letter, and to discuss them with the other members of our editorial team. We appreciate that you are willing to address/have addressed the points raised by the referees, however I am afraid we do not think the manuscript is well suited for publication in EMBO Molecular Medicine and have therefore decided not to proceed with peer review.

Indeed, we appreciate that your study investigates LDHA/B contribution to glioblastoma progression and invasion, and that you propose a new therapeutic strategy based on the use of the anti-epileptic drug stiripentol combined with the anti-angiogenic bevacizumab.
However, we are not convinced that the translational advance is sufficient for publication in EMBO Molecular Medicine, as the strong anti-tumor effects observed might be due to bevacizumab. We realize that you are proposing to address this by performing further experiments, but the issue of these experiments remain uncertain at this stage. Furthermore, the new therapeutic strategy is not placed in the context of current standard of care.

I further consulted with referee #1 who reviewed your initial manuscript. This referee stated:
"I read the authors' rebuttal letter carefully, in particular their responses to our comments on the moderate to minor anti-tumor effects of targeting LDHs by the drug Stritipentol. While the authors' efforts seem excellent, their plan for improving the effects as illustrated in response #12 for Figure 6 may or may not work. In addition, the rational of combination treatment of Stritipentol with TMZ and RT is not stated in the rebuttal. Information of the GBM PDX models were not provided. In this case, the authors should use known TMZ-resistant and/or RT-resistant GBM PDX models and the rational should be that Stritipentol inhibition of LDHs should reduce TMZ- or RT resistance of GBM tumors in orthotopic brain GBM tumor xenograft models. This set of experiments has risk of not improved anti-tumor effects and using inappropriate GBM models."

Based on these considerations, I am afraid we cannot offer further consideration to your article here.

Please rest assured that this is not a judgment of the quality or interest of your work, but a decision based on the scope requirement of our journal.

Yours sincerely,

Lise Roth

Lise Roth, Ph.D
Editor
EMBO Molecular Medicine

****
Rev_Com_number: RC-2021-00982
New_manu_number: EMM-2021-15343
Corr_author: Daubon
Title: Specific expression of lactate dehydrogenases in glioblastoma controls intercellular lactate transfer to promote tumor growth and invasion

Dear Dr Roth,

Thank you for your message.

I would like to discuss with you about your message because I believe that there is some points to comment (e.g. "Furthermore, the new therapeutic strategy is not placed in the context of current standard of care" = we designed the new experiment on Stupp protocol, which is the only protocol used for GBM patients ; "the issue of these experiments remain uncertain at this stage" = this is true but this is the case in all experiments asked during the revision process, and we believed in the potential of this drug ; from Reviewer "MZ-resistant and/or RT-resistant GBM PDX models" = we can consider that all our models are resistant as the animal unconditionally do not survive when implanted with our stem-like GBM cells).

Then, as a young group leader, I would need your advice to see if this is a good strategy to continue submitting to EMBO journals.

Please, let me know if this is possible for you.

Thank you for your help.

Best regards
Thomas Daubon

Dear Dr. Daubon,

Thank you for your message regarding your recent submission to EMBO Molecular Medicine.
To address your points, should you perform the in vivo experiments convincingly showing the efficacy of the treatment independently from bevacizumab, we would welcome a resubmission to EMM. Please be aware that I cannot guarantee the outcome of a resubmission.
If you would like to discuss further, we could schedule a zoom meeting at your convenience.

I hope this helps,

With my best wishes,

Lise Roth

Lise Roth, PhD
Editor
EMBO Molecular Medicine
l.roth@embomolmed.org

Revision Plan

Manuscript number: RC-2021-00982 → EMM-2021-15343

Corresponding author(s): Thomas, Daubon

1. General Statements

Our article was globally appreciated by the 3 reviewers and we believe that explanations given in part 2 of the Revision Plan will convince the reviewers that our story is of importance for a broad scientific audience. We would like to thank the reviewers for the positive comments about our manuscript and we would like to bring more information about the significance and novelty of our work. Reviewer 1 said that the role of LDH in GB growth regulation was well studied but only few studies related to this topic exist: Seliger et al 2013 and Kim et al 2015. More importantly, lactate as metabolite has never been studied in such a great detail in GB development and especially in GB invasion.
Importantly, our article shows that not only on Warburg effect is occurring (as raised by reviewer 3), but that GB development relies on lactate symbiosis between hypoxic and oxygenated cells. Metabolic symbiosis is of great importance, as discussed by several scientific teams (*e.g.* important review of Corbet and Feron "Tumour acidosis: from the passenger to the driver's seat" doi:10.1038/nrc.2017.77). We added recent and important data from our collaborators and new co-authors (Kevin Joseph, Vidhya M. Ravi and Dieter Henrik Heiland ; "Spatially resolved multi-omics deciphers bidirectional tumor-host interdependence in glioblastoma" Ravi et al., Cancer Cell, Jun 2022) in which we can observe a regional expression of LDHA and LDHB in patient samples has been observed compatible with a lactate-dependent metabolic symbiosis in patient samples. Here, we show that GB cells cultured in hypoxia can fuel cells from oxygenated area by feeding them with lactate, to trigger cell invasion, in a symbiotic way. This is the first time that the role of both LDHA and LDHB is described in such a great detail in GB including RNA sequencing coupled with ^{13}C metabolomics. We also discuss extensively how LDHA/B KO cells adapt to severe hypoxia. This is the first time that regulation of respiratory chain complex expression is shown in that context. Moreover, we repurposed the antiepileptic drug stiripentol and validate the drug in a preclinical orthotopic GB model. A massive increase in survival was observed when compared to the control group.

We have now fully revised our manuscript and answered to all reviewer's questions.

A detailed response to the reviewer's comments is given below.

Revision Plan

We can propose a graphical abstract for our revised manuscript:

Legend: This study highlights the importance of metabolic symbiosis dependent on lactate and lactate dehydrogenases isoforms (LDHA and B) in glioblastoma development.
Targeting both lactate dehydrogenases may be a novel potential therapeutic approach for targeting glioblastoma.

- Lactate, which is produced in hypoxic environments, is secreted and uptaken by oxidative cells to full the Krebs cycle to promote growth and invasion.
- Only double knockout of LDHA/B abolished lactate production, reduced tumor growth and invasion, and finally prolonged mouse survival.
- Tumors that no longer express LDH are more oxidative and more sensitive to radiation.
- Use of the LDH inhibitor stiripentol in the clinical practice may be therapeutically relevant for glioblastoma.

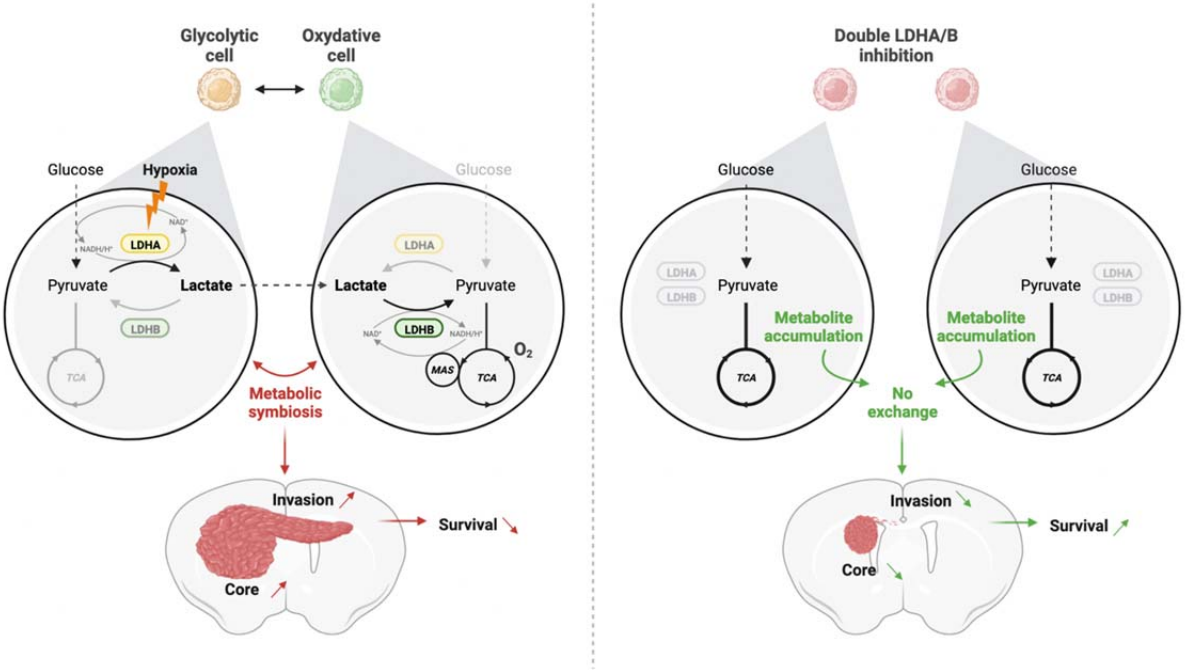

2. Description of the planned revisions

The responses to reviewers were written in blue in the text.

Reviewer #1 (Evidence, reproducibility and clarity (Required)):

In this manuscript by Guyon J. et al., the authors have demonstrated that in GB the two isoforms of the enzyme Lactate Dehydrogenase (LDH), namely LDHA and LDHB, show preferential expression of LDHA in the tumor core while that of LDHB in the periphery using spheroid models and scRNAseq patient data. In P3 and BL13 stem-like models they have shown that high LDHA/B levels lead to high lactate secretion from the cells under hypoxic conditions which is responsible for spheroid growth and invasiveness by fueling the TCA cycle as an energy source, as shown by metabolic tracing experiments. Further, the authors show that double KO of LDHA and LDHA but not single KO of either A or B is responsible for reducing invasive tumor growth in mice as shown by orthotopic tumor experiments in mice. Moreover, by integrating metabolomic data generated by carbon tracing experiments and transcriptomic data generated by RNA Sequencing, the authors show that LDHA/B KO cells under hypoxic conditions, triggers a metabolic switch leading to upregulation of TCA and glycolysis related metabolites and transcripts. Components of the mitochondrial respiratory chain which were specifically upregulated were confirmed by Western Blotting and that inhibiting the high respiratory capacity by irradiation increases survival in mice. Lastly, they have shown that an LDH inhibitor could marginally increase survival in mice which is increased significantly with a combinational treatment with antiangiogenic treatment, bevacizumab.
This is an interesting study showing the role of LDHs in tumor progression using high throughput data analysis, state of the art biochemical techniques and other molecular biology tools. Although the role of LDHs in regulate tumor progression in GB are well known, the mechanistic pathways studied using global metabolomics and transcriptomic analysis is interesting and provides useful datasets in the field. Also, though the study is thoroughly conducted and data is mostly well presented, some of the data needs to be addressed further, most importantly regarding the mechanistic role of lactate through the respiratory chain complex as inhibiting the pathway did not affect survival in mice, and also, how does the anti-tumorigenic role of LDHB relate to the tumorigenic function of the double KO. Based on some of the issues mentioned below, the overall enthusiasm for publishing this manuscript in the EMBO journal in (is) medium.

Reply: We thank the reviewer for the positive comments and time spent to evaluate our manuscript and we would like to bring more information about the significance and novelty of our work. Reviewer suggested that the role of LDH in GB growth regulation was well studied but, to our knowledge, only few studies were related to this topic: Seliger *et al*., 2013 (siRNA of LDHA or LDHB but not both simultaneously and only in *in vitro* assays), Daniele *et al*., 2015 (siRNA LDHA and *in vitro* assays) and Kim *et al* ., 2015 (no LDH modulation in cell line and only *in vitro* assays). More importantly, lactate as metabolite has never been studied in such

great details in GB development and especially in GB invasion, by using double LDHA/B KO and a drug targeting both isoform activities *in vivo*. We have now answered all questions raised by the reviewer, performed crucial experiments, and thus manuscript has been improved.

Reviewer #1 (Significance (Required)):

Comments on both aspects:

1. In Figure 1, the authors should elaborate on their results better. For example, does the corpus callosum region highlighted in fig 1a portray the invasive region of the tumor as it is covered by the tumor periphery. Are both LDHA and LDHB expressed in this region? If yes, how is this region different from the periphery region which predominantly expresses LDHB.

Reply: We thank the reviewer for these comments and we have improved the explanations of this figure in the text.
The corpus callosum region is part of the invasive area, as depicted in the image below with the red line:

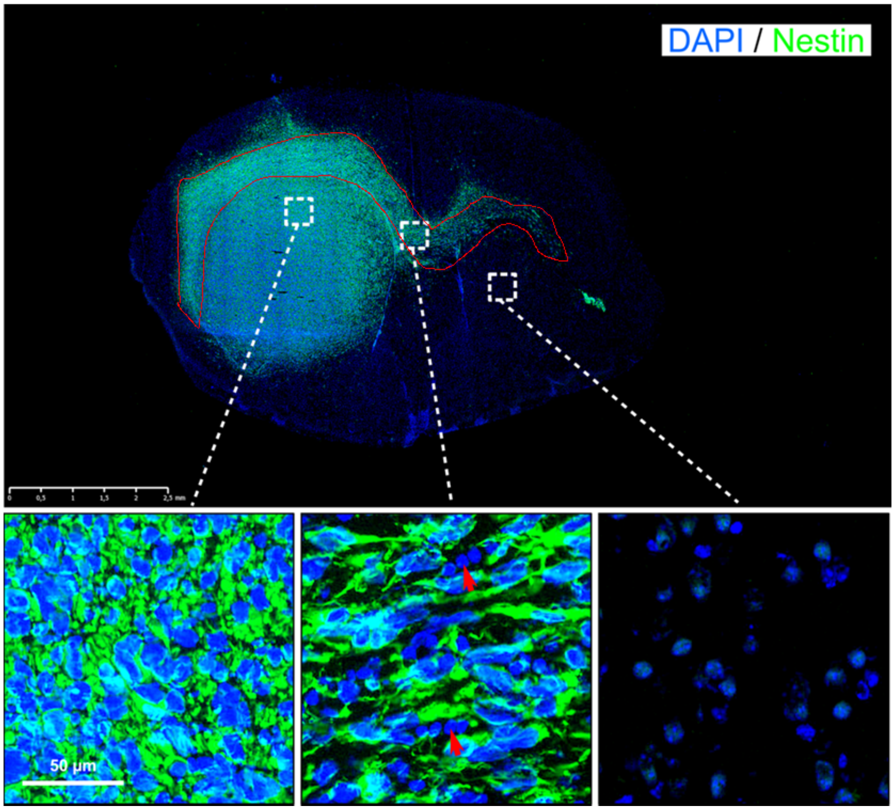

This area in the corpus callosum differs from the periphery area by the microenvironment, mainly composed of myelinated axons and giving access to the contro-lateral brain. Of note, GB cells are influenced by this microenvironment and acquire elongated/invasive phenotype (lower middle image). Both LDHA and LDHB are expressed in this region but as now better indicated

in the text and in the legend, some cells only express one or the other (as shown by the dotted lines in **Fig 1c**) and in the image below:

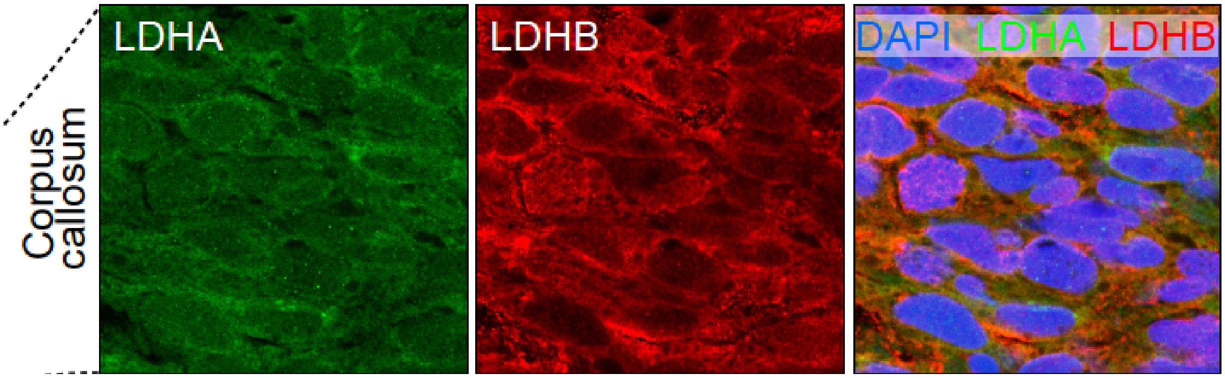

All the images were quantified in **Fig 1A** and **Supplementary Fig 1A**.

In **Fig 1D-E**, analysis from new co-authors (Kevin Joseph, Vidhya M. Ravi and Dieter Henrik Heiland ; "Spatially resolved multi-omics deciphers bidirectional tumor-host interdependence in glioblastoma" Ravi et al., Cancer Cell, Jun 2022) clearly shows exclusion of LDHB expression in hypoxic areas but high expression in peripheral/invasive areas. LDHA is highly expressed in hypoxic areas and in some invasive cells. These new results strongly reinforce our previously acquired results in the P3 model.

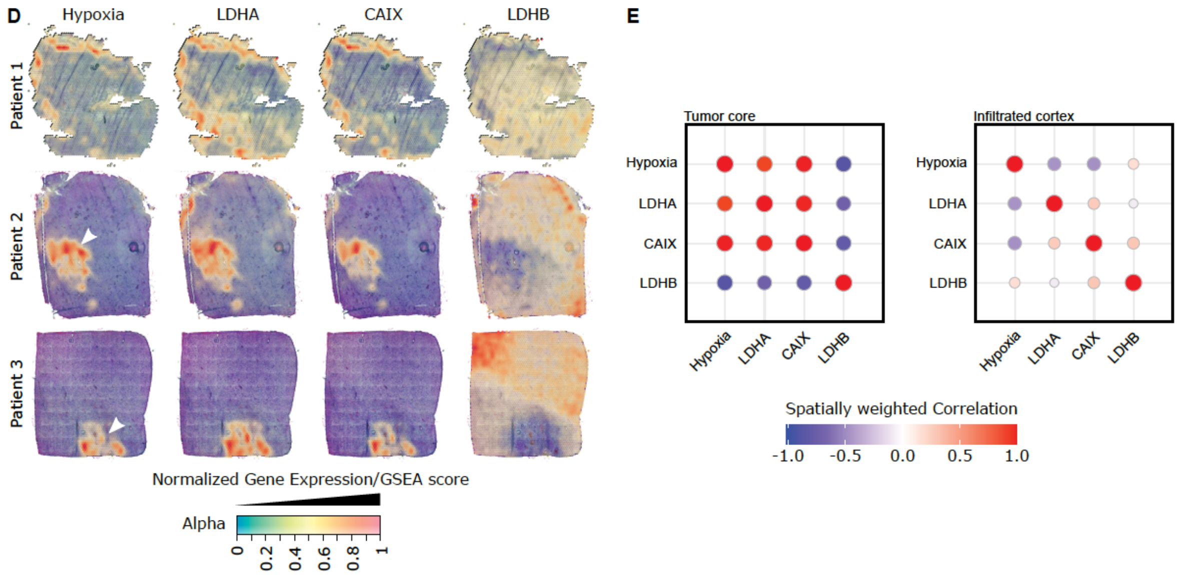

What does the dotted line indicate? Please mention in the legend.
This has been removed from **Fig 1**, as we quantified the intensities of LDHA and LDHB (see above).

Also, in fig. s1b, please explain how LDHA and B expression is correlated to anatomical origin of tumor and which regions represent the core and peripheral regions? Is the positive correlation between LDHA and HIFA observed in any particular anatomic locations in this analysis?

In **Supplmenterary Fig 2B**, LDHA is more expressed in infiltrative area (IT) and in cellular tumor with microvascular proliferation (CT mvp), corresponding to areas we defined as invasion area and to some hypoxic/central area, respectively. Globally, LDHA is more expressed in samples with high HIF1A expression as described. Of note, hypoxic areas cannot be sequenced by RNA sequencing, due to low RNA quality. Then, LDHB has no specific expression in the IVYGAP database but is globally less expressed in areas with high HIF1A expression. This has been better explained in the main text.

2. The survival curves in fig. s1c are only marginally significant. The authors should check for survival analysis in other publically available GB datasets as well.

Reply: We thank the reviewer for this comment and we generated other survival curves based on alternative database (CCGA). Please find it below:

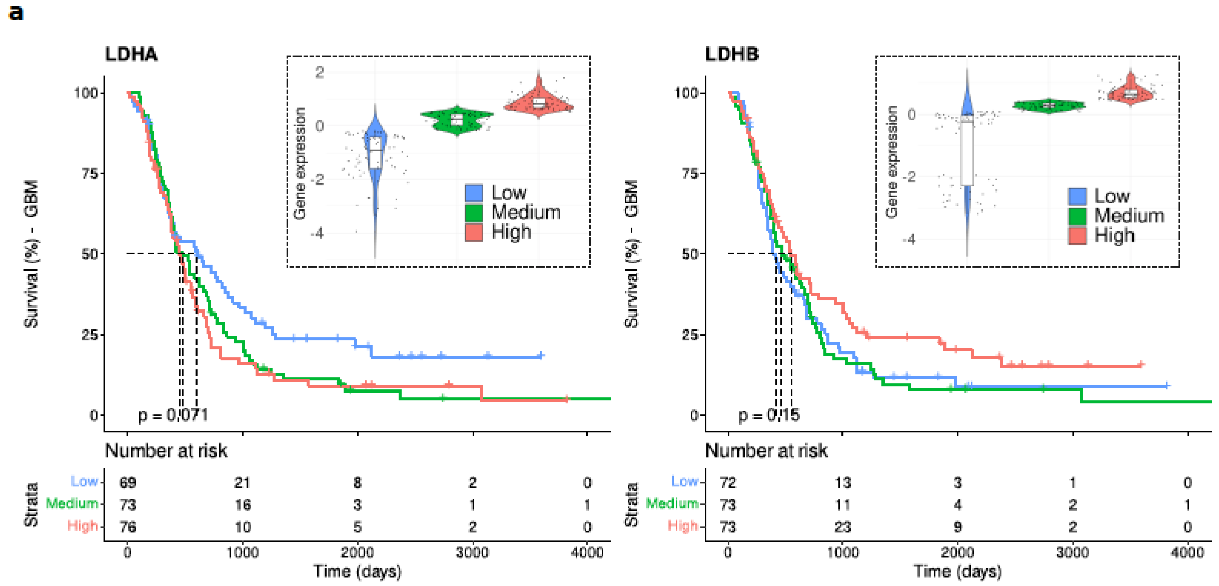

The same tendency was observed in CCGA and TCGA but we preferred not including in the revised manuscript.

Of note, in most survival curves for GB patients (as opposed to low-grade IDH-mutant glioma) the differences in survival are not large because of the overall dramatic evolution of the disease. For comparison, we generated graphs with survival curves for low-grade IDH-mutant glioma, and the same trend was found for LDHA and LDHB (LDHA as poor and LDHB as good prognostic factor). This was added in **Supplementary Fig 2C**.

3. In the metabolic tracing experiment, the amount of labelled lactate and pyruvate reached maximum levels between 0 to 5hrs showing that almost all of the lactate and pyruvate molecules in the cells become labelled within this time (Fig. S3b). However, in Figure S3c, the incorporation of labelled carbon does not appear to be very high within 0 to 5hrs as compared to citrate, which shows early incorporation in fig. s3c while achieves maximum activity in the exometabolome late around 24 hrs. What is the incorporation rate in citrate in the

endometabolome? Does it match with its maximum level time point in fig s3b? Please comment on this discrepancy.

Reply: We would like to recapitulate how the analysis was done in this experiment.
The fractional contribution represents the contribution of ^{13}C atoms to all of the atoms in a specific metabolite. This implies that in the case of citrate, if we - in theory- could only label a maximum of 2 molecules out of the 6 molecules, the fractional contribution will "penalize" the actual contribution, hence a lower labeling score than it actually is, is measured.
Looking at the isotopologues, we could have for instance 100% labeling in the m2 of citrate, but the fractional contribution will only be of 33%. Corrected isotopologues represent the actual "artificial" ^{13}C labeling. For small molecules like pyruvate, the chances are high that all carbons are labelled and then the formula of fractional contribution works, while larger molecules have carbons that cannot be labeled from specific sources, hence they have a lower labeling percentage. The incorporation rate of citrate in fig s3c corresponds to fractional contribution of citrate in fig s3b.
Either we show the fractional contribution, or we show the isotopologues or a combination of both to make the point. Bart Guesquiere's team (Metabolomics facility, Leuven, Belgium) is working on an improved formula for fractional contribution, but but this is depended on the tracer used.
Of note, to transform abundance to quantity (based on VIB metabolomics expertise): metabolite abundances cannot be compared with each other, this is due to the difference in the ionization potential of each molecule. However, we can compare the abundance of the same metabolite across different conditions, this gives a relative abundance. Furthermore, those techniques are really expensive and time-consuming. We therefore believe that our method is highly suitable in the context of our project.
The figure below will make the analysis clearer.

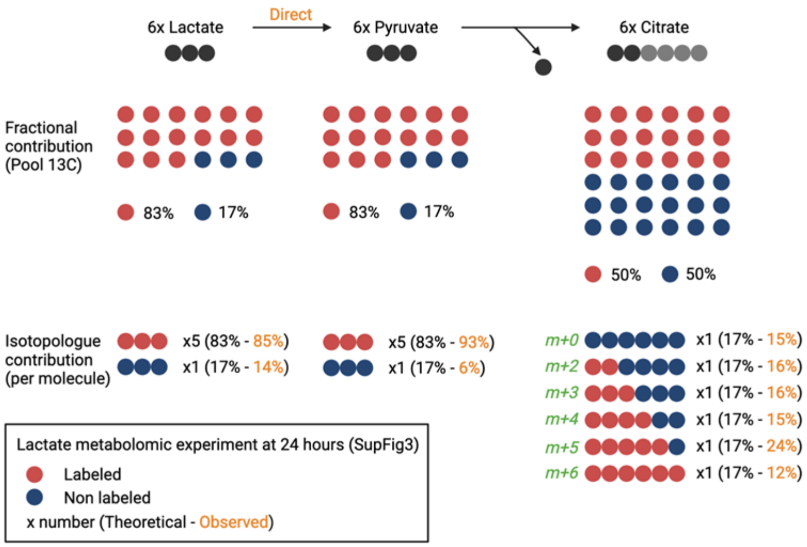

What do the values m+0 to m+6 indicate? Please specify in the figure legend.

→ m+0 stands for the fraction of metabolite without ^{13}Carbon and m+n (n > 0) stands for fraction of metabolite with n ^{13}Carbon. For example, m+5 correspond to a metabolite with 5 labeled ^{13}Carbon. The sum of (m+0, m+1,…, m+10,…) equals to 1.
This has been added in the legends.

4. The data is figure 2i is interesting where the authors show that lactate is used as an energy source via TCA cycle through metabolic tracing experiments. Here, the authors should also inhibit one of the crucial components of the TCA cycle such as malate produced by the malate aspartate shuttle, which appears to have a high rate of labelled carbon incorporation, and then measure the labeled glutamine levels in the exometabolome.

Reply: We thank the reviewer for this interesting suggestion. To our knowledge thanks to our research team members who are experts on it (Molinié et al, 2021, doi.org/10.1016/j.bbabio.2022.148532), inhibiting malate-aspartate shuttle (MAS) by using MDH inhibitors is not working. Another inhibitor massively used for inhibiting the MAS, *via* GOT inhibition, is aminooxiacetate (AOA) but again, this is unspecific and inhibits all transaminases which will directly impair glutamate and then glutamine production (we provided a figure with detailed malate-aspartate shuttle and pathways directly linked to glutamine to illustrate our reply).

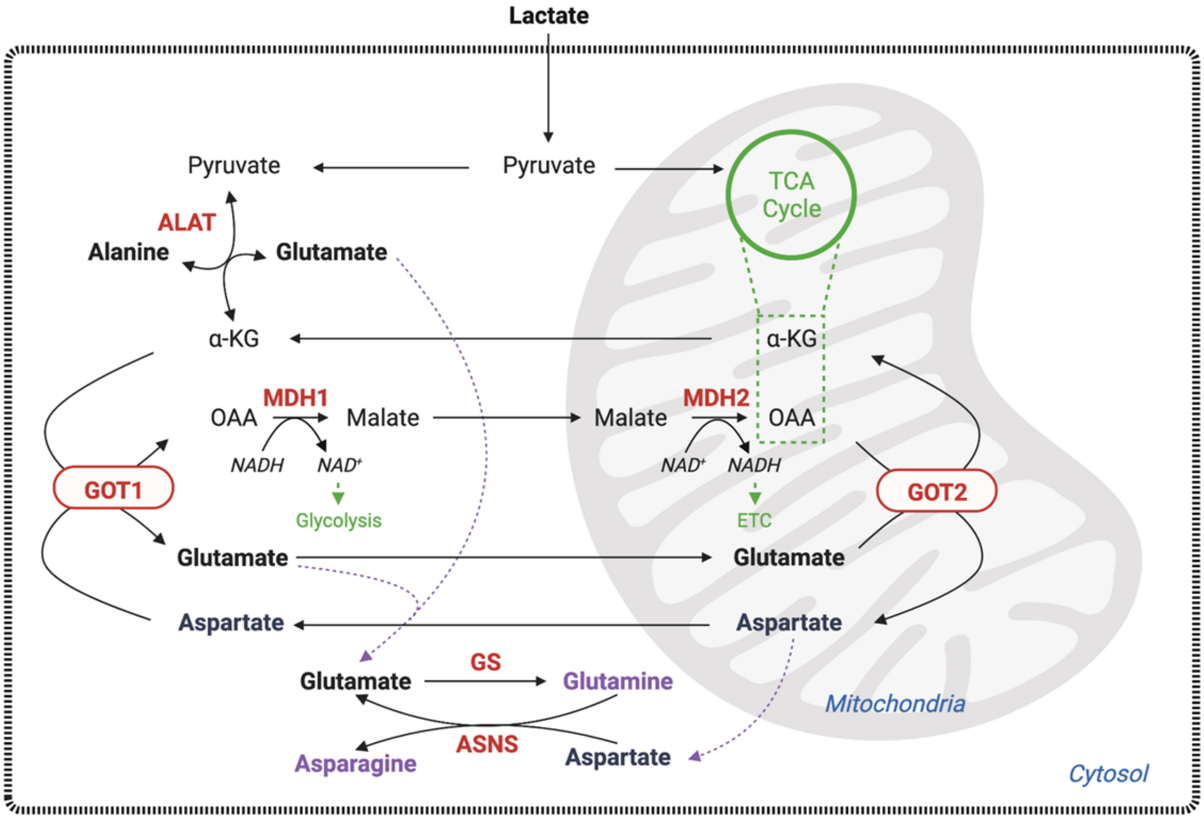

We performed similar metabolomic experiment than in the **Fig 2I** using cycloserine, which was characterized as a malate-aspartate inhibitor (https://www.jbc.org/article/S0021-9258(18)94588-6/fulltext). The major effect of cycloserine was to block the conversion of pyruvate to alanine, which is demonstrated by the absence of M+3 carbon for alanine. However, it is interesting to

note that the abundance of aspartate, glutamate and glutamine was reduced in presence of cycloserine after 4 hours treatment, with also a slight decrease in the enrichment of malate (**see Figure below**). The levels of labeled glutamine in the exometabolome did not appear to be affected after 24 hours of cycloserine treatment. Glutamate and glutamine are involved in many transamination reactions, including those catalyzed by alanine transaminase and GOT. The non-fully inhibited pathways could have been compensated by other metabolic mechanisms.

Supplementary Figure 5

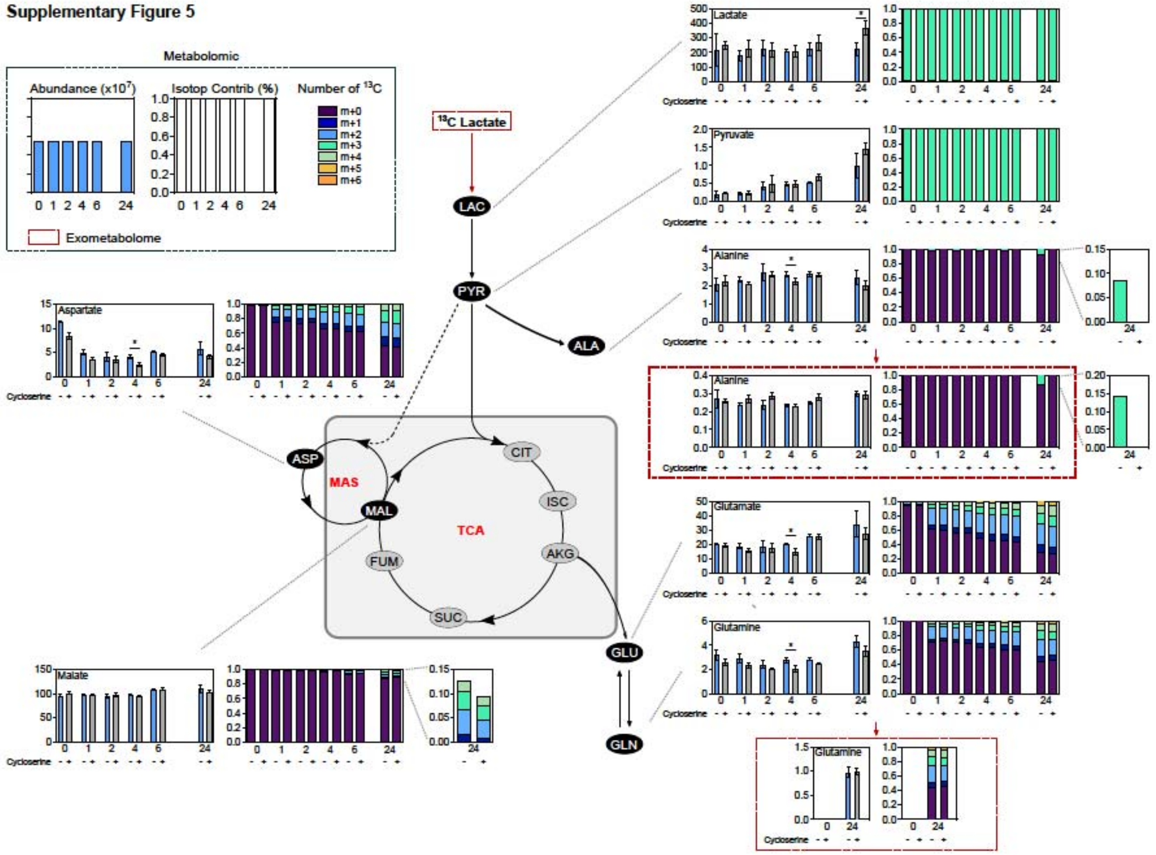

5. In Fig. 3e and S4f, how do the authors explain the higher invasives in P3 cells upon LDHB KO? Does KO of LDHB shuttles the lactate production to LDHA which causes increase in invasiveness? However, in Fig. 3c, KO of LDHB shows low lactate production. Please comment.

Reply: We thank the reviewer for this comment. P3 sgLDHB cells only express LDHA and this enzyme is related to poor patient prognosis (**Supplementary Fig 2C**). LDHA has a greater affinity for pyruvate than lactate and converts it into lactate to regenerate the redox potential, allowing the maintenance of the glycolytic flux. Indeed, the intracellular lactate production rate in **Fig 3C** is slightly lower for LDHB KO cells compared to control cells (0.05 min^{-1} *vs* 0.06 min^{-}

[1], respectively) and in this experiment, cells were stationary, did not invade any matrix, experiment followed cells during 35 minutes and used different agents (oxamate, diclofenac). However, in **Fig 3B**, lactate secretion in the extracellular compartment after 24 hours is higher for LDHB KO cells compared to control cells ($1.4x10^6$ RLU *vs* $1.1x10^6$ RLU). Then, lactate accumulation in the extracellular compartment is a pro-invasive metabolite, as shown in our article. This has been better explained in the text.

6. In Fig. 3i, the authors state that reduced survival in case of LDHB KO is likely due to hemorrhage at the tumor site, however in Fig. S1c, patient survival data, there is a similar pattern of worse survival in case of low LDHB expression. Also, LDHB KO increases invasiveness in Fig. 3. Also, VEGF increase only in the LDHB KO tumor (Fig. S4g) could also be due to the anti-tumorigenic activity of LDHB and not just hemorrhage. Overall, LDHB appears to have an anti-tumorigenic function. However, double KO of LDHA and LDHB is increases survival indicating their cumulative action to be pro-tumorigenic. Though the doble KO data is convincing, single KO of LDHB does not add to the hypothesis. Please comment on the above.

Reply: We think that the *in vivo* results of LDHB are of high importance. As the reviewer commented, LDHB appears to be anti-tumoral in patient survival data, and the results we obtained are in line with this finding. We showed that inhibiting LDHB expression leads to accumulation of extracellular lactate and protons (**Fig 3B**), inducing activation of many pathways *via* growth factors (TGFβ, VEGF), and an increase in GB aggressiveness, as mentioned in several papers from Pierre Sonveaux group (doi: 10.1158/0008-5472.CAN-10-2828; https://doi.org/10.1371/journal.pone.0033418). Thus, accumulation of extracellular lactate in LDHB KO cells leads to local acidosis which activates VEGF, as shown in **Supplementary Figure 6H**. VEGF is one of the most efficient growth factors to induce neoangiogenesis and in high concentration, it induces blood vessel leakiness, leading to hemorrhage. In addition, we quantified the vascular density in mouse brain slices (anti-CD31 staining) that shows increased vascular coverage of LDHB KO cells compared to control, LDHA KO and double LDHA/B KO (**Supplementary Figure 6I** and below). To conclude, LDHB cannot be considered as a therapeutic target by itself but only by complementing with LDHA inhibition.

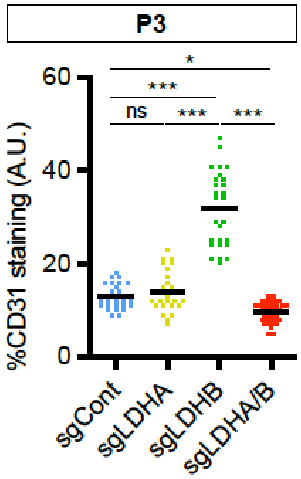

The text has been modified in the discussion section for more information. This reply is linked to the reply from **Reviewer 3 point 6** in which blood vessel quantification was requested.

7. In Fig. 3g,h, the authors should check the LDHA and LDHB levels in the tumor core and periphery to validate their localization in the tumor mouse model.
Reply: We thank the reviewer for this comment. In **Fig 3G and H** (now **3F** and **3G**), we implanted only GB control, KO LDHA, KO LDHB or double KO LDHA/B spheroids (only one cell line per brain). Thus, sgCont tumor expressed both LDHA and B (similar to the **Fig 1A**), the whole tumor sgLDHA expressed only LDHB, the whole tumor sgLDHB expressed only LDHA and the tumor sgLDHA/B did not express LDHs. To reinforce this finding, we generated a zymography experiment, sgCont cells presented the 3 heterotetramers composed of the possible LDHA and LDHB subunits (1A/3B – 2A/2B – 3A/1B) while sgLDHA and sgLDHB cells presented only the homotetramer of LDHA or LDHB subunits, respectively. Finally, double KO showed no homo or heterotetramer.

Figure for reviewers removed

8. In 4c left panel, control cells, the majority of transcripts related to glycolysis increased whereas the majority of metabolites related to glycolysis decreased. Why isn't there a similar pattern between metabolome and transcriptome? Although a similar pattern is observed in the middle and right panels.
Reply: A metabologram is a simplified visualization tool to view simultaneously transcriptomic and metabolomic data of compared conditions. Control cells under hypoxia (left metabologram) efficiently use glycolysis, leading to upregulation of most of enzymes linked to HIF1α (HKs, PFK1, ALDO, phosphoglycerate kinase, ENO1, PKM2 and LDHA) but no accumulation of metabolites, reflecting high glycolytic flux. The two other conditions (middle and right metabolograms) are related to comparisons with LDHA/B KO which leads to redox imbalance and then metabolite accumulation, as LDH cannot regenerate NAD+.

9. In Figure 5, although the components of the respiratory chain were validated to be high, inhibiting the respiratory complex I did not decrease tumor formation. Did the authors try to use inhibitors against the glycolytic pathways which are also seen to be upregulated in LDHA/B KO in Figure 4.

Reply: This is an interesting point.
We have performed this experiment by treating control and LDHA/B KO spheroids with 2-DG which bypasses the glycolysis, either in normoxia or hypoxia. 2DG evenly induced toxicity in both cell types in normoxia, as cells can use mitochondrial respiration based on other sources (lipids, glutamate). However, in hypoxia, sgCont cells were more sensitive to 2-DG than sgLDHA/B cells, even if double LDHA/B KO cells had a higher death rate than without 2-DG, as previously shown in **Fig 3D**. This strongly demonstrates the high metabolic plasticity of GB stem-like cells.
These results were inserted as **Supplementary Fig 12** and commented in the text, and below:

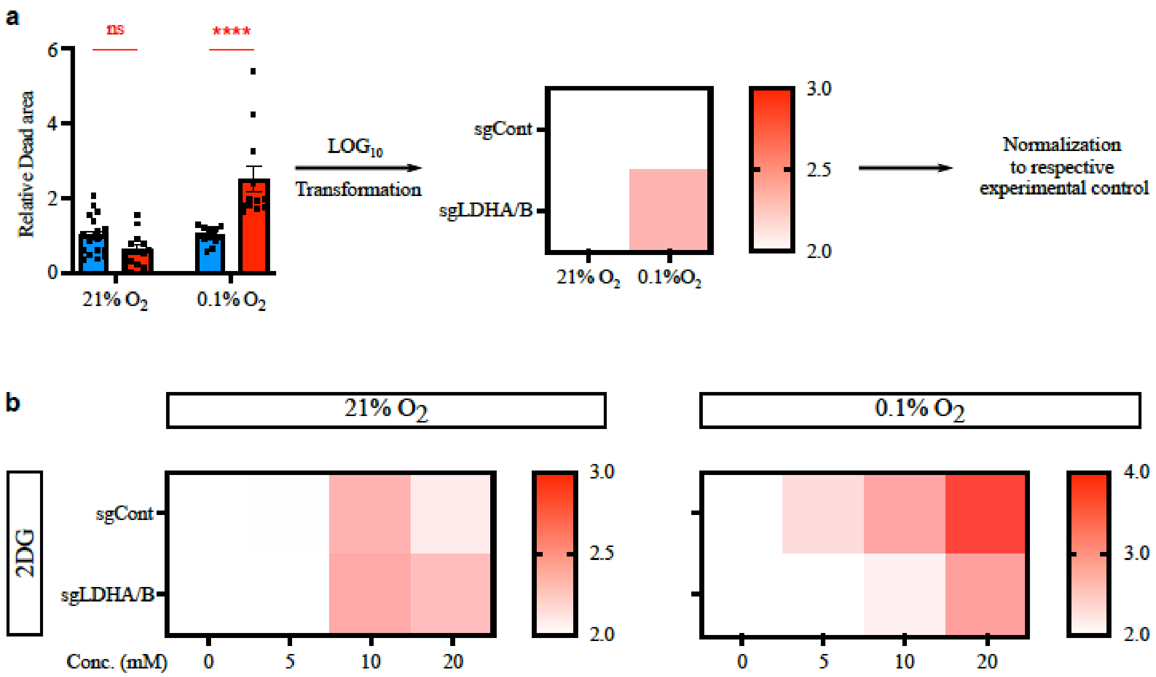

We did not perform in vivo experiments with 2-DG, as it was already published by others (https://doi.org/10.15252/embj.201798772 ; doi: 10.3390/cancers11020159), and also the efficacy on double LDHA/B KO was limited *in vitro*.

10. In Fig. 6d, LDHA/B KO upregulates many components of the TCA cycle as all the pyruvate is pumped into the TCA cycle. However how does LDHA/B KO upregulate enzymes of the glycolytic cycle?
Reply: This comment is related to reply of **point 5**. In **Fig 4D**, results showed that pyruvate is partially integrated in the mitochondria for fueling the TCA, as indicated by the increase of > 200 times in its intracellular abundance compared to control. This accumulation reflects the absence of the key enzymes of the lactic fermentation which is a reaction without limitations to maintain the redox potential (without LDHs, cells are accumulating NADH from glycolysis without possibility of regenerating NAD+). If reviewer 2 mentioned enzymes of TCA cycle (not glycolytic cycle), we can comment that these enzymes are allosterically regulated by the substrates and the products, they also depend on the cofactors, but are also regulated by other factors such as hypoxia (https://www.nature.com/articles/s41467-019-13668-3). For example,

the accumulation of pyruvate (and thus acetyl-CoA) blocks pyruvate dehydrogenase and directs the flow for producing OAA to maintain the flux of the cycle.

11. Fig. S6c is not readable.
Reply: This figure has been moved to new **Supplementary Fig S7**.

Please explain the values m+0 to m+10 in figure legend or text of Figure S7.
Reply: This has been added into the legend: m+0 stands for the fraction of metabolite without ^{13}Carbon and m+n (n > 0) stands for fraction of metabolite with n ^{13}Carbon. For example, m+5 correspond to a metabolite with 5 labeled ^{13}C. The sum of (m+0, m+1,…, m+10,…) equals to 1.

12. In Figure 6, the drug Stiripentol, which decreases LDH activity does not show a significant survival difference, however, combinational treatment with VEGF inhibitor Bevacizumab shows significant difference. Also, the difference between Bevacizumab only and combination treatment is only slightly significant, attributing all the survival difference to Bevacizumab and not Stiripentol. Thus, this data is not very convincing. Have the authors done a dose dependent study to test for Stiripentol IC50 values and toxicity in P3 cells? A combinational treatment with higher dose of Stiripentol along with irradiation could be attempted here.
Reply: We thank the reviewer for this comment and we agree on the moderate effect of stiripentol when coupled with bevacizumab, but this effect was found statistically significant when compared to control conditions. Moreover, we discussed this point with specialist of drug delivery in glioblastoma (Dr Junier, Sorbonne Univ, France), they said that, at 100 mg/kg, stiripentol induces a positive effect on one group which could be defined as "responders" (the animals responding to the drug, graph below, left part). However, we have increased the stiripentol to 150 mg/kg and this massively improved survival, as shown in the new graph in **Fig 6F** and below (right part). Of note, the dose of Stiripentol used in our first *in vivo* experiment was 300 mg/kg but it induced a strong inhibition of mouse mobility, impacting mouse wellbeing, which was considered as excessive molecule reaction by the local ethical committee.

Previous experiment (100 mg/kg Stiripentol)

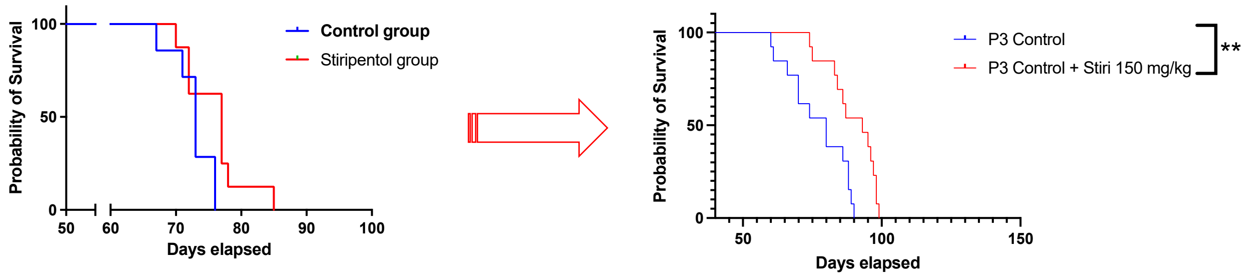

Minor
1. In Figure 1b,c, matrix embedded spheroid model, the authors should provide a quantification of the number of cells expressing either LDHA or LDHB in both the core and peripheral regions of the model.
Reply: We have quantified the intensity of LDHA and LDHB expression in the spheroid areas as defined in **Fig 1C**, as we believe that this is more accurate than counting number of cells only positive for LDHA and/or LDHB.

2. Please provide figure numbers to the main and supplementary figures.
Reply: This has been added in each figure.

3. In Fig. S4e, please plot the significance of double KO Annexin-V staining with respect to control.
Reply: This result was presented in the table of **Supplementary Fig 6E**. This is now better explained in the legend.

4. Please mention and explain Fig. S6a in the text.
Reply: Supplementary Fig 6A (now Supplementary Figure 8) was mentioned in both sentences "To explore the relationship between transcriptomic and metabolomic data, transcript levels of metabolism-related genes were incorporated into the central carbon metabolic network pathway map (**Fig 4D** and **supplementary Fig 8A-B**)." and in "To corroborate the RNA sequencing results (**Fig 4C-D** and **supplementary Fig 8A**), but we added extra explanations in the main text.

Reviewer #2 (Evidence, reproducibility and clarity (Required)):

In this study by Guyon et al, the authors demonstrate that targeting both LDHA and LDHB that deferentially express in glioblastoma (GB) cells and induced by hypoxia attenuates invasion and tumor progression of GB. The authors performed metabolomics and transcriptomics analyses using cells lacking LDHA/B and demonstrated that knockout of LDHA/B results changes in gene expression and metabolites in GB cells under hypoxic conditions. Genetic or pharmacological inhibition of LDH alone or in combination with irradiation further enhanced GB tumor growth inhibition and enhanced survival both in vitro and in vivo. Overall, this study has identified an interesting link between LDHA/B, hypoxia, and mitochondria metabolism, which is certainly interesting and relevant to the community who focus on cancer metabolism. However, several issues should be addressed through additional experiments and clarification to provide firmer mechanistic evidence in support of the major conclusions.
Reply: We thank the reviewer for these positive comments.

1. Quantification of distinct localization of LDHA and LDHB is needed for Figure 1 along with markers for hypoxic region and peripheral region of the tumor. In addition, colocalization of LDHA/B should be quantified and graphically presented.
Reply: As discussed in **Reviewer 1 (point 7)**, double staining of LDHA and LDHB required specific procedure of tissue fixation which led to complications for staining with other markers. We have already performed some image quantification of LDHA and LDHB staining, as shown in **Fig 1A**. We also performed double staining of CAIX (carbonic anhydrase IX) and LDHA which shows colocalization of both proteins in the core hypoxic area. LDHA staining is absent in the border area and some invasive cells are LDHA-positive, as previously described (images below). This is now shown in **Supplementary Fig 1**.

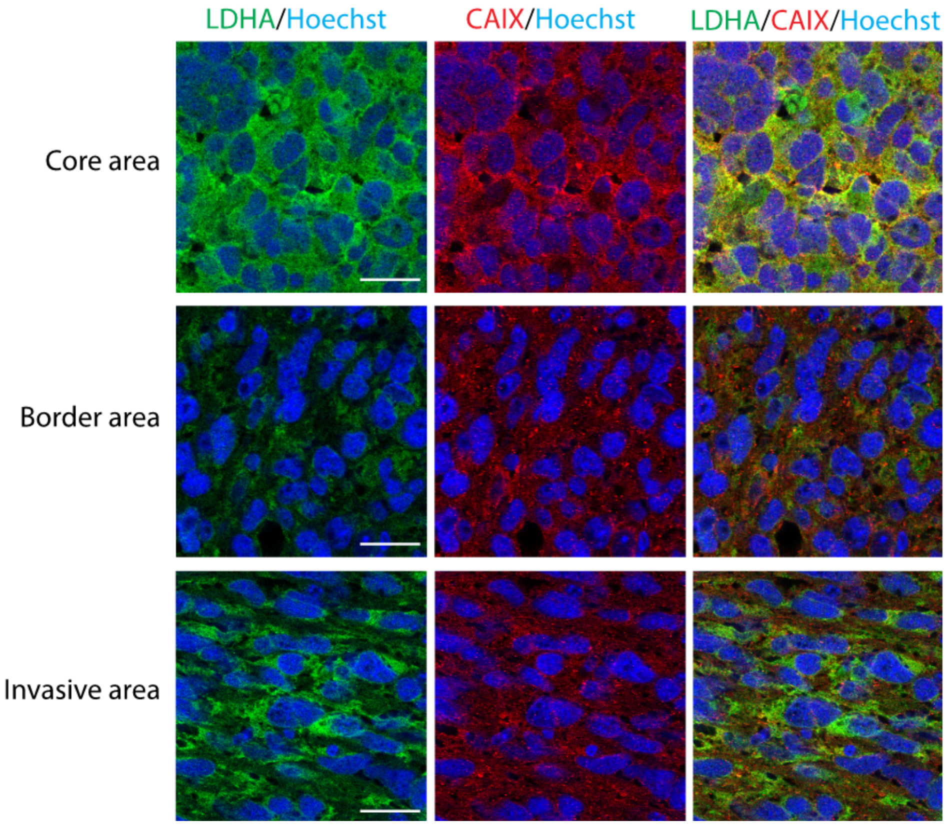

Moreover, antibodies used in this study to detect LDHA and LDHB are specific for the human epitopes (**Fig 1A**). Peripheral region can be detected by using these antibodies but also tumor cells harbor bigger nuclei (green nuclei in the figure below, right panel) than cells from microenvironment (black nuclei in the figure below).

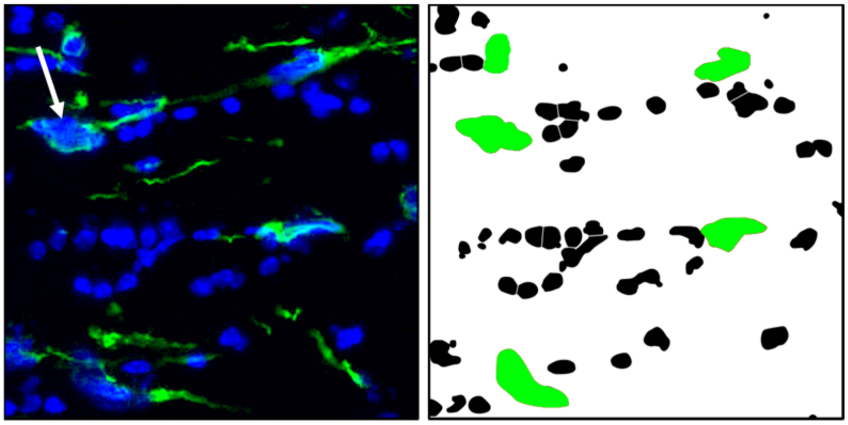

Colocalisation of LDHA/B has been quantified in supplementary Figure 1 and only few cells are expressing both isoforms.

2. What is specifically inducing LDHA not LDHB under hypoxia (Figure 2a)?
Reply: It has been previously characterized that *LDHA* promoter contains Hypoxia-Responsive Element (HRE) which is not present in *LDHB* promoter. *LDHB* has been characterized to be regulated by PGC-1a (https://www.jbc.org/article/S0021-9258(20)34525-7/fulltext).

Why same concentration (20 mM) of external lactate attenuate proliferation but promote GB cell invasion (Figure 2e-g)?
We hypothesized that lactate favors invasion over proliferation as suggested in the "go-or-grow" hypothesis (recently reinforced by our collaborators/co-authors from MILO lab in their Cancer cell paper - doi: 10.1016/j.ccell.2022.05.009) and in our observations, even if we can observe a smaller but efficient proliferation of lactate-treated cells when compare to control cells. We generated a longer proliferation recording to confirm results presented in **Fig 2E**:

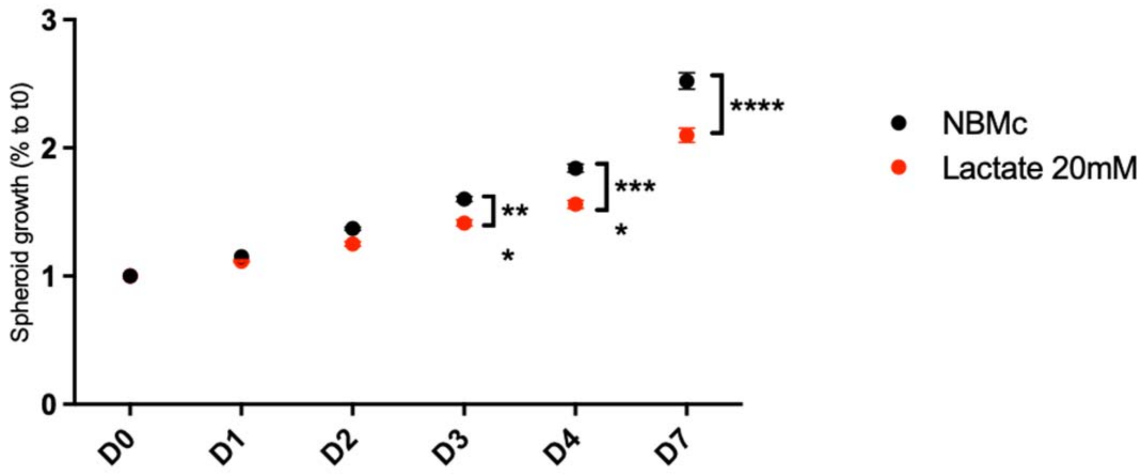

Does this difference brings back to the switch between glycolysis and OXPHOS. The authors at least should discuss potential underlying mechanisms.
We observed this phenomenon quickly after lactate infusion (<24h), lactate is directly fueling Krebs cycle for efficient energy production, then incorporation of 13C in metabolites present in neoglucogenesis was observed for sustaining biomass synthesis (unpublished results, from **Supplementary Fig 4**), but is not sufficient in longer term to sustain proliferation as observed in glucose-enriched medium (NBMc above). We can hypothesize that in our cell model oxidative phosphorylation promotes invasion whereas glycolysis regulates proliferation. Some modifications were inserted in the discussion to better discuss these points.

3. The authors distinguished LDH activity and LDHB activity in LDHA/B knockout cell lines. However, it is unclear how the activities of LDHs and LDHB were distinguished. The legend states that they were colorimetric analyses but should be described in more detail. If LDHB activity was by using enriched LDHB through immunoprecipitation, the same approach should be made for LDHA.
Reply: We agree with the reviewer and we can comment that no commercial kit has been developed for specifically measure LDHA activity. We, therefore, performed an equivalent

experiment for measuring LDHB activity, as previously published by our colleagues (Ji *et al*, 2017, Nature Comm, DOI: 10.1038/ncomms15308).

Here is the protocol:

LDHA immunoprecipitation and enzymatic activity assay

LDHA was immunoprecipitated and the enzymatic activity measured as previously described (DOI: 10.1038/ncomms153085308; DOI 10.1074/jbc.RA118.004180).

Immunoprecipitation: In brief, cells were incubated for 4 h in a buffer containing 0.3% Nonidet P-40, 150 mM NaCl, 50 mM Tris-HCl (pH 7.5), inhibitors of proteases, and phosphatases. Lysates, obtained by centrifugation at 13.000g for 10 min, were pre-cleared with protein A/G agarose beads (16% of sample volume) for 1h. After a short centrifugation, pellets were discarded and the protein content of the supernatants measured by Bradford assay. For each experiment, samples were diluted to obtain equal protein concentration. Extracts were incubated overnight with anti-LDHA antibodies (LDHA sc-137243, Santa Cruz Biotechnology) at the final concentration of 9 ng of Ab/µg of proteins. Afterward protein A/G agarose beads (16% of sample volume) were added to the lysates, which were incubated for further 4h. After a short centrifugation, the pellets containing LDHA proteins coupled to the agarose beads were washed once and re-suspended in Tris-HCl 0.2 M pH 7.3. All the centrifugations steps were performed at 4°C, incubation were carried out at 4°C with gentle shaking. Where indicated, cells were incubated at 0.1% of oxygen for 48h.

Enzymatic assay: LDHA immunoprecipitated proteins were added to the reaction buffer (0.2 M Tris-HCl pH 7.3, 0.05% BSA, 10 mM $MgCl_2$, 2 mM pyruvate, 0.5 mM NADH) and the enzymatic activity was determined by measuring NADH oxidation (reduction in absorbance) at 340nm at 25°C using a CLARIOstar (BMG Labtech).

Results obtained with this method were perfectly aligned with the results presented in the first manuscript. These results are now in **Fig2C and Supplmentary Figure 5B.**

On a related note, the authors observed minimum effect on spheroid growth when LDHB was deprived. It is worth to know the expression levels of LDHA and LDHB in GB cells or patient tumors.

Reply: As shown in **Fig 3A**, expression of LDHA and LDHB does not differ when the other subunit is depleted:

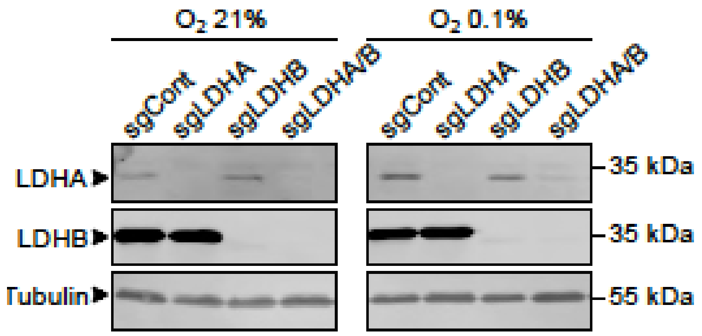

LDHB is highly expressed when compared to LDHA expression in P3 cells. When checking LDHA and LDHB expression in ProteinAtlas database, we can observe that

LDHB is also more expressed in glioma patients than LDHA (value of 183.4 FPKM for LDHB and of 99.2 FPKM for LDHA).

4. It is unclear why only ETC1 inhibitor phenformin was used and compared to irradiation for tumor growth inhibition in LDHA/B KO cells in Figure 5d. Based on the immunoblot of Figure 5a, factors involved in ETC 2 (SDHA), 3 (UQCRC2), and 4 (COX II) were also increased. The authors need to check whether inhibitors against ETC 2/3/4 alter viability of GB cells that lack LDHA and LDHB.

Reply: We thank the reviewer for this interesting comment. Phenformin was used *in vivo* for reproducing results from **Fig 2G** when rotenone was used in spheroid invasion assay. Our aim was to specifically inhibit both regulators of cellular redox in the tumor cells (lactate dehydrogenases and ETC1).

Then, we measured cell viability, by using dead/live kit, in control and LDHA/B KO cells when adding inhibitors of ETC2 or ETC3 such as Aptenin A5 or antimycin A, respectively. Inhibiting ETC3 and ETC4 will lead to toxic effects as antimycin A induces letal accumulation of ROS and cyanide inhibits all peroxidases. To conclude, antimycin A and cyanide will have pleiotropic effects which will hide a precise answer. Though, we tested antimycin A treatment on spheroids and most of the cells were dying, thus, results were not presented in the revised manuscript. However, we then generated new results based on Aptenin A5 treatment on cell viability and we observed a small increase of cell death in double LDHA/B KO cells in both oxygen conditions, and more specifically in hypoxia, when compared to the control group. Results were inserted below and in **Supplementary Figure 12B**.

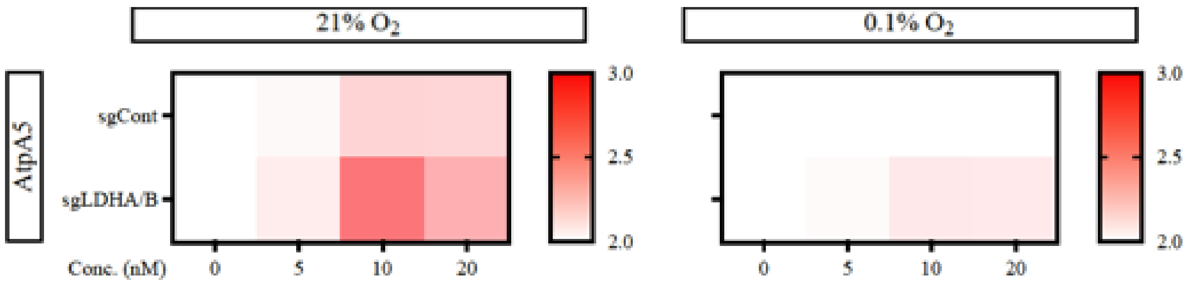

5. On a related note, it is unclear why the authors used LDH inhibitor and VEGF inhibitor in combination in Figure 6. Based on Figure 5, it seems targeting LDH in combination with radiation therapy is a good direction. It is necessary to provide why radiation and VEGF inhibition approaches were used in combination with LDH inhibition. In addition, both radiation and VEGF inhibitor should be treated in combination with the LDH inhibitor stiripentol and compare which would be more beneficial treatment for GB. If there are any additional drugs that are routinely used in GB, these need to be included in the combination study. Especially because the combination of VEGF inhibitor and LDH inhibitor seems not beneficial at all in vivo.

Reply: VEGF inhibitor (bevacizumab) was used in **Fig 6** to improve stiripentol accessibility, as observed in our previous study (Daubon et al., Nature Communications 2019) and when used on double LDHA/B KO in **Supplementary Figure 6J**, it improves survival. Bevacizumab was characterized to improve vessel quality and molecules penetration in the tumor, it appears that

stiripentol follows the same trend that the one observed with anti-thrombospondin-1, as published earlier by our team.

As replied to **Reviewer 1 (point 12)**, a new experiment has been performed by treating implanted animals with a higher dose of stiripentol (150 mg/kg) and the results show that it has a strong effect on tumor growth. **Figure 6** has been modified to integrate the new results of mice treated with stiripentol 150 mg/kg which clearly shows the benefit of stiripentol. Other extra experiments are performed in the lab to evaluate the effects of stiripentol on recurrent tumors but we consider now that the new results are strong enough to support our contention.

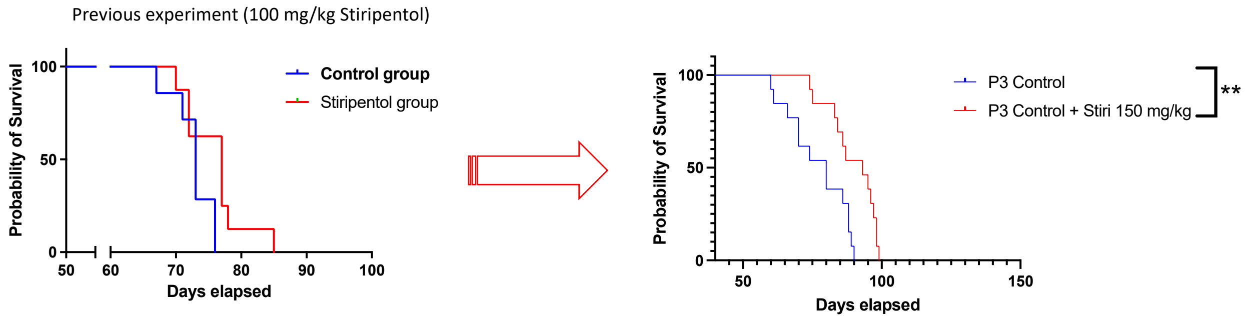

6. The authors state in their abstract that lactate is the metabolite that replenishes the TCA cycle, and this is based on the metabolomics data obtained using LDHA/B KO. How are the authors sure that all these changes including alteration of metabolites and metabolic gene expression (Figure 4c) are due to lactate but no other metabolites in cells lacking LDHA/B? Unless the authors demonstrate that replenishment of lactate restore these changes, it is hard to conclude that the driver was lactate.

Reply: We thank the reviewer for this important comment. Without LDHs, cells are not able to retroconvert lactate into pyruvate and then, replenish the TCA cycle. As an example, we have previously generated results based on invasion experiments. We have treated double LDHA/B KO spheroid with lactate or HCl (to mimic acidic pH) and measure invasion rate. The same increase of double LDHA/B KO cell invasion was observed when treating with lactate of HCl, which is much lower than the one observed on control spheroids, as depicted below:

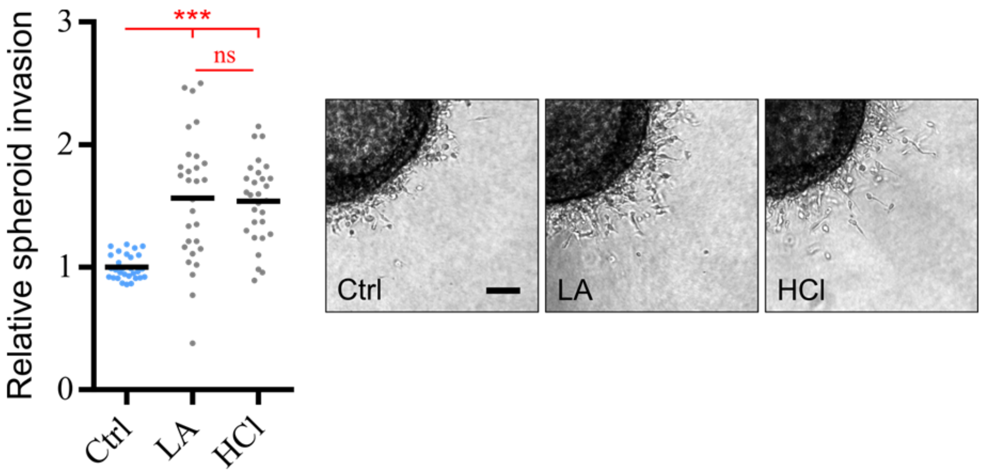

We can conclude that lactate cannot be used as a metabolite to sustain cell invasion of double LDHA/B KO. Acid pH induced by LA and HCl triggers small increase in invasion rate (rate of 1.5 compared to a value of 3.8 when wild type spheroids are stimulated with lactate, **Fig 2G**). To finish, our study mainly focused on impact of LDHA/B KO, leading to redox imbalance, and metabolic stress. Lactate absence is a consequence of this specific KO.

Reviewer #2 (Significance (Required)):

Please see above comments.

Reviewer #3 (Evidence, reproducibility and clarity (Required):

Summary

The publication of Guyon et al reports that LDHA and LDHB contribute to the growth and invasion of glioblastoma stem-like cells by supporting the TCA cycle to maintain energy metabolism in hypoxic tumor areas. By creating single or double-knockout LDHA/B GB cell lines as well as by metabolomics and transcriptional analyses, the authors identify changes of genes and metabolites involved in energy metabolism and report that lactate fuels oxidative phosphorylation in order to sustain tumor development. While LDHA and LDHB alone were shown to be insufficient therapeutic targets, a combined knockout of LDHA and LDHB significantly inhibited the malignant phenotype of patient-derived GB stem-like cells. In addition, in vivo mouse studies with intracranial tumor injection with LDH activity inhibited by the antiepileptic drug stiripentol in combination with the antiangiogenic drug bevacizumab were performed. Guyon et al conclude that the activity of LDHA and LDHB in GB is a potential new target for glioblastoma therapy when combined with other drugs or irradiation.

Major comments

Point 1 : The introduction of the publication does not cover all relevant information. First, the authors only describe the two main LDH subtypes LDH-A and LDH-B and leave out the other two LDH subtypes LDH-C and LDH-Bx. Furthermore, in the human body LDH exists in five different isozymes composed of four subunits, i.e. LDH-1 to LDH-5 (Drent et al, 1996; DOI: 10.1183/09031936.96.09081736). Depending on the composition of the LDH isozyme available in the brain, more information can be drawn from the presented data. The publication of Bittar et al from 1995 for instance describes the dominant LDH isozymes in astrocytes and neurons (DOI: 10.1097/00004647-199611000-00001). Further information is necessary for an improved interpretation of the published data. In general, some information in the introduction is not cited at all.

Reply: We thank the reviewer for this comment and we completed our introduction based on these recommendations. Of note, LDH-C and LDH-D are detected at very low level, as reported in brain tissues (protein atlas database). Our results based on *in vivo* RNA sequencing previously published (Daubon et al, Nature Communications 2019) showed no expression of LDH-C and very low of LDH-D. LDH-Bx cannot be detected as there is only a slight difference with LDH-B of 7 supplement amino acids. Moreover, LDHC was characterized as mainly germinal, and LDH-D mainly metabolizing D-lactate. This was added in the revised manuscript.

Other passages could be removed since they are not important for the story and hence are confusing (i.e. the information about NMDAR is not required).
This sentence has been removed.

A reagent list cannot be found in the supplemental documents.
The reagent list can be found as supplemental file named: Reagent List.

Point 2: Figure 1 shows the comparison of a healthy mouse brain with human GB stem-like cells. This comparison is not suitable because different species can express a different pattern of LDH isozymes. Furthermore, since the brain is one of the most complex organs, it is unclear which area of the brain was used as control and if there is a general difference in the LDH expression pattern in different brain areas. An age-dependent change of LDH expression should also be excluded by appropriate experiments.
Reply: As previously explained to **Reviewers 1 and 2**, staining procedure of LDHA and LDHB required extensive work in the P3 tumor model. Control areas were mainly taken in striatum of mice from 16- to 18-week-old. As shown in **Reviewer 1 (point 1)**, LDHA and LDHB antibodies used specifically recognize human proteins, as shown in the graphs from **Fig 1A**.
However, we consider that checking expression of LDHA and LDHB in the physiological brain is outside the scope of our manuscript, as we mainly focused on tumor areas.

Were the results confirmed with another GB cell line, i.e. big cells?
To confirm our results obtained with P3 cells, we analyzed the expression of LDHA and LDHB in patient samples, with our collaborators from University of Freiburg who just published a milestone article on spatial resolution of GB samples ("Spatially resolved multi-omics deciphers bidirectional tumor-host interdependence in glioblastoma" Ravi et al., Cancer Cell, Jun 2022). These results are presented in the revised manuscript, in **Fig 1D-E** (and below) and are perfectly in correlation what was primarily found in our P3 tumor model, LDHA being expressed in hypoxic areas and in some invasive cells, and LDHB mainly in invasive areas (infiltrative cortex).

Review COMMONS

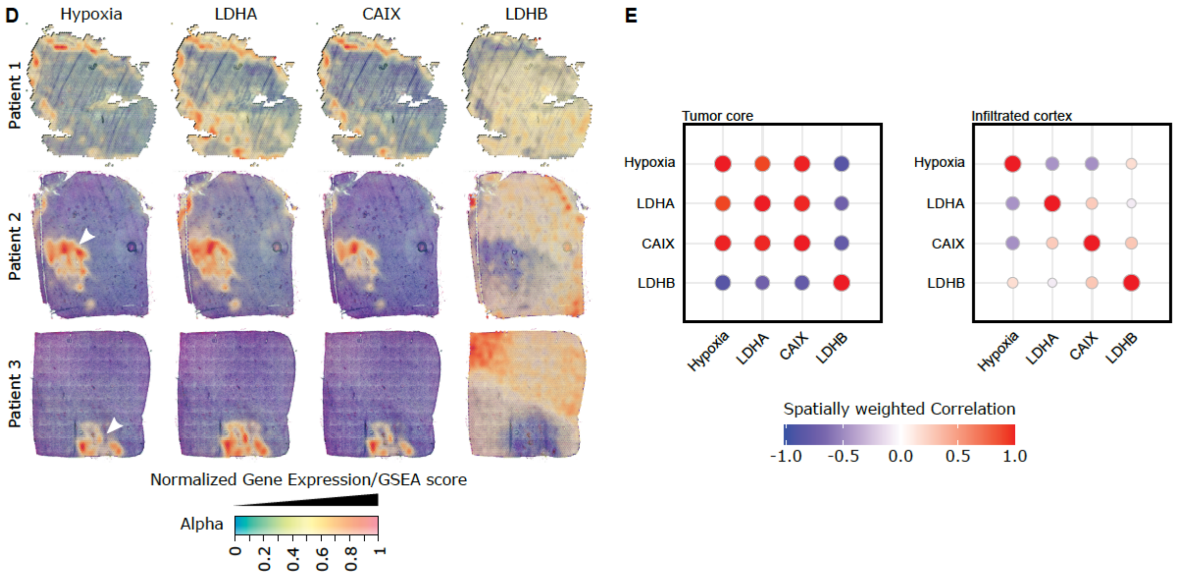

The age of the mice and the number of tested animals should be specified. Statistical analysis of biological replicates should be included.
This has been answered in this **Point 2** reply and specified in the text.

At present the text does not explain why the spheroid model was used in addition to the in vivo model. One may argue that in the in vivo model it is unclear whether the cells at the invading edge are tumor cells or healthy therefore, the spheroid model is important and shows that the peripheral tumor cells show a different LDH composition that the ones in the tumor core. This should be explained in the text.
We characterized in several previous studies the importance of using such spheroid model as a simplified avatar of tumor, as mentioned in the text "a model that recapitulates GB oxygenation regional heterogeneity (ref[1])".
To distinguish between tumor or healthy cells, we have performed an experiment in which GB cells were expressing GFP and analyze the size of the nuclei which are bigger than nuclei from mouse microenvironment. Moreover, cells in the corpus callosum modify their shape for getting mesenchymal phenotype:

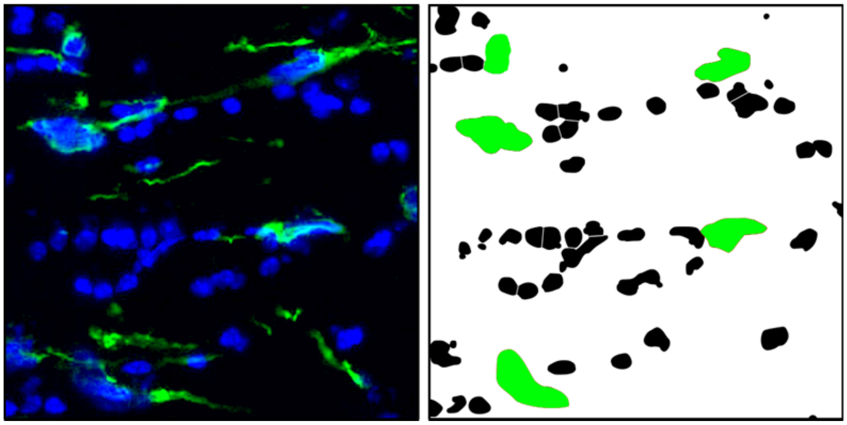

In general the text can be improved.
We thank the reviewer for this comment and we improved the description of **Fig 1** (sentences in red), but also the whole text.

Point 3: In figure 2 only the activity of total LDH and of LDHB is shown, but information about the activity of LDHA is missing. Since other LDH subunits than only LDHB and LDHA could be present, the activity of LDHA should be included.
Reply: As replied to **Reviewer 2 (point 3)**, we performed an equivalent experiment for measuring LDHB activity, as previously published by our colleagues (Ji *et al*, 2017, Nature Comm, DOI: 10.1038/ncomms15308). Here is the protocol:

LDHA immunoprecipitation and enzymatic activity assay
LDHA was immunoprecipitated and the enzymatic activity measured as previously described (DOI: 10.1038/ncomms153085308; DOI 10.1074/jbc.RA118.004180).
Immunoprecipitation: In brief, cells were incubated for 4 h in a buffer containing 0.3% Nonidet P-40, 150 mM NaCl, 50 mM Tris-HCl (pH 7.5), inhibitors of proteases, and phosphatases. Lysates, obtained by centrifugation at 13.000g for 10 min, were pre-cleared with protein A/G agarose beads (16% of sample volume) for 1h. After a short centrifugation, pellets were discarded and the protein content of the supernatants measured by Bradford assay. For each experiment, samples were diluted to obtain equal protein concentration. Extracts were incubated overnight with anti-LDHA antibodies (LDHA sc-137243, Santa Cruz Biotechnology) at the final concentration of 9 ng of Ab/µg of proteins. Afterward protein A/G agarose beads (16% of sample volume) were added to the lysates, which were incubated for further 4h. After a short centrifugation, the pellets containing LDHA proteins coupled to the agarose beads were washed once and re-suspended in Tris-HCl 0.2 M pH 7.3. All the centrifugations steps were performed at 4°C, incubation was carried out at 4°C with gentle shaking. Where indicated, cells were incubated at 0.1% of oxygen for 48h.
Enzymatic assay: LDHA immunoprecipitated proteins were added to the reaction buffer (0.2 M Tris-HCl pH 7.3, 0.05% BSA, 10 mM $MgCl_2$ 2 mM pyruvate, 0.5 mM NADH) and the enzymatic activity was determined by measuring NADH oxidation (reduction in absorbance) at 340nm at 25°C using a CLARIOstar (BMG Labtech).

Results obtained with this method were perfectly aligned with the results presented in the first manuscript. These results are now in **Fig 2C and Supplementary Figure 5B.**

Point 4: In figure 3 it is unclear if the hypoxic condition was at 0.1 % O2 (3a and 3f) or at 1 % O2 (3b). In addition, the oxygen concentrations in the figure differs from the indicated values in the figure description. The hypoxic conditions vary in the publication from 0.1 % O2 to 1% O2 (figure 4) and can therefore not be compared. Any explanation of the different conditions is missing.
Reply: We apologize about these discrepancies. We used 0.1% hypoxia for all experiments in **Fig 2**, as we performed assays on control spheroids. Then, in **Fig 3 and 4**, 0.1% O_2 hypoxia was used for short experiment such as invasion assay or metabolomics experiments (maximum 2 days) and 1% O_2 for longer experiments such as proliferation assay (around 4 to 7 days). This was due to low viability of double LDHA/B KO cells observed at 0.1% 0_2, as already demonstrated for 1% O_2 in **Fig 3D**.

Review COMMONS

We thank the reviewer for pointing out that a mistake was inserted in the legend of **Fig 4**, as the experiments were done at 0.1% O_2. This is now corrected.

Presented western blot data should be quantified according to figure 2a.
Western-blots were quantified as shown here (related to tubulin):

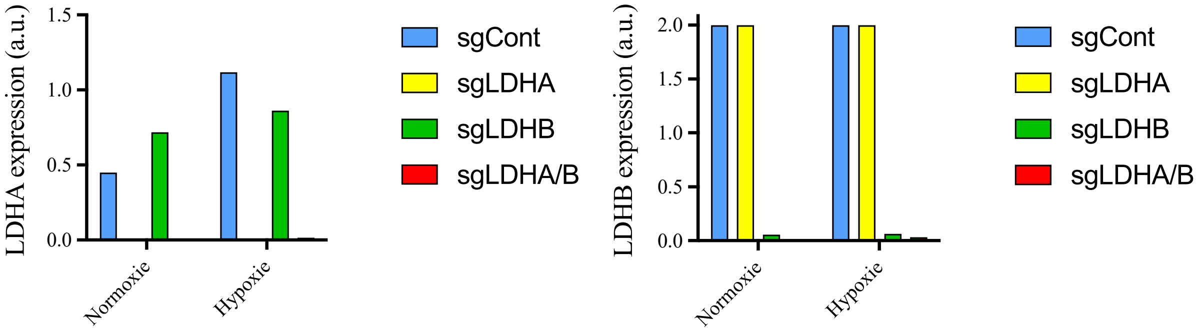

We decided to not include this quantification as it only confirms that our KO of LDHA and LDHB worked.

Point 5: Figure 3f is not required for a standard in vivo experiment, the method description is sufficient.
Reply: We removed this panel and respective legend as suggested by the **Reviewer 3**.

Point 6: It is striking that mice bearing LDHB knockout tumors show significantly lower survival than those with a knockout of LDHA and LDHA/LDHB (Fig 3i) but the tumor core and tumor invasion (Fig 3b) did not show differences. What is the reason for this observation? In the discussion the authors describe hemorrhages at the tumor site of mice bearing LDHB KO tumors. This should be supported by appropriate data and also dealt with in the results and more extensively in the discussion. Were hemorrhages also present in the double knockouts and if not, why not?
Reply: As shown in the figure below, we can detect that blood vessels from KO LDHB are larger than in the other tumors, such as control tumors. We are actually precisely quantifying blood vessel density and will bring more details in the final revised version of our manuscript. Of note, no hemorrhage was observed in double LDHA/B KO as no lactate was produced by the tumor cells, leading to low tumor acidosis. It has been characterized by Pierre Sonveaux group that lactate and acidosis lead to neo-angiogenesis (doi:10.1158/0008-5472.CAN-10-2828; https://doi.org/10.1371/journal.pone.0033418).
Below an example of blood vessel density (anti-CD31 staining, in red) in control and LDHB KO tumor:

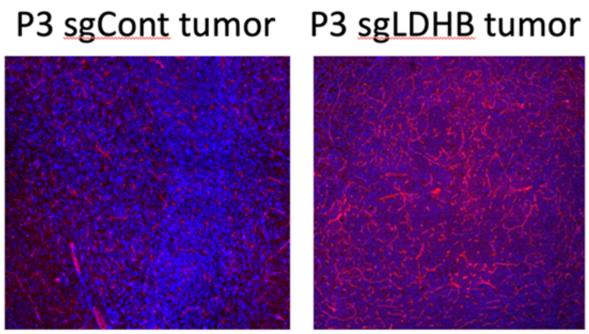

We quantified the vascular density in mouse brain slices (anti-CD31 staining) that shows increased vascular coverage of LDHB KO cells compared to control, LDHA KO and double LDHA/B KO (**Supplementary Fig 5H**).

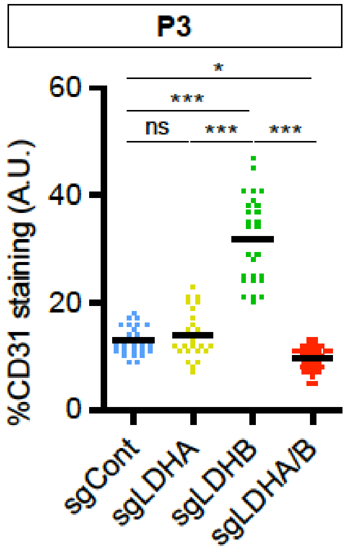

This point is also discussed in **Reviewer 1 Point 6.** The discussion was also improved to better explain this point.

Point 7: The data shown in 3g and h raises various questions:
How is the tumor core defined? How do you differentiate between tumor core cells and invasive GB cells? The indicated areas for the cell invasion, i.e. the red square in the histological images, do not cover the complete dark region (assumed as the invasive area), why did you exclude the rest? The quantification is unusual and unclear and therefore difficult to assess.
Reply: The implantation is proceeded into the striatum (red star) and tumor is growing by invading the corpus callosum as shown in the images below (white stars):

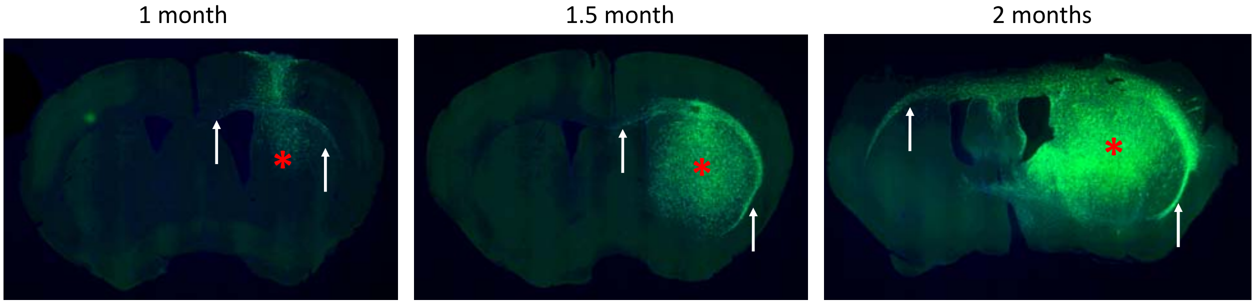

Then, tumor core is mainly defined as the area in the striatum and invasion is quantified in the corpus callosum, which are visible and easily quantified brain areas, as shown in the figure below:

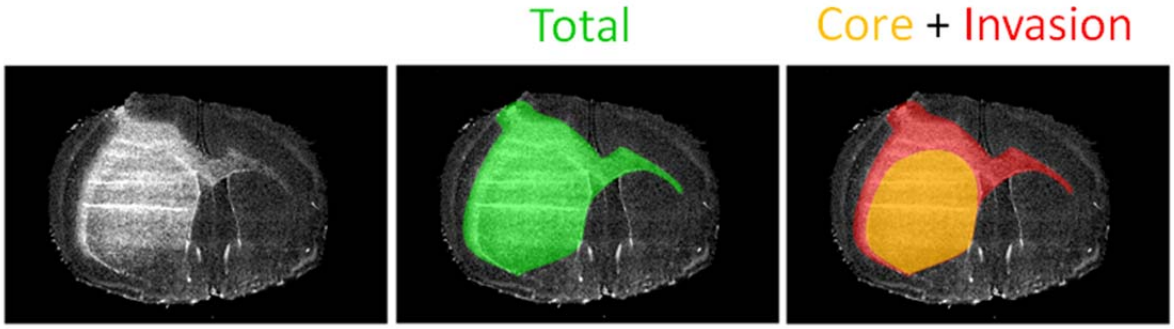

How old were the mice in g and h, were they all sacrificed at the same day after tumor injection?

We had to perform two different *in vivo* experiments as control cells in **Fig 3G** only contain one CRISPR-cas9 plasmid and control cells in 3h contain both puromycin- and blasticidin resistant constructs. This control was first employed to verify if growing cells in both antibiotics would impact general proliferation, which was not the case, and this is why we could generate a second set of experiments (**Fig 3H** in the new Figure). The mice were of 16-week-old and 20-week-old in **Fig 3G** and **3H**, respectively.

The unit of the tumor core and the tumor invasion, meaning in relative to brain, is unusual and needs further explanation.

We have changed the graphs and applied new calculations for putting a unit to quantify core and invasive areas in **Fig 3** and **6** (in mm^2).

Point 8: While 3g compares the single knockouts with the control, 3h only compare the double knockout with the control. How do single and double knockouts compare?

Reply: This has been commented in **point 7**, second reply.

Point 9: Figure 4 shows the metabolic and transcriptomic status of the created single and double-

knockout LDHA/B cell lines and could be improved by including the metabolic and transcriptomic data of these cell lines under normoxic conditions, i.e. in a supplemental figure.
Reply: This new analysis has been done and are now presented in **Supplementary Fig 9** and **10**.

Point 10: Phenformin inhibits the oxidative phosphorylation in cells thereby altering their energy metabolism. Since this effect impacts not only tumor cells but the whole body, please add additional information on the impact of phenformin in healthy individuals. The used concentration of phenformin in figure 5 is not explained in the paper. Was the applied concentration determined in an in vitro pretest? Was it found in other publications? How can the authors conclude that the employed concentration was sufficient to inhibit oxidative phosphorylation? Further information are required.
Reply: Phenformin was used as complex I inhibitor, to link results obtained with rotenone in *in vitro* invasion assays or respirometry. Of note, phenformin was found efficient in control tumors but not in double LDHA/B KO (**Fig 5E**), suggesting that the concentration used in this experiment was inducing a good bioavailability of the drug, as suggested in several publications (Jiang et al, 2016 Oncotarget, doi: 10.18632/oncotarget.10919). This is now better explained in the discussion. The dose used was then based on previous published protocols.

Point 11: In figure 6 the combined treatment of bevacizumab and stiripentol is shown. How efficient is the inhibition of the LDH activity in the cell lines by stiripentol? This could be easily investigated by a dose-dependent in vitro experiment.
Reply: We have performed an experiment by using 100 and 500 µM stiripentol concentration and invasion was recording in our collagen assay. As shown below, there is a dose-response which is observed by using either 100 or 500 µM stiripentol concentrations on invasion capacities but not on proliferation:

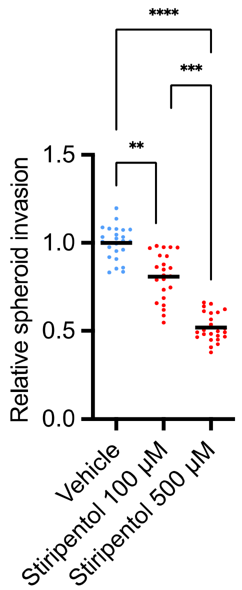

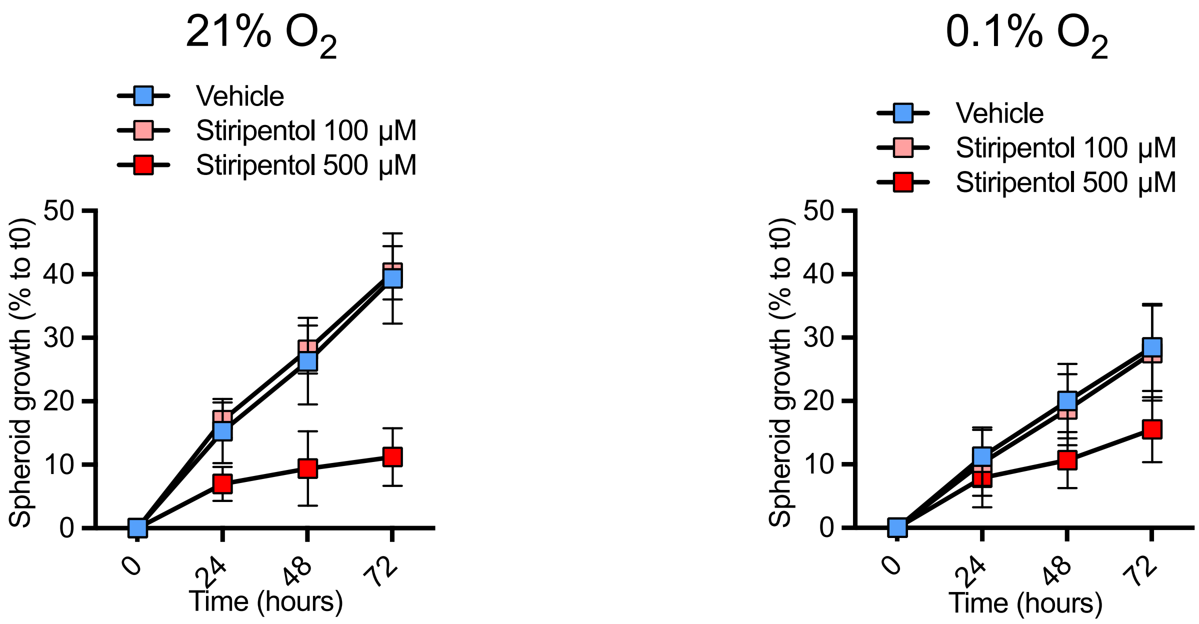

The concentration of 500 µM was used based on those first results.

Point 12: In order to determine the effect of the combined treatment, the combo needs to be statistically compared to both single treatments, not only to the vehicle. Figure 6f and the tumor core data of 6g show that mainly bevacizumab is important for a better survival of the animals. This has to be stated in the manuscript and also modified in the abstract.

Reply: As this question was raised by all reviewers, we generated a new *in vivo* experiment in which stiripentol was injected at 150 mg/kg in glioblastoma-bearing animals. Survival of stiripentol-treated animals was strongly increased (**Fig 6F** and below). This response is linking to the **reply to point 12 of reviewer 1** and the **reply to point 5 of reviewer 2**.

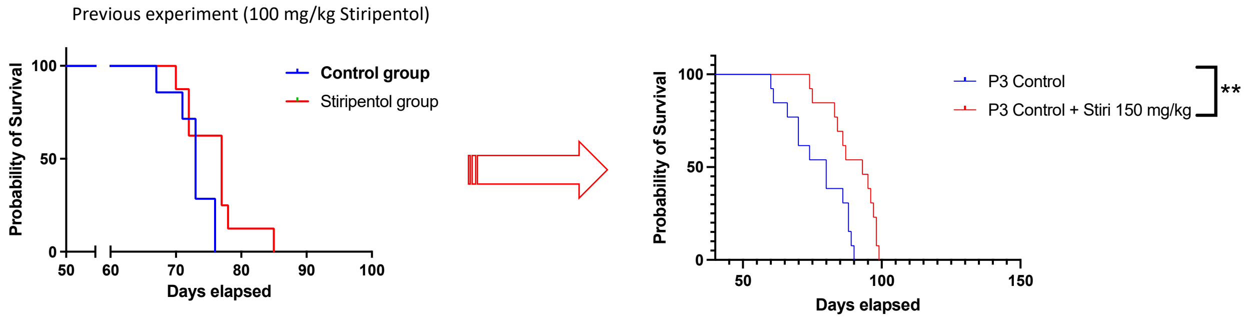

Point 13: As mentioned above, further information about the calculation of the tumor core and the tumor invasion is missing as well.

Reply: This is now explained in point 7 and modified in **Fig 3** and **6**.

Does the combination of LDHA/B knockout with bevacizumab show a combined effect?

Reply: The result was presented in **Supplementary Fig 6J** (*** statistical differences, p<0.001).

Point 14: In general, in figure 1 it was shown that spheroids have a different LDH expression pattern at different regions. The comparison of injected spheroids displaying regional-dependent

LDH expression patterns with the metabolic and transcriptional analysis of cultivated GB cells in suspension could greatly differ in their metabolism and thereby not reflect the situation in the tissue.

Reply: We agree with the reviewer that *in vitro* and *in vivo* results could be different, obviously, but we claim in the article than spheroid can efficiently recapitulate *in vivo* situation, as shown in our results. As an example, we found same results *in vitro* and *in vivo* results for double LDHA/B KO which inhibits spheroid and tumor growth and invasion. We do believe that this experimental procedure is a strength for neuro-oncology studies.

Minor comments

The text needs to be checked for grammatical errors. The experiments should be described in a way that they can be reproduced. References should be checked and included where they are missing, i.e. in the introduction. Hypoxic conditions should be made clear to avoid any confusion. In general, check if all necessary information for each figure is included in the figure and the figure description, do not exclusively insert important information in the main text.

Reply: We carefully corrected the manuscript, we added more references in the introduction and discussion parts.

Reviewer #3 (Significance (Required)):

The manuscript is of moderate novelty. The presentation of lactate accumulation and the increased activity of LDH under hypoxia is presented for the specific cancer type GB. Nevertheless, most of the information is already known for most cancer types (i.e. Warburg effect). The main significance of this paper relies in the potential co-therapeutic targeting of LDHA and LDHB in GB patients.

Reply: The reviewer considers our article as of moderate novelty but we can underline that we generated original results which, we believe, will be of importance for a broad audience. Our article does not rely only on Warburg effect, which is the adaptation of cells after hypoxia, but also rely on lactate symbiosis between hypoxic cells and oxygenated areas, as suggested in some previous work. Metabolic symbiosis is of great importance, as discussed into details in the review of Corbet and Feron ("Tumour acidosis: from the passenger to the driver's seat": doi:10.1038/nrc.2017.77). We added recent and important data from our collaborators and new co-authors (Kevin Joseph, Vidhya M. Ravi and Dieter Henrik Heiland ; "Spatially resolved multi-omics deciphers bidirectional tumor-host interdependence in glioblastoma" Ravi et al., Cancer Cell, Jun 2022) in which we can observe a regional expression of LDHA and LDHB in patient samples has been observed compatible with a lactate-dependent metabolic symbiosis in patient samples. Here, we show that GB cells cultured in hypoxia can fuel cells from oxygenated area by feeding them with lactate, to trigger cell invasion, in a symbiotic way. This is the first time that the role of both LDHA and LDHB is described in such a great detail in GB including RNA sequencing coupled with ^{13}C metabolomics. We also discuss extensively how LDHA/B KO cells adapt to severe hypoxia. This is the first time that regulation of respiratory chain

complex expression is shown in that context. Moreover, we repurposed the antiepileptic drug stiripentol and validate the drug in a preclinical orthotopic GB model. A massive increase in survival was observed when compared to the control group.
This has been better explained in the revised manuscript.

My expertise
Long-standing experience working on amino acid metabolism in glioblastoma.

3. Description of the revisions that have already been incorporated in the transferred manuscript

All the experiments asked by the reviewers were performed

4. Description of analyses that authors prefer not to carry out

All the experiments asked by the reviewers were performed.

5. **Comment from Editor and Referee #1 on 2nd of November 2021:**

Thank you for the submission of your research manuscript to our editorial offices. I have now had the opportunity to read your manuscript, as well as the referees' reports and your rebuttal letter, and to discuss them with the other members of our editorial team. We appreciate that you are willing to address/have addressed the points raised by the referees, however I am afraid we do not think the manuscript is well suited for publication in EMBO Molecular Medicine and have therefore decided not to proceed with peer review.

Indeed, we appreciate that your study investigates LDHA/B contribution to glioblastoma progression and invasion, and that you propose a new therapeutic strategy based on the use of the anti-epileptic drug stiripentol combined with the anti-angiogenic bevacizumab. However, we are not convinced that the translational advance is sufficient for publication in EMBO Molecular Medicine, as the strong anti-tumor effects observed might be due to bevacizumab. We realize that you are proposing to address this by performing further experiments, but the issue of these experiments remain uncertain at this stage. Furthermore, the new therapeutic strategy is not placed in the context of current standard of care.
Reply: We have simplified the *in vivo* experiment and found that stiripentol in monotherapy at a concentration of 150 mg/kg is efficient for improving mouse survival. The new results are now presented in Figure 6F. Further studies will be performed by combining TMZ or irradiation and stiripentol, but we believe that these new results are strong enough to support our results.

7th Sep 2022

Dear Thomas,

Thank you for the submission of your manuscript to EMBO Molecular Medicine, and please accept my apologies for the delay in getting back to you as I was out of office when the referees' reports came back. We have now received the enclosed reports from the three reviewers who had initially reviewed your manuscript. As you will see, they are now supportive of publication, and I am therefore pleased to inform you that we will be able to accept your manuscript once the following points will be addressed:

1/ Main manuscript text:
- Please answer/correct the changes suggested by our data editors in the main manuscript file (in track changes mode). This file will be sent to you in the next couple of days. Please use this file for any further modification.
- Thank you for providing the 'Data Availability' section. Please note that the Data Availability Section is restricted to new primary data that are part of this study. All primary datasets produced in this study need to be deposited in a appropriate public database and the accession numbers and database should be listed. Additional text should be removed.
- Please remove the list of abbreviations. Instead, please define each abbreviation the first time it appears in the text.
- We updated our journal's competing interests policy in January 2022 and request authors to consider both actual and perceived competing interests. Please review the policy https://www.embopress.org/competing-interests and update your competing interests if necessary. Kindly also update the title of this section to "Disclosure and competing interests statement".
- Funding: please add CNRS and INSERM in the submission system.
- Please remove the DOI from the references.

2/ Figures and Appendix:
- The excel table "Reagents List" needs to be renamed as "Dataset EV1", a legend should be added to the file and the callout in the manuscript should be updated.
- Appendix file: You may wish to make some of the supplementary figures as EV figures: We replaced Supplementary Information with Expanded View (EV) Figures and Tables that are collapsible/expandable online. A maximum of 5 EV Figures can be typeset. EV Figures should be cited as 'Figure EV1, Figure EV2" etc... in the text and their respective legends should be included in the main text after the legends of regular figures.
For the figures that you do NOT wish to display as Expanded View figures, they should be bundled together with their legends in a single PDF file called *Appendix*, which should start with a short Table of Content. Appendix figures should be referred to in the main text as: "Appendix Figure S1, Appendix Figure S2" etc.
Additional Tables/Datasets should be labelled and referred to as Table EV1, Dataset EV1, etc. Legends have to be provided in a separate tab in case of .xls files. Alternatively, the legend can be supplied as a separate text file (README) and zipped together with the Table/Dataset file.
See detailed instructions here: .
- The suppl. experimental procedures should be merged with the M&M in the main manuscript. Information on source data should be added to the corresponding source data files.
- Thank you for providing Source Data. Please upload them as one file per figure.

3/ For data quantification: please specify the name of the statistical test used to generate error bars and P values, the number (n) of independent experiments (specify technical or biological replicates) underlying each data point and the test used to calculate p-values in each figure legend. The figure legends should contain a basic description of n, P and the test applied. Graphs must include a description of the bars and the error bars (s.d., s.e.m.). Please provide exact p values.

4/ Please provide a complete author checklist, which you can download from our author guidelines (https://www.embopress.org/page/journal/17574684/authorguide#submissionofrevisions). Please insert information in the checklist that is also reflected in the manuscript. The completed author checklist will also be part of the RPF.

5/ Please note that all corresponding authors are required to supply an ORCID ID for their name upon submission of a revised manuscript. An ORCID identifier is currently missing for A. Bikfalvi.

6/ Please provide "The paper explained" section: EMBO Molecular Medicine articles are accompanied by a summary of the articles to emphasize the major findings in the paper and their medical implications for the non-specialist reader. Please provide a draft summary of your article highlighting
- the medical issue you are addressing,
- the results obtained and
- their clinical impact.
This may be edited to ensure that readers understand the significance and context of the research. Please refer to any of our published articles for an example.

7/ Author contributions: CRediT has replaced the traditional author contributions section because it offers a systematic machine readable author contributions format that allows for more effective research assessment. Please remove the Authors Contributions from the manuscript and use the free text boxes beneath each contributing author's name in our system to add specific details on the author's contribution. More information is available in our guide to authors.

8/ Please provide a synopsis as an individual file. You may use the graphical abstract provided in the rebuttal letter. The synopsis image should be uploaded as a PNG/TIF/JPEG file 550 px wide x 300-600 px high. Please note that referee #1 commented on the graphical abstract.

9/ As part of the EMBO Publications transparent editorial process initiative (see our Editorial at http://embomolmed.embopress.org/content/2/9/329), EMBO Molecular Medicine will publish online a Review Process File (RPF) to accompany accepted manuscripts.
This file will be published in conjunction with your paper and will include the anonymous referee reports, your point-by-point response and all pertinent correspondence relating to the manuscript. Let us know whether you agree with the publication of the RPF and as here, if you want to remove or not any figures from it prior to publication.
Please note that the Authors checklist will be published at the end of the RPF.

I look forward to receiving your revised manuscript.

With kind regards,

Lise

Lise Roth, PhD
Senior Editor
EMBO Molecular Medicine

***** Reviewer's comments *****

Referee #1 (Remarks for Author):

The authors adequately addressed all the concerns raised during the initial review. However, an edition of the graphical abstract (right panel) is recommended. Current graphical abstract illustrates dual LDHA/B inhibition result in changes in metabolic dysregulation that lead to reduced tumor growth and invasion. However, left and right cells are exactly identical. The readers may better understand the model if the authors could describe the changes that occur distinctively in glycolytic and oxidative cells by the loss of LDHA and LDHB, respectively.

Referee #2 (Comments on Novelty/Model System for Author):

This is a second-time revised manuscript which is further improved.

Referee #2 (Remarks for Author):

This is a second-time revised manuscript which is further improved. The authors addressed my concerns with new data and revised the text. The manuscript is sufficient for its furthr consideration in this journal.

Referee #3 (Remarks for Author):

The authors have answered all our questions and have substantially improved the manuscript

***

Rev_Com_number: RC-2021-00982
New_manu_number: EMM-2021-15343-V2
Corr_author: Daubon
Title: Lactate dehydrogenases promote glioblastoma growth and invasion via metabolic symbiosis

Final revision – EMM – Sept 2022

Thank you for the submission of your manuscript to EMBO Molecular Medicine, and please accept my apologies for the delay in getting back to you as I was out of office when the referees' reports came back. We have now received the enclosed reports from the three reviewers who had initially reviewed your manuscript. As you will see, they are now supportive of publication, and **I am therefore pleased to inform you that we will be able to accept your manuscript** once the following points will be addressed:

1/ Main manuscript text:

- Please answer/correct the changes suggested by our data editors in the main manuscript file (in track changes mode). This file will be sent to you in the next couple of days. Please use this file for any further modification.
→ This has been corrected.

- Thank you for providing the 'Data Availability' section. Please note that the Data Availability Section is restricted to new primary data that are part of this study. All primary datasets produced in this study need to be deposited in a appropriate public database and the accession numbers and database should be listed. Additional text should be removed.
→ This has been done.

- Please remove the list of abbreviations. Instead, please define each abbreviation the first time it appears in the text.
→ This has been corrected.

- We updated our journal's competing interests policy in January 2022 and request authors to consider both actual and perceived competing interests. Please review the policy https://www.embopress.org/competing-interests and update your competing interests if necessary. Kindly also update the title of this section to "Disclosure and competing interests statement".
→ This has been corrected.

- Funding: please add CNRS and INSERM in the submission system.
→ This has been added.

- Please remove the DOI from the references.
→ This has been corrected.

2/ Figures and Appendix:

- The excel table "Reagents List" needs to be renamed as "Dataset EV1", a legend should be added to the file and the callout in the manuscript should be updated.
→ This has been corrected.

- Appendix file: You may wish to make some of the supplementary figures as EV figures: We replaced Supplementary Information with Expanded View (EV) Figures and Tables that are collapsible/expandable online. A maximum of 5 EV Figures can be typeset. EV Figures should be cited as 'Figure EV1, Figure EV2" etc... in the text and their respective legends should be included in the main text after the legends of regular figures.

→ This has been corrected.
For the figures that you do NOT wish to display as Expanded View figures, they should be bundled together with their legends in a single PDF file called *Appendix*, which should start with a short Table of Content. Appendix figures should be referred to in the main text as: "Appendix Figure S1, Appendix Figure S2" etc.
→ This has been corrected.
Additional Tables/Datasets should be labelled and referred to as Table EV1, Dataset EV1, etc. Legends have to be provided in a separate tab in case of .xls files. Alternatively, the legend can be supplied as a separate text file (README) and zipped together with the Table/Dataset file.
See detailed instructions here:
https://www.embopress.org/page/journal/17574684/authorguide#expandedview.
→ This has been corrected.

- The suppl. experimental procedures should be merged with the M&M in the main manuscript. Information on source data should be added to the corresponding source data files.
→ This has been done.

- Thank you for providing Source Data. Please upload them as one file per figure.
→ This has been done.

3/ For data quantification: please specify the name of the statistical test used to generate error bars and P values, the number (n) of independent experiments (specify technical or biological replicates) underlying each data point and the test used to calculate p-values in each figure legend. The figure legends should contain a basic description of n, P and the test applied. Graphs must include a description of the bars and the error bars (s.d., s.e.m.). Please provide exact p values.
→ This has been corrected.

4/ Please provide a complete author checklist, which you can download from our author guidelines (https://www.embopress.org/page/journal/17574684/authorguide#submissionofrevisions). Please insert information in the checklist that is also reflected in the manuscript. The completed author checklist will also be part of the RPF.
→ This has been done.

5/ Please note that all corresponding authors are required to supply an ORCID ID for their name upon submission of a revised manuscript. An ORCID identifier is currently missing for A. Bikfalvi.
→ Andreas Bikfalvi has now an ORCID identifier: 0000-0003-4138-5229. This has been added to the author's information.

6/ Please provide "The paper explained" section: EMBO Molecular Medicine articles are accompanied by a summary of the articles to emphasize the major findings in the paper and their medical implications for the non-specialist reader. Please provide a draft summary of your article highlighting
- the medical issue you are addressing,
- the results obtained and
- their clinical impact.

This may be edited to ensure that readers understand the significance and context of the research. Please refer to any of our published articles for an example.

→ **Here is our "the paper explained" section**

PROBLEM

Lactate is a central metabolite in brain function but also in tumor development. Glioblastomas are very heterogeneous and severe brain tumors with minimal treatment for patients. Yet, a better understanding of how brain tumor cells adapt to oxygen constraints *via* metabolic rewiring is crucial.

RESULTS

Using multiple models such as immunostainings on 3D invasive spheroids or patient-derived tumors and spatial transcriptomics data on patient material, we described a regional expression of lactate dehydrogenase (LDH) A and B in glioblastoma, suggesting a metabolic symbiosis between glycolytic and oxidative cells through lactate transfer. Indeed, LDHA is more expressed in hypoxic areas, and a few invasive cells, and LDHB is expressed in oxygenated/vascularized and invasive regions. We then showed that lactate, massively produced in hypoxic areas, fuels TCA cycle to sustain the invasion and proliferation of glucose-starved cells. Only double LDHA/B KO led to a drastic decrease in tumor development and invasion. However, metabolic adaptation through respiratory chain remodeling and activity happened in double LDHA/B KO cells, even under low oxygen concentration. Thus, cranial radiation of the LDHA/B KO tumors further improved mouse survival. Finally, we repurposed an antiepileptic drug, stiripentol, to directly target LDHA and LDHB activity, which led to decreased cell respiration, proliferation, and invasion, and further increased survival in intracranially implanted mice.

IMPACT

Metabolic symbiosis in tumors is a central phenomenon to define good therapeutic targets to efficiently treat patients with glioblastoma. Finding drugs crossing the blood-brain barrier and having specific inhibition activity on major metabolic enzymes, such as LDHs, led to a massive decrease in glioblastoma development in mouse models. Potential clinical studies can be based on these promising results.

7/ Author contributions: CRediT has replaced the traditional author contributions section because it offers a systematic machine readable author contributions format that allows for more effective research assessment. Please remove the Authors Contributions from the manuscript and use the free text boxes beneath each contributing author's name in our system to add specific details on the author's contribution. More information is available in our guide to authors.

→ This has been added.

8/ Please provide a synopsis as an individual file. You may use the graphical abstract provided in the rebuttal letter. The synopsis image should be uploaded as a PNG/TIF/JPEG file 550 px wide x 300-600 px high. Please note that referee #1 commented on the graphical abstract.

→ This has been corrected. Comments from referee #1 have been considered to reshape the graphical abstract. The graphical abstract and its legend have been uploaded.

9/ As part of the EMBO Publications transparent editorial process initiative (see our Editorial at http://embomolmed.embopress.org/content/2/9/329), EMBO Molecular Medicine will

***** Reviewer's comments *****

Referee #1 (Remarks for Author):

The authors adequately addressed all the concerns raised during the initial review. However, an edition of the graphical abstract (right panel) is recommended. Current graphical abstract illustrates dual LDHA/B inhibition result in changes in metabolic dysregulation that lead to reduced tumor growth and invasion. However, left and right cells are exactly identical. The readers may better understand the model if the authors could describe the changes that occur distinctively in glycolytic and oxidative cells by the loss of LDHA and LDHB, respectively.
→ We would like to thank referee #1 for the positive comments. We have addressed the last question concerning the graphical abstract as seen below :

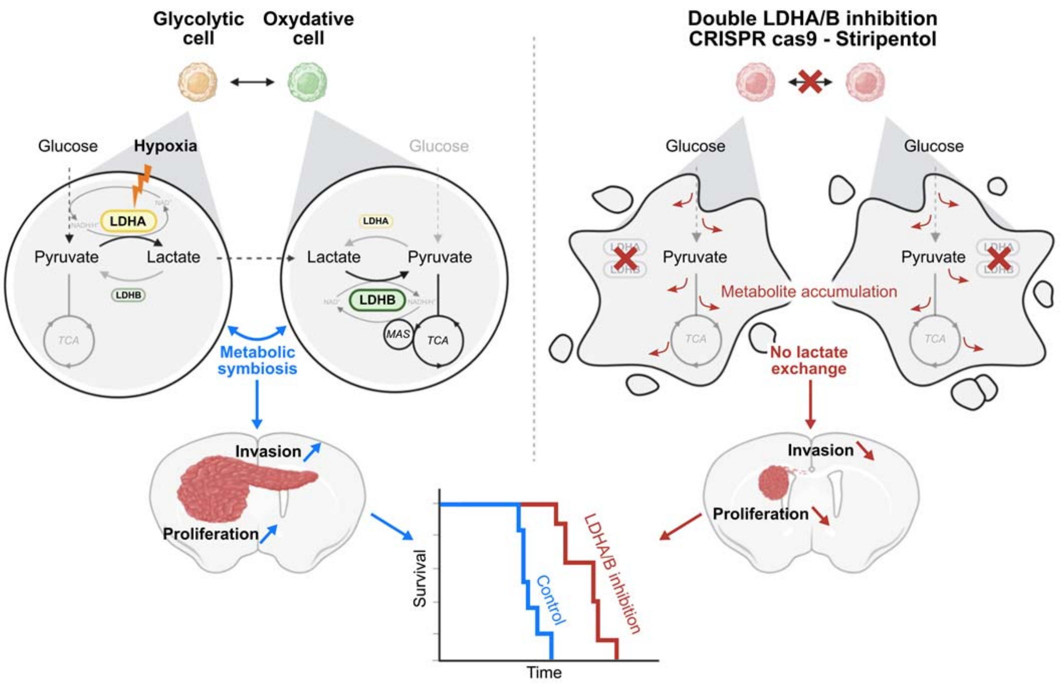

Referee #2 (Comments on Novelty/Model System for Author):

This is a second-time revised manuscript which is further improved.

Referee #2 (Remarks for Author):

This is a second-time revised manuscript which is further improved. The authors addressed my concerns with new data and revised the text. The manuscript is sufficient for its furthr consideration in this journal.

→ We would like to thank referee #2 for the positive comments.

Referee #3 (Remarks for Author):

The authors have answered all our questions and have substantially improved the manuscript.

→ We would like to thank referee #3 for these positive comments.

27th Sep 2022

Dear Thomas,

I am pleased to inform you that your manuscript is accepted for publication and is now being sent to our publisher to be included in the next available issue of EMBO Molecular Medicine!

Please read below for additional IMPORTANT information regarding your article, its publication and the production process.

Congratulations on your interesting work!

With kind regards,

Lise

Lise Roth, Ph.D
Senior Editor
EMBO Molecular Medicine

Follow us on Twitter @EmboMolMed
Sign up for eTOCs at embopress.org/alertsfeeds

EMBO Press Author Checklist

Corresponding Author Name: Thomas DAUBON	**USEFUL LINKS FOR COMPLETING THIS FORM**
Journal Submitted to: EMBO Molecular Medecine	The EMBO Journal - Author Guidelines
Manuscript Number: EMM-2021-15343	EMBO Reports - Author Guidelines
	Molecular Systems Biology - Author Guidelines
	EMBO Molecular Medicine - Author Guidelines

Reporting Checklist for Life Science Articles (updated January 2022)
This checklist is adapted from Materials Design Analysis Reporting (MDAR) Checklist for Authors. MDAR establishes a minimum set of requirements in transparent reporting in the life sciences (see Statement of Task: 10.31222/osf.io/9sm4x). Please follow the journal's guidelines in preparing your manuscript.
Please note that a copy of this checklist will be published alongside your article.

Abridged guidelines for figures
1. Data
The data shown in figures should satisfy the following conditions:
- the data were obtained and processed according to the field's best practice and are presented to reflect the results of the experiments in an accurate and unbiased manner.
- ideally, figure panels should include only measurements that are directly comparable to each other and obtained with the same assay.
- plots include clearly labeled error bars for independent experiments and sample sizes. Unless justified, error bars should not be shown for technical replicates.
- if n<5, the individual data points from each experiment should be plotted. Any statistical test employed should be justified.
- Source Data should be included to report the data underlying figures according to the guidelines set out in the authorship guidelines on Data Presentation.

2. Captions
Each figure caption should contain the following information, for each panel where they are relevant:
- a specification of the experimental system investigated (eg cell line, species name).
- the assay(s) and method(s) used to carry out the reported observations and measurements.
- an explicit mention of the biological and chemical entity(ies) that are being measured.
- an explicit mention of the biological and chemical entity(ies) that are altered/varied/perturbed in a controlled manner.
- the exact sample size (n) for each experimental group/condition, given as a number, not a range;
- a description of the sample collection allowing the reader to understand whether the samples represent technical or biological replicates (including how many animals, litters, cultures, etc.).
- a statement of how many times the experiment shown was independently replicated in the laboratory.
- definitions of statistical methods and measures:
  - common tests, such as t-test (please specify whether paired vs. unpaired), simple χ2 tests, Wilcoxon and Mann-Whitney tests, can be unambiguously identified by name only, but more complex techniques should be described in the methods section;
  - are tests one-sided or two-sided?
  - are there adjustments for multiple comparisons?
  - exact statistical test results, e.g., P values = x but not P values < x;
  - definition of 'center values' as median or average;
  - definition of error bars as s.d. or s.e.m.

Please complete ALL of the questions below.
Select "Not Applicable" only when the requested information is not relevant for your study.

Materials

Newly Created Materials	Information included in the manuscript?	In which section is the information available? (Reagents and Tools Table, Materials and Methods, Figures, Data Availability Section)
New materials and reagents need to be available; do any restrictions apply?	Not Applicable	/

Antibodies	Information included in the manuscript?	In which section is the information available? (Reagents and Tools Table, Materials and Methods, Figures, Data Availability Section)
For **antibodies** provide the following information:<br>- Commercial antibodies: RRID (if possible) or supplier name, catalogue number and or/clone number<br>- Non-commercial: RRID or citation	Yes	Dataset EV1

DNA and RNA sequences	Information included in the manuscript?	In which section is the information available? (Reagents and Tools Table, Materials and Methods, Figures, Data Availability Section)
**Short novel DNA or RNA including primers, probes**: provide the sequences.	Not Applicable	/

Cell materials	Information included in the manuscript?	In which section is the information available? (Reagents and Tools Table, Materials and Methods, Figures, Data Availability Section)
**Cell lines:** Provide species information, strain. Provide accession number in repository **OR** supplier name, catalog number, clone number, and/**OR** RRID.	Not Applicable	/
**Primary cultures:** Provide species, strain, sex of origin, genetic modification status.	Yes	Materials and Methods
Report if the cell lines were recently **authenticated** (e.g., by STR profiling) and tested for mycoplasma contamination.	Yes	Materials and Methods

Experimental animals	Information included in the manuscript?	In which section is the information available? (Reagents and Tools Table, Materials and Methods, Figures, Data Availability Section)
**Laboratory animals or Model organisms:** Provide species, strain, sex, age, genetic modification status. Provide accession number in repository **OR** supplier name, catalog number, clone number, **OR** RRID.	Yes	Materials and Methods
**Animal observed in or captured from the field:** Provide species, sex, and age where possible.	Not Applicable	/
Please detail **housing and husbandry conditions**.	Yes	Materials and Methods

Plants and microbes	Information included in the manuscript?	In which section is the information available? (Reagents and Tools Table, Materials and Methods, Figures, Data Availability Section)
**Plants:** provide species and strain, ecotype and cultivar where relevant, unique accession number if available, and source (including location for collected wild specimens).	Not Applicable	/
**Microbes:** provide species and strain, unique accession number if available, and source.	Not Applicable	/

Human research participants	Information included in the manuscript?	In which section is the information available? (Reagents and Tools Table, Materials and Methods, Figures, Data Availability Section)
If collected and within the bounds of privacy constraints report on age, sex and gender or ethnicity for all study participants.	Not Applicable	/

Core facilities	Information included in the manuscript?	In which section is the information available? (Reagents and Tools Table, Materials and Methods, Figures, Data Availability Section)
If your work benefited from core facilities, was their service mentioned in the acknowledgments section?	Yes	Acknowledgements section

Design

Study protocol	Information included in the manuscript?	In which section is the information available? (Reagents and Tools Table, Materials and Methods, Figures, Data Availability Section)
If study protocol has been **pre-registered, provide DOI in the manuscript**. For clinical trials, provide the trial registration number **OR** cite DOI.	Yes	doi: 10.21203/rs.3.rs-690811/v1
Report the **clinical trial registration number** (at ClinicalTrials.gov or equivalent), where applicable.	Not Applicable	/

Laboratory protocol	Information included in the manuscript?	In which section is the information available? (Reagents and Tools Table, Materials and Methods, Figures, Data Availability Section)
Provide DOI OR other citation details if **external detailed step-by-step protocols** are available.	Yes	NADH-independent enzymatic assay to quantify extracellular and intracellular L-lactate levels. Bouchez CL, Daubon T, Mourier A. STAR Protoc. 2022 May 16;3(2):101403.

Experimental study design and statistics	Information included in the manuscript?	In which section is the information available? (Reagents and Tools Table, Materials and Methods, Figures, Data Availability Section)
Include a statement about **sample size** estimate even if no statistical methods were used.	Yes	Figure legends
Were any steps taken to minimize the effects of subjective bias when allocating animals/samples to treatment (e.g. **randomization procedure**)? If yes, have they been described?	Yes	Materials and Methods
Include a statement about **blinding** even if no blinding was done.	Yes	Materials and Methods
Describe **inclusion/exclusion criteria** if samples or animals were excluded from the analysis. Were the criteria pre-established?<br><br>If sample or data points were omitted from analysis, report if this was due to attrition or intentional exclusion and provide justification.	Yes	Materials and Methods
For every figure, are **statistical tests** justified as appropriate? Do the data meet the assumptions of the tests (e.g., normal distribution)? Describe any methods used to assess it. Is there an estimate of variation within each group of data? Is the variance similar between the groups that are being statistically compared?	Yes	Figure legends and Tables EVs

Sample definition and in-laboratory replication	Information included in the manuscript?	In which section is the information available? (Reagents and Tools Table, Materials and Methods, Figures, Data Availability Section)
In the figure legends: state number of times the experiment was **replicated** in laboratory.	Yes	Figure legends and Tables EVs
In the figure legends: define whether data describe **technical or biological replicates**.	Yes	Figure legends and Tables EVs

Ethics

Ethics	Information included in the manuscript?	In which section is the information available? (Reagents and Tools Table, Materials and Methods, Figures, Data Availability Section)
Studies involving **human participants**: State details of **authority granting ethics approval** (IRB or equivalent committee(s), provide reference number for approval.	Not Applicable	/
Studies involving **human participants**: Include a statement confirming that **informed consent** was obtained from all subjects and that the experiments conformed to the principles set out in the WMA Declaration of Helsinki and the Department of Health and Human Services Belmont Report.	Not Applicable	/
Studies involving **human participants:** For publication of **patient photos**, include a statement confirming that consent to publish was obtained.	Not Applicable	/
Studies involving experimental **animals**: State details of **authority granting ethics approval** (IRB or equivalent committee(s), provide reference number for approval. Include a statement of compliance with ethical regulations.	Yes	Materials and Methods
Studies involving **specimen and field samples:** State if relevant **permits** obtained, provide details of authority approving study; if none were required, explain why.	Not Applicable	/

Dual Use Research of Concern (DURC)	Information included in the manuscript?	In which section is the information available? (Reagents and Tools Table, Materials and Methods, Figures, Data Availability Section)
Could your study fall under dual use research restrictions? Please check biosecurity documents and list of **select agents and toxins** (CDC): https://www.selectagents.gov/sat/list.htm	Not Applicable	/
If you used a select agent, is the security level of the lab appropriate and reported in the manuscript?	Not Applicable	/
If a study is subject to dual use research of concern regulations, is the name of the **authority granting approval and reference number** for the regulatory approval provided in the manuscript?	Not Applicable	/

Reporting

The MDAR framework recommends adoption of discipline-specific guidelines, established and endorsed through community initiatives. Journals have their own policy about requiring specific guidelines and recommendations to complement MDAR.

Adherence to community standards	Information included in the manuscript?	In which section is the information available? (Reagents and Tools Table, Materials and Methods, Figures, Data Availability Section)
State if relevant guidelines or checklists (e.g., **ICMJE, MIBBI, ARRIVE, PRISMA**) have been followed or provided.	Not Applicable	/
For **tumor marker prognostic studies,** we recommend that you follow the **REMARK** reporting guidelines (see link list at top right). See author guidelines, under 'Reporting Guidelines'. Please confirm you have followed these guidelines.	Not Applicable	/
For **phase II and III randomized controlled trials**, please refer to the **CONSORT** flow diagram (see link list at top right) and submit the CONSORT checklist (see link list at top right) with your submission. See author guidelines, under 'Reporting Guidelines'. Please confirm you have submitted this list.	Not Applicable	/

Data Availability

Data availability	Information included in the manuscript?	In which section is the information available? (Reagents and Tools Table, Materials and Methods, Figures, Data Availability Section)
Have **primary datasets** been deposited according to the journal's guidelines (see 'Data Deposition' section) and the respective accession numbers provided in the Data Availability Section?	Yes	Data availability section
Were **human clinical and genomic datasets** deposited in a public access-controlled repository in accordance to ethical obligations to the patients and to the applicable consent agreement?	Not Applicable	/
Are **computational models** that are central and integral to a study available without restrictions in a machine-readable form? Were the relevant accession numbers or links provided?	Not Applicable	/
If publicly available data were reused, provide the respective **data citations in the reference list**.	Yes	References
